# Genetic association studies of alterations in protein function expose recessive effects on cancer predisposition

**DOI:** 10.1038/s41598-021-94252-y

**Published:** 2021-07-21

**Authors:** Nadav Brandes, Nathan Linial, Michal Linial

**Affiliations:** 1 The Rachel and Selim Benin School of Computer Science and Engineering, The Hebrew University of Jerusalem, Jerusalem, Israel; 2 Department of Biological Chemistry, The Alexander Silberman Institute of Life Science, The Hebrew University of Jerusalem, Jerusalem, Israel

**Keywords:** Cancer genetics, Genetic association study

## Abstract

The characterization of germline genetic variation affecting cancer risk, known as cancer predisposition, is fundamental to preventive and personalized medicine. Studies of genetic cancer predisposition typically identify significant genomic regions based on family-based cohorts or genome-wide association studies (GWAS). However, the results of such studies rarely provide biological insight or functional interpretation. In this study, we conducted a comprehensive analysis of cancer predisposition in the UK Biobank cohort using a new gene-based method for detecting protein-coding genes that are functionally interpretable. Specifically, we conducted proteome-wide association studies (PWAS) to identify genetic associations mediated by alterations to protein function. With PWAS, we identified 110 significant gene-cancer associations in 70 unique genomic regions across nine cancer types and pan-cancer. In 48 of the 110 PWAS associations (44%), estimated gene damage is associated with reduced rather than elevated cancer risk, suggesting a protective effect. Together with standard GWAS, we implicated 145 unique genomic loci with cancer risk. While most of these genomic regions are supported by external evidence, our results also highlight many novel loci. Based on the capacity of PWAS to detect non-additive genetic effects, we found that 46% of the PWAS-significant cancer regions exhibited exclusive recessive inheritance. These results highlight the importance of recessive genetic effects, without relying on familial studies. Finally, we show that many of the detected genes exert substantial cancer risk in the studied cohort determined by a quantitative functional description, suggesting their relevance for diagnosis and genetic consulting.

## Introduction

Cancer is considered a genetic disease. Notably, that characterization typically refers to somatic mutations in tumors, not to inherited germline genetic variation. Nonetheless, like with any non-Mendelian disease, germline variants are known to contribute to cancer predisposition^1^.

The best-known examples of cancer predisposition genes are the tumor suppressors BRCA1 and BRCA2 involved in breast and ovarian cancers^2^. By 2014, following thirty years of research, 114 cancer predisposition genes had been reported, mostly based on families with high prevalence of cancer^3^. As of 2016, the list was expanded to include genes from pediatric cancers^4^. Some of the genes most prevalent in children are also known cancer driver genes from the somatic context (e.g. TP53, APC, BRCA2, NF1, PMS2, RB1 and RUNX1). Recently, a based unified list of 152 cancer predisposition genes was refined on germline variants from The Cancer Genome Atlas (TCGA) across 33 cancer types^5^ (see also the discussion in^6^).

Although the heritability of most cancers is considerable^7–9^ and despite many years of research leading to the identification of numerous genes, the overall genetic risk explained by the discovered genes remains quite limited. It turned out that BRCA1 and BRCA2 are quite exceptional in their high penetrance, with most cancer predisposition genes showing only mild effects^10–13^. Notably, a gene’s effect size is highly dependent on the cancer-type and population context. For example, BRCA1 and BRCA2 are mostly specific to a small set of cancer types such as breast and ovarian cancer, and are highly enriched in Ashkenazi Jews^2^.

In recent years, the exponential growth in genotyped cohorts and the availability of genome-wide association studies (GWAS) have allowed the discovery of additional genetic loci associated with cancer. As opposed to family studies, GWAS can pick genetic factors with smaller effect sizes, provided that the variants are common enough. Several years of GWAS research into cancer predisposition have implicated numerous cancer predisposition loci (e.g. in breast^14,15^, lung^16^, prostate^17^, colorectal cancer^18^ and melanoma^19^). It is anticipated that by increasing cohort sizes, more genetic associations will be found. Interestingly, the overlaps of significant loci in independent GWAS works studying the same cancer types tend to be surprisingly small, supporting the claim that GWAS efforts in this domain are far from saturated^20,21^. Benefits from GWAS are also constrained by the lack of interpretability of most implicated loci, which do not appear to affect any functional elements in the genome^22^.

The majority of GWAS projects are restricted to specific cancer types, limiting their capacity to draw strong conclusions about the similarities and differences between the genetic signatures of cancer types. Moreover, the collective study of all cancer types as a single phenotype, known as pan-cancer, is prevalent in the somatic world^23^, but remains understudied in the germline context^24^. The UK Biobank (UKB) cohort^25,26^ is highly suitable for comparative studies between phenotypes, due to its uniform standards and protocols applied across all samples. The UKB covers ~ 500,000 genotyped individuals with open medical records. As it recruited only individuals older than 40 years, the cohort is enriched with cancer cases^24^.

An important limitation of GWAS is its assumption of additive genetic effects, which leads to oversight of dominant and recessive effects. This limitation is particularly relevant to recessive inheritance. While some GWAS works have modeled recessive inheritance at the variant level, this solves only a small part of the problem, as recessive effects are likely to occur at the gene level. Specifically, it is anticipated that most recessive effects would manifest through compound heterozygosity, namely different variants affecting the two copies of the same gene^27,28^. Unfortunately, variant-level approaches like GWAS cannot detect genetic patterns that transcend individual variants. As attested by family studies, recessive inheritance is likely to play a major role in cancer predisposition^29–31^, as in other complex traits^32^.

**Figure 1:**
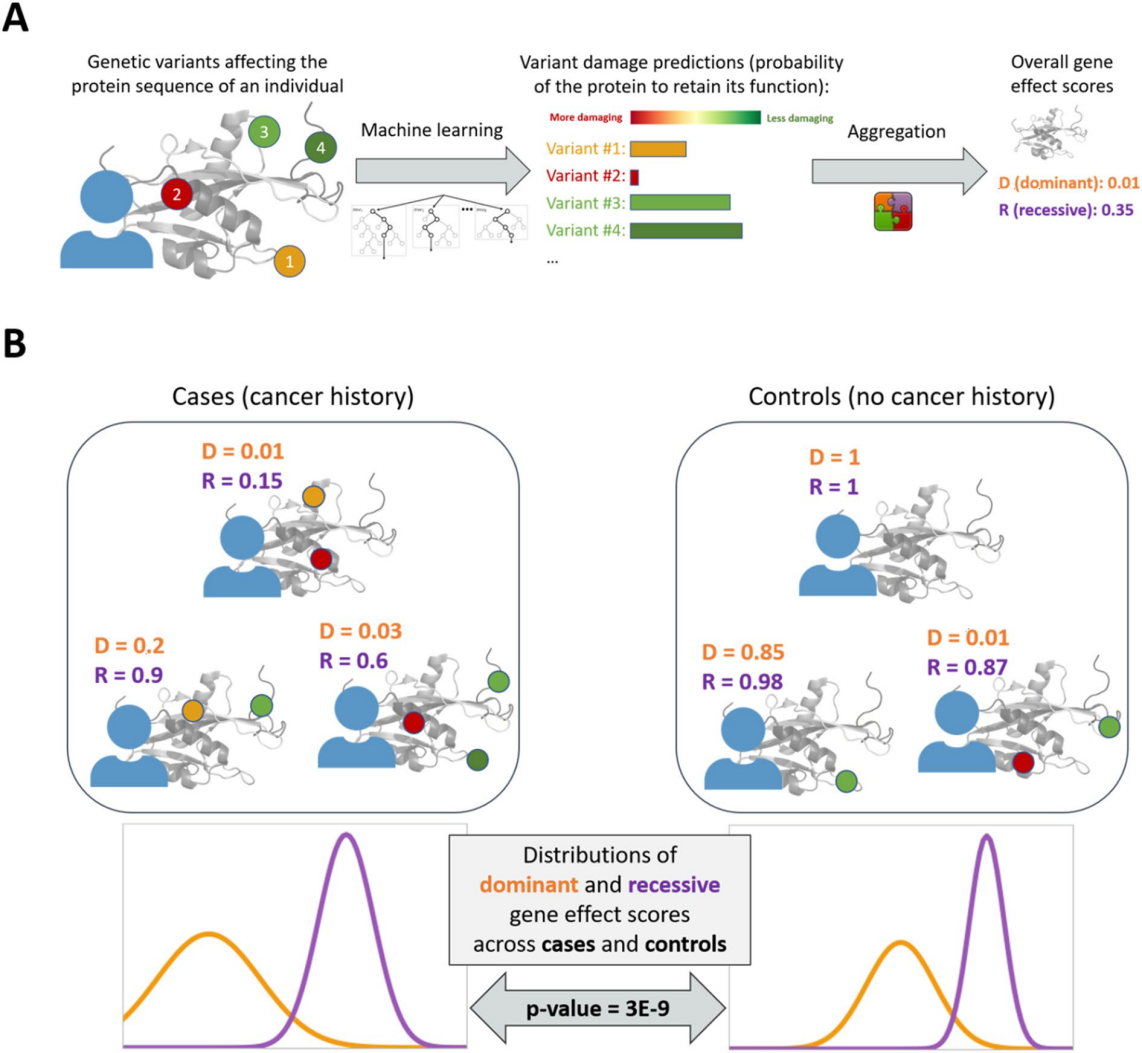
Proteome-wide association study (PWAS). PWAS finds associations between phenotypes (such as cancer) and protein-coding genes that are mediated by genetic alterations to protein function. (**A**) When studying a protein-coding gene, PWAS collects all the called variants in the genome of an individual that affect the coding sequence of that protein. Using a machine-learning model, it predicts how damaging these variants are to the protein function (for example, how likely an enzyme is to retain its catalytic activity given each of the mutations, which might affect its 3D structure). It then aggregates the predictions made for all the variants into overall gene effect scores. Each gene is assigned two such scores, corresponding to either a dominant or a recessive genetic effect. The dominant effect score attempts to capture the event that the gene is affected by at least one damaging variant, whereas the recessive effect score attempts to capture the event that it is affected by at least two damaging variants. (**B**) Given a cohort of cases and controls, PWAS assigns each individual dominant and recessive effect scores for the studied protein-coding gene based on his or her genetics, as described in (**A**). By comparing the distributions of gene effect scores assigned to cases and controls, PWAS can detect statistically significant differences between the two groups (measured by *p*-values and FDR q-values). These differences reflect the estimated functional damage to the gene, finding, for example, that the studied protein appears to be more damaged in cases than in controls.

Recently, we developed Proteome-Wide Association Study (PWAS)^33^, a new gene-based method that addresses many of the shortcomings of GWAS. PWAS detects gene-phenotype associations that are mediated by alterations to protein function (Fig. 1). To do so, it considers the proteomic context of genetic variants and their functional effects. As a gene-based method, PWAS aggregates the signal from all variants affecting the same protein-coding gene, and can detect genes with dominant and recessive effects. Unlike GWAS, PWAS associations are supported by concrete functional effects in coding genes, making them more interpretable.

In this work we study genetic predisposition to cancer in the UKB across 10 cancer types (including pan-cancer) using genome-wide and proteome-wide association studies, thereby providing a systematic view of genetic cancer predisposition from a protein function perspective. We compare our results to contemporary clinical knowledge about cancer predisposition and to catalogues of cancer driver genes from the somatic world. We took advantage of the capacity of PWAS to model dominant and recessive effects to systematically study the importance of these heritability modes in cancer predisposition across the human coding genome.

## Results

### Genetic and protein-function analysis of cancer predisposition in the UK Biobank

**Figure 2:**
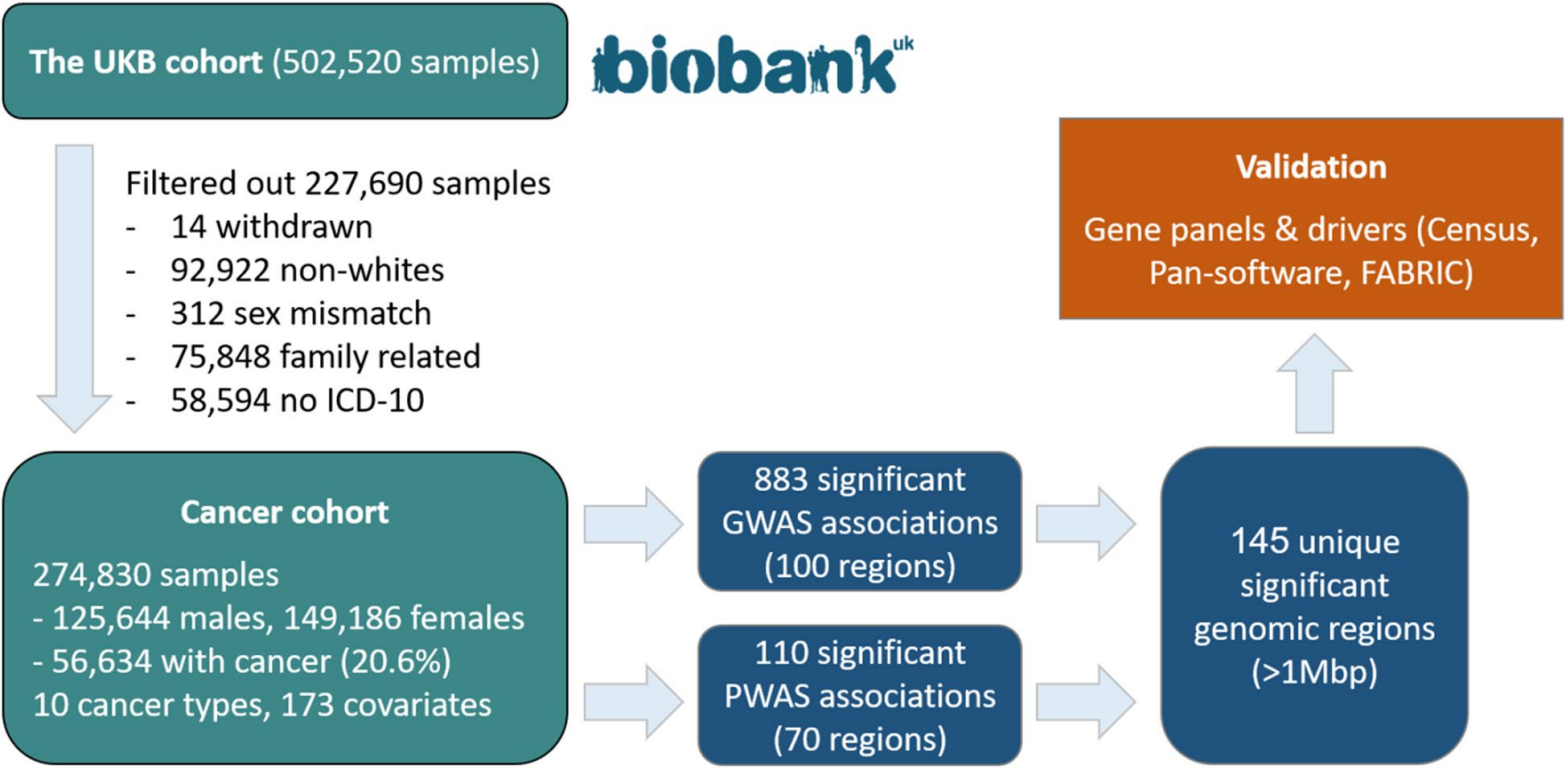
Analysis workflow. From the UK Biobank cohort (~ 500 K individuals), we derived a study cohort of 274,830 individuals. Based on their medical records, we determined whether each of the individuals had a history of cancer, focusing on nine major cancer types and pan-cancer which combines individuals with any cancer (56,634 overall). We used two statistical methods, standard GWAS and PWAS, over each of the ten cancer types, and found 883 variant-cancer and 110 gene-cancer significant associations, respectively. We merged the significant associations into 145 unique genomic regions (of at least 1,000,000 base pairs each), and validated the regions by comparing them with prominent gene panels used in the clinics and catalogues of cancer driver genes.

From the UKB, we derived a study cohort of 274,830 unrelated white individuals (Fig. 2). Based on their medical records, we determined that 56,634 of those individuals had a history of cancer. In addition to this pan-cancer view, we also considered nine distinct cancer types: melanoma, leukemia, breast, ovarian, prostate, lung, skin, pancreatic and colorectal cancer. We studied each of the ten defined cancer types (the nine specific cancers and pan-cancer) using both GWAS and PWAS independently. With GWAS we found 883 significant variant-cancer associations in 100 unique genomic regions, and with PWAS we found 110 significant gene-cancer associations in 70 unique genomic regions. Altogether, the GWAS and PWAS associations mapped to 145 unique genomic regions, available at Supplementary Table S1. The full GWAS and PWAS results across the ten cancer types and all tested variants and genes are available at Supplementary Table S2 and Supplementary Table S3.

**Figure 3:**
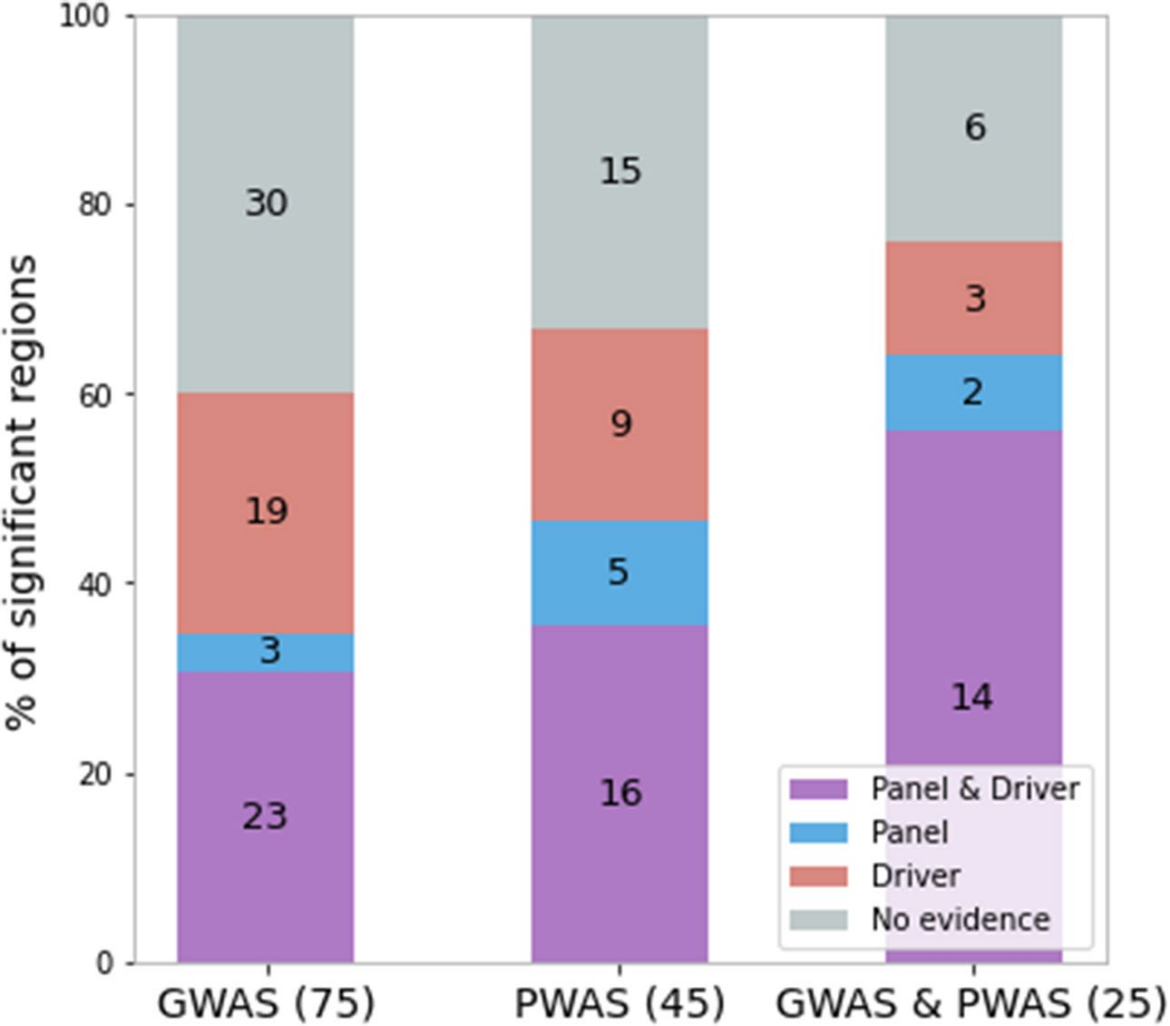
External evidence supporting the cancer predisposition regions. Partitioning of the 145 significant cancer genomic regions according to which method found them (GWAS, PWAS or both) and supporting evidence (overlap with clinical cancer gene panels, driver cancer genes, both, or none).

We compared the 145 discovered genomic regions against prominent cancer predisposition gene panels used in the clinics (CleanPlex TMB 500 and Invitae Multi-Cancer). We also compared our results to three catalogues of cancer driver genes: Census^34^, the pan-software catalogue^35^ and FABRIC^36,37^. We find that a majority of the 145 significant cancer regions are indeed supported by external evidence (clinical panels, cancer driver catalogues, or both), but 51 of the 145 regions (35%) appear as new discoveries (Fig. 3). We also observe that external validation is slightly stronger for significant regions that are supported by both GWAS and PWAS.

### Single-gene loci with direct interpretation

To demonstrate the discovered genetic signal of cancer predisposition, we begin by focusing on high-confidence associations with plausible causal mechanisms. One of the benefits of PWAS is its capacity to suggest concrete protein-coding genes underlying genetic associations. However, like GWAS, PWAS is also sensitive to linkage disequilibrium, which can lead to the genetic signal being spread across an extended genomic region and mistakenly attributed to an unrelated nearby gene. For this reason, it is of interest to examine significant associations that are restricted to single genes. We define a significant cancer region to be a soliton region if i) it has exactly one gene significant by PWAS, and ii) the significance is very strong (FDR q-value < 1E−03). Of the 145 cancer regions, 14 are solitons according to this definition (Table 1).

**Table 1:** Soliton cancer genes discovered by PWAS.

Gene	Chromosome bands	Significant cancer types	Lowest PWAS FDR q-value	Lowest GWAS *p*-value	PWAS heritability	Protein damage associated with cancer risk (R) or protection (P)	External evidence (out of the 2 gene panels and 3 cancer driver catalogues)
MUTYH	1p34.1	Colorectal	2E−7	1E−3	Recessive	R	CleanPlex, Invitae, Census
CTLA4	2q33.2-q33.3	Skin	4E−6	2E−13*	Both	P	CleanPlex
MITF	3p14.1-p13	Melanoma	1E−8	4E−4	Both	R	CleanPlex, Invitae, Census
SLC45A2	5p13.3-p13.2	Skin, melanoma, P-C	1E−20	4E−22*	Recessive	R	None
CCDC170	6q25.1-q25.2	Breast	5E−5	1E−13*	Dominant	R	None
POU5F1B	8q24.21	Prostate, P-C, colorectal, breast	1E−22	4E−42*	Both	P	None
MTAP	9p21.3	Skin, melanoma, P-C	1E−4	8E−23*	Both	P	None
STN1	10q24.33-q25.1	P-C, melanoma	9E−6	3E−10*	Recessive	P	None
UBE3A	15q11.2-q12	Pancreatic	8E−4	3E−4	Recessive	R	None
HOXB13	17q21.32	Prostate, P-C	2E−31	6E−40*	Dominant	R	CleanPlex, Invitae
IRF3	19q13.33	Skin	7E−6	1E−6	Dominant	R	None
KLK3	19q13.33-q13.41	Prostate	4E−9	3E−25*	Dominant	P	None
AVP	20p13	Melanoma	9E−4	2E−4	Recessive	P	None
CHEK2	22q12.1-q12.2	Breast, P-C, prostate	5E−5	3E−6	Dominant	R	CleanPlex, Invitae, Census, PanSoftware, FABRIC

*Below the GWAS exome-wide significance threshold (5E−07); P-C, Pan-cancer.

5 of the 14 soliton genes are supported by gene panels (CleanPlex or Invitae), and 3 of them are supported by Census and other cancer driver catalogues. Importantly, the criteria of external evidence for the solitons listed in Table 1 are stricter than those used for the genomic regions in Fig. 3. Whereas in Fig. 3 we considered evidence for any gene within the same region (which may include up to 138 genes; see Supplementary Table S1), in Table 1 we only consider evidence matching the exact soliton gene.

In addition to recovering genomic regions that are missed by GWAS, a major contribution of PWAS is highlighting the exact genes that drive known associations. Of the 14 soliton genes, 8 are also found by standard GWAS, whereas the other 6 are below the exome-wide significance threshold (5E−07). IRF3 (interferon regulatory factor 3), for example, is associated with non-melanoma skin cancer according to PWAS with overwhelming significance (FDR q-value = 7E−6), but not according to GWAS (*p* = 1E−6). IRF3 is part of the innate immune system, whose one of its major roles is to recognize and destroy infected and tumorous cells. In-vivo and expression studies have implicated IRF3 in melanoma^38,39^ and other cancer types^40,41^.

The list of solitons also includes novel discoveries. For example, UBE3A (ubiquitin protein ligase E3A; also known as E6AP) is known for resulting in the ubiquitylation and degradation of the p53 tumor suppressor in papillomavirus-positive cervical cancer^42^, and it has also been implicated with prostate and other cancers^43–45^. However, E6AP hasn’t been linked to pancreatic cancer, an association that we observe here (PWAS q-value = 8E−04). Another example is AVP (arginine vasopressin; also known as ADH), a gene producing a hormone involved in water balance homeostasis and complex behavioral traits^46^. Despite vast literature about the hormone, there doesn’t appear to be any direct evidence linking the gene to cancer or specifically to melanoma, as observed here (PWAS q-value = 9E−04).

### Functional damage to coding genes is often associated with reduced cancer risk

An advantage of PWAS is that it provides clear interpretation of the directionality of significant associations, namely whether gene damage is associated with elevated or reduced cancer risk. Of the 14 solitons, which are high-confidence PWAS associations, 8 genes (57%) are associated with elevated and 6 (43%) are associated with reduced cancer risk (Table 1). For example, CTLA4 (also known as CD152) is an immune checkpoint and a preferred target in cancer immunotherapy. Reduced CTLA4 function, either through natural genetic variation or immunotherapy treatment, activates T cells and suppresses tumor (while, on the downside, increases the risk for autoimmunity)^47^. Therefore, it is not surprising that CTLA4 is characterized by PWAS as a protective gene, with gene damage being associated with reduced cancer prevalence in the UKB cohort.

The characterization of genes as being associated with elevated or reduced cancer risk according to PWAS is based on either the dominant or recessive effect scores calculated by the method, depending on the heritability found significant for each association. Notably, the 4 soliton genes showing both dominant and recessive inheritance (potentially with different effect sizes and significance levels) all exhibit consistent trends. In other words, when estimated damage to these genes is associated with elevated (or reduced) cancer risk according to the dominant model, it shows the same trend according to the recessive model.

Across all 110 significant PWAS associations we observe similar patterns, with 62 (56%) being associated with elevated cancer risk and the other 48 (44%) with reduced risk. Among the 16 PWAS associations that are significant for both dominant and recessive effects, 15 show consistent directionality and only one association shows opposite trends between the dominant and recessive models.

### Genetic signal varies between cancers

**Figure 4:**
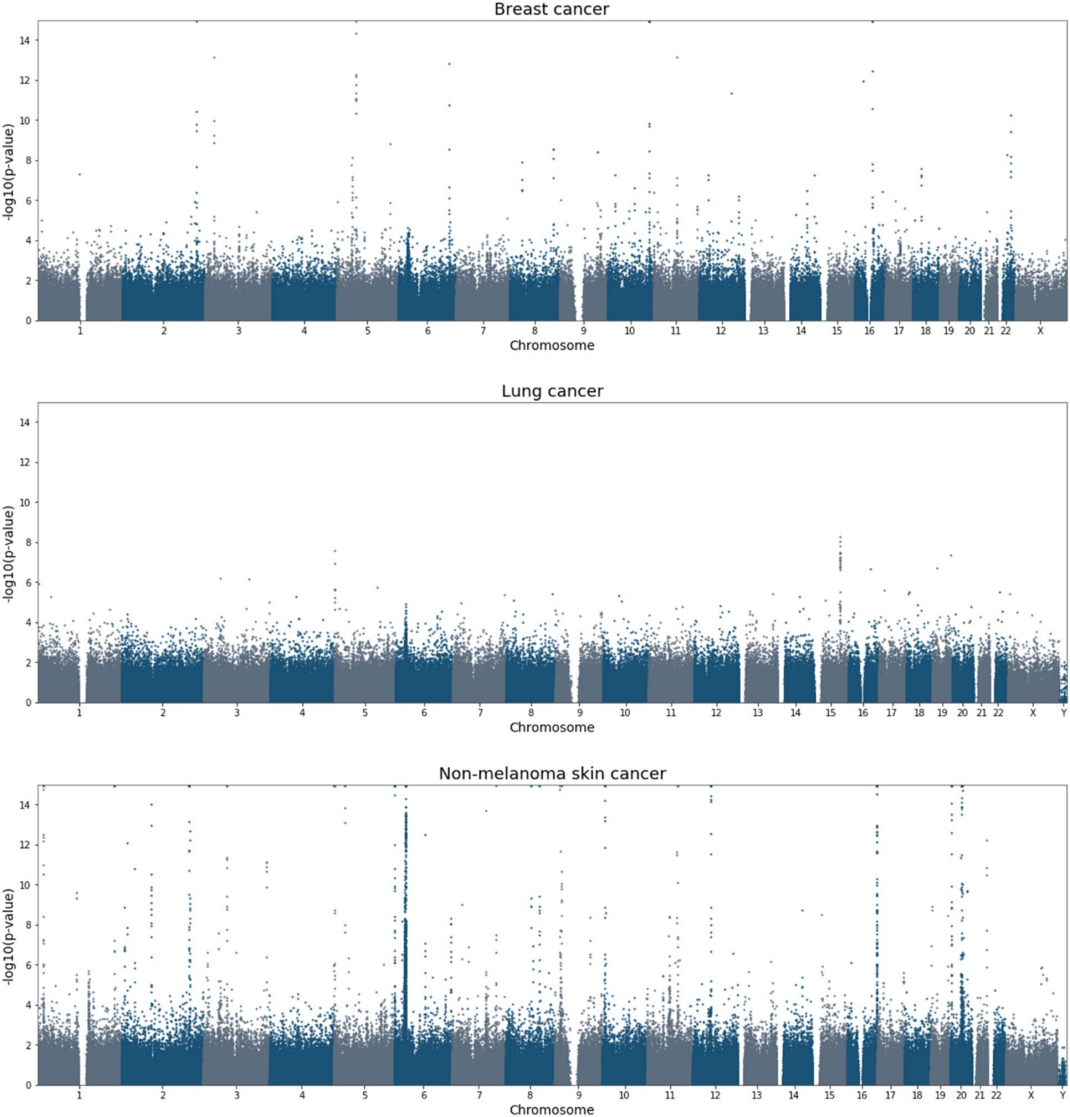
Genome-wide cancer predisposition in breast, lung and non-melanoma skin cancer. Manhattan plots for three selected cancer types. The plots show the significance of all the variants and genes tested with GWAS and PWAS. *p*-values are capped at 1E-15. Similar Manhattan plots for all ten cancer types (including pan-cancer) are available in Supplementary Fig. S1.

The uniformity of the UKB as a prospective cohort that covers all major cancer types provides a unique opportunity to perform a comparative analysis between cancer types. We start by considering the number of significant genomic regions recovered in each cancer type, according to GWAS, PWAS or both (Table 2). We observe that the number of recovered regions vary substantially across cancer types, ranging from 2 in epithelial ovarian cancer to 58 in non-melanoma skin cancer. As statistical power is determined by sample sizes, it is not surprising that cancers with fewer cases tend to recover fewer significant regions. However, this is not the only factor. Chronic lymphocytic leukemia, for example, has the fewest cases (764), yet it recovers 20 significant regions, more than many other cancers. This could be explained by a strong polygenic signal associated with leukemia, or more likely, it could be a spurious effect of mistaking somatic mutations as germline variants in blood cancers (as UKB genotypes are based on blood samples)^48^. Colorectal cancer includes 3351 cases but only 4 recovered regions, compared for example to melanoma with 14 recovered regions, despite its slightly lower number of cases (3122). Interestingly, although pan-cancer has the highest number of cases (by definition), it doesn’t recover the highest number of genomic regions. The comparison between cancer types also reinforces the contribution of both GWAS and PWAS to our analysis, with each of the two methods dominating the discovery in certain cancer types. Major differences in polygenic signal between cancer types are also observed with respect to significance levels (Fig. 4). For example, the 6 significant regions of lung cancer are statistically weaker than the 18 significant regions of breast cancer, although this could simply reflect the different numbers of cases.

**Table 2:** Studied cancer types.

Cancer type	ICD-10 codes	Sex	# samples	# cases	# controls	# significant GWAS regions	# significant PWAS regions	# total significant regions
Pan-cancer	C00-C26, C30-C34, C37-C58, C60-C86, C88, C90-C97	All	274,830	56,634	218,196	19	12	26
Breast cancer	C50	F	149,186	10,682	138,504	17	2	18
Chronic lymphocytic leukemia	C91	All	274,830	764	272,079	4	16	20
Colorectal cancer	C18	All	274,830	3351	271,479	1	4	4
Epithelial ovarian cancer	C56	F	149,186	1112	147,944	2	0	2
Lung cancer	C34	All	274,830	2479	272,351	3	4	6
Melanoma	C43	All	274,830	3122	271,708	8	12	14
Non-melanoma skin cancer	C44	All	274,830	18,944	255,886	43	27	58
Pancreatic cancer	C25	All	274,830	768	264,559	3	2	5
Prostate cancer	C61	M	125,644	7438	118,206	30	8	32

Among the 145 significant cancer predisposition loci, 65 are associated with melanoma or non-melanoma skin cancer. Genes affecting skin pigmentation were implicated with both cancer types. Of all the PWAS associations recovered in this study, the most significant is the gene MC1R (Melanocortin 1 receptor; q-value = 1E−109 in non-melanoma skin cancer and q-value = 2E−53 in melanoma), which plays a central role in skin pigmentation, melanin formation and melanocyte differentiation. Many variants in the gene have been associated with pigmentation differences and elevated risk of skin cancer and melanoma^49^. Additional major pigmentation-related genes identified in this study include TYR (tyrosinase; PWAS q-value = 6E−13 in non-melanoma skin cancer and q-value = 0.005 in melanoma) and SLC45A2 (solute carrier family 45 member 2; PWAS q-value = 1E−20 in non-melanoma skin cancer and q-value = 8E−5 in melanoma)^50^.

### Predisposition genomic loci are mostly cancer-type specific

**Figure 5:**
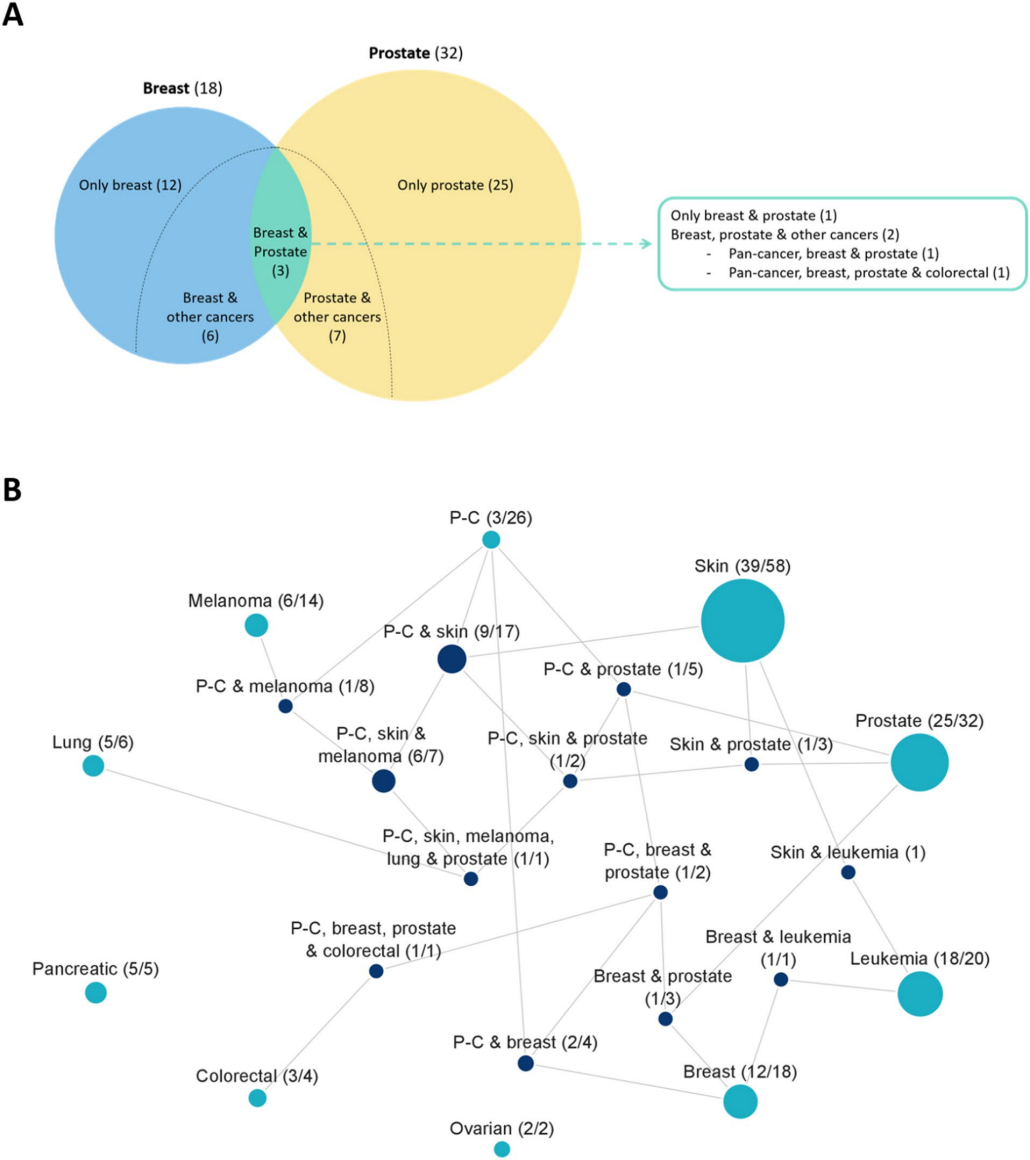
Number of significant regions per cancer-type combination. (**A**) Venn diagram of the 18 significant genomic regions in breast cancer and the 32 significant regions in prostate cancer with 3 overlapping regions between these two cancer types, as an example of the unique and shared genetic signals between cancer types. (**B**) Partitioning of the 145 significant cancer genomic regions by the combination of cancer types in which they were found significant. Each node in the graph represents a combination of cancer types. Two nodes are connected when one combination is a direct subset of the other. Each node is labeled with two numbers: the number of significant regions matching the exact combination of cancer types represented by the node (e.g. 12 genomic regions that are unique to breast cancer), and the total number of significant regions matching any combination of cancer types that includes this combination (e.g. overall 18 regions associated with breast cancer, including 6 regions shared with other cancers). Node sizes reflect the number of significant regions that match the combinations exactly. Nodes representing combinations of more than one cancer type are colored dark blue. Pan-cancer is abbreviated as P-C.

We also consider the extent to which cancer predisposition genetic signal overlaps between cancers, by counting the number of significant cancer regions shared between cancer types (Fig. 5). For example, melanoma and non-melanoma skin cancer share a substantial genetic signal, with 7 of the 14 melanoma regions being also significant in non-melanoma skin cancer (Fig. 5B). A shared genetic signal between these two cancer types is expected, given their relatedness. More surprising is the fact that 3 of the 18 breast-cancer regions are shared with prostate cancer (with overall 32 regions), while none of the 2 significant ovarian-cancer regions is shared with breast cancer (or any other cancer type). One of the 3 genomic regions shared between breast and prostate cancer is found in 11q13.3. Most significant in this region is the intergenic variant rs7130881 (lowest GWAS *p*-value = 3E−36). The other 2 regions shared between breast and prostate cancer contain soliton genes listed in Table 1: CHEK2 (22q12.1-q12.2) and POU5F1B (8q24.21). All six associations (the three regions with respect to the two cancer types) have strong support in literature^51–54^.

We observe that the pan-cancer phenotype does not substantially add to the recovery of significant genomic regions over the nine specific cancer types. Of the 26 significant pan-cancer regions, only 3 are not recovered by any of the specific cancer types. From a statistical perspective, this stands out given the high number of pan-cancer cases (56,634).

The genomic region with the most dominant pan-cancer signal is 5p15.33, which is also significant in non-melanoma skin cancer, melanoma, prostate cancer and lung cancer. Among the most significant variants in this region are rs401681 (GWAS *p*-value = 7E−32) in the intronic region of the gene CLPTM1L (Cisplatin Resistance Related Protein), and rs2853677 (GWAS *p*-value = 9E−22) in the intronic region of TERT (Telomerase Reverse Transcriptase). TERT is a well-known cancer predisposition gene implicated in numerous cancer types^55^. Mutations in TERT can cause abnormal activities of the telomerase and prevent telomere shortening, thereby leading to cell immortality. rs2853677 has been implicated in various Chinese and Indian populations^56,57^. We provide evidence for the importance of this variant in the white British population. CLPTM1L has also been linked to cancer in multiple studies. For example, overexpression of the gene has been shown to enhance the growth of pancreatic cancer cells in vitro and in vivo^58^.

Another relatively major genetic overlap is observed between prostate cancer and non-melanoma skin cancer, which share 3 predisposition regions. On top of 5p15.33 (the locus of CLPTM1L and TERT), prostate and non-melanoma skin cancer also share 6p22.1-p21.31 and 21q22.2-q22.3. The 21q22.2-q22.3 region seems to be a novel discovery of our analysis, without any existing support from the literature. The most significant variant in that region is rs2849691 (lowest GWAS *p*-value = 6E−13) that occurs in the non-coding lncRNA transcript LOC107985478. The other region, 6p22.1-p21.31, is a 4.7 Mbp genomic region covering the MHC region. Of the 138 genes in that region, 7 are significant in non-melanoma skin cancer according to PWAS: HLA-DPA1, HLA-C, PPP1R18, CCHCR1, MPIG6B, MUC22 and C6orf15 (Supplementary Table S1).

Other than the handful of shared regions mentioned here, most cancer types show little overlap with other cancers (Fig. 5). Overall, of the 145 cancer predisposition regions, only 27 are shared by multiple cancers. Disregarding pan-cancer, only 14 regions are shared by two or more specific cancer types.

### Recessive effects are prevalent in cancer predisposition

**Figure 6:**
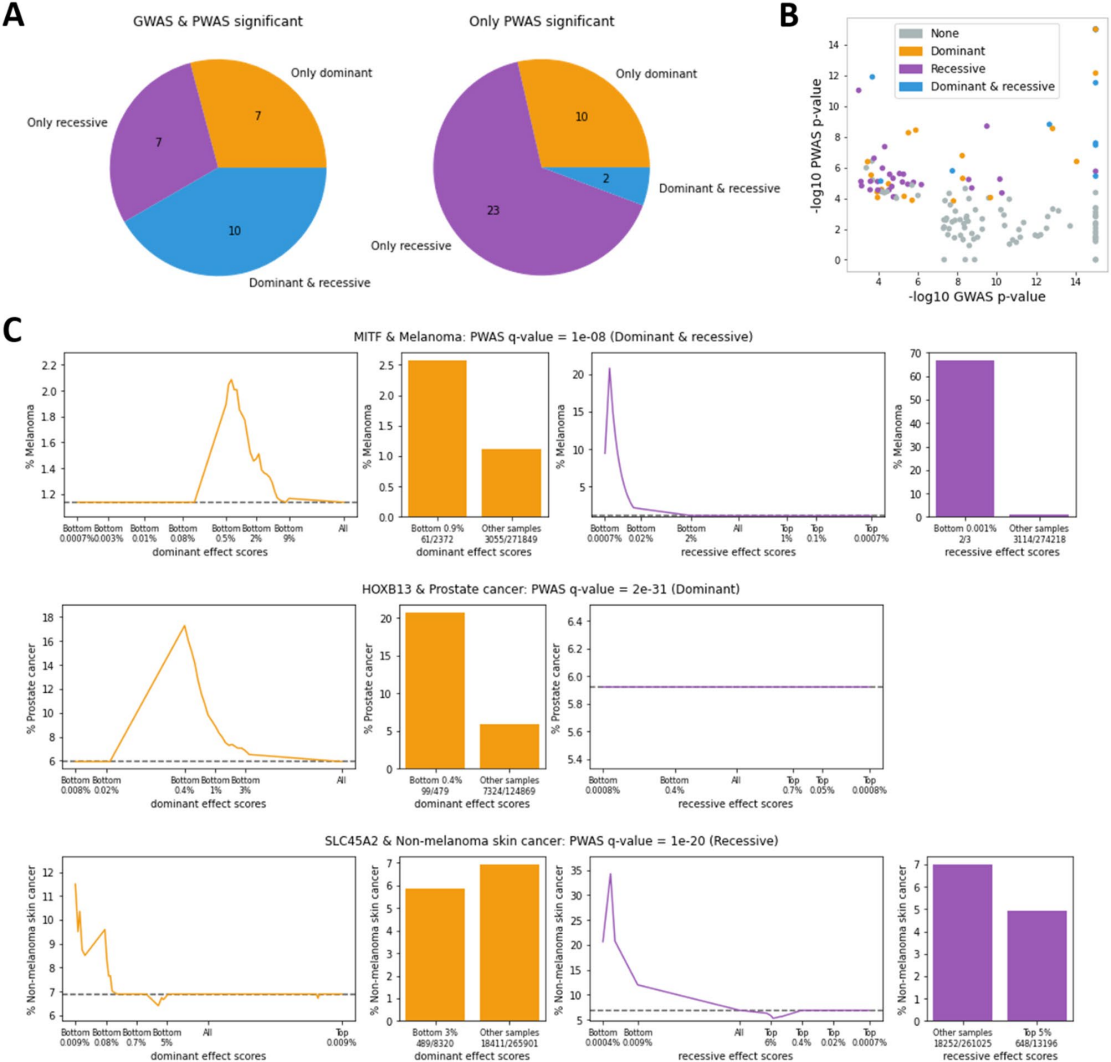
Dominant and recessive gene effects. (**A**) Partitioning of the significant cancer genomic regions that are PWAS-significant according to their inheritance mode (dominant, recessive or both), and whether they are also significant by GWAS. (**B**) GWAS and PWAS *p*-values (capped at 1E-15) across all 145 significant caner genomic regions. Regions are color coded by whether they were found significant by the dominant model of PWAS, the recessive one, both, or none. (**C**) Three examples of significant PWAS associations: MITF and melanoma, HOXB13 and prostate cancer, and SLC45A2 and non-melanoma skin cancer. The plots show how risk for the relevant cancer types changes as a function of PWAS effect scores in the UKB cohort. Dominant effect scores (orange) aim to capture the likelihood of an individual to have at least one damaged copy of the gene, whereas recessive effect scores (purple) aim to capture the likelihood of at least two damaged copies. In both types of PWAS effect scores, lower scores reflect individuals with greater gene damage. The line plots show a continuous partitioning of the cohort according to the dominant or recessive effect scores on the x-axis, and the estimated fraction of individuals with a history of the relevant cancer in each partition on the y-axis. For example, in the association between melanoma and the dominant effect scores of MITF (shown on the upper-left plot in (C)), “Bottom 0.5%” on the x-axis refers to the 0.5% of the cohort with the lowest dominant effect scores, namely the 0.5% of individuals that are the most likely to have at least one damaged copy of MITF. The prevalence of melanoma in that group is estimated at ~ 2%. The dashed horizontal line shows the baseline cancer risk across the entire cohort (1.14% cases for melanoma). The bar plots show the most significant partitioning of individuals. For example, among the 0.9% (2,372) of the individuals with the lowest dominant effect scores calculated for the MITF gene, 61 (2.57% of them) have had melanoma, compared to 1.12% rate of melanoma among the other individuals (3055 melanoma cases among 271,849 individuals). Note that the scales of the plots are not uniform. In particular, each plot shows a different partitioning of the cohort (based on the relevant distribution of effect scores).

Unlike GWAS, PWAS explicitly models dominant and recessive effects at the gene level and has the capacity to capture genetic effects that transcend the individual variant. It therefore provides the opportunity to assess the importance of these effects to cancer predisposition. We observe that recessive effects are indeed common among significant PWAS regions, and especially among the regions that are not captured by GWAS (Fig. 6A). The enrichment of recessive effects in PWAS-exclusive regions is explained by the fact that GWAS (under standard settings) models additive genetic effects. Unlike dominant effects, which can be approximated by an additive model, recessive effects cannot be effectively recovered by an additive GWAS model. The same trend shows when considering the evidence for each genomic region according to continuous *p*-values: significant regions with little evidence according to GWAS tend to be recovered by the recessive model of PWAS (Fig. 6B).

To illustrate the dominant and recessive genetic effects captured by PWAS, we selected three soliton associations with strong effects (see Table 1): MITF (melanocyte inducing transcription factor) associated with melanoma (q-value = 1E−08; both dominant and recessive effects are significant), HOXB13 (homeobox B13) associated with prostate cancer (q-value = 2E−31; only dominant effect is significant) and SLC45A2 (solute carrier family 45 member 2) associated with non-melanoma skin cancer (q-value = 1E−20; only recessive effect is significant). For each of the three associations, we plot cancer risk among UKB individuals as a function of PWAS effect scores (Fig. 6C). PWAS effect scores are assigned to each individual in the cohort with respect to each studied protein-coding gene based on their genotype. They reflect the overall estimated functional damage affecting genes at the molecular level, with lower effect scores reflecting more damage. For each individual, each coding gene is assigned two effect scores that aggregate all the variants within the gene that were genotyped or imputed for that individual: a dominant effect score (reflecting the likelihood of at least one damaging hit) and a recessive effect score (reflecting the likelihood of at least two damaging hits). These effect scores are used by PWAS to detect significant genes (if they correlate with a history of cancer), and they can also be used to assess individual-level risk (with respect to specific genes).

In the case of MITF, we observe that among the 2,372 individuals with the lowest dominant effect scores (comprising 0.9% of the cohort), 61 (2.57% of that group) have had melanoma, compared to only 1.12% risk of melanoma in the rest of the cohort (leading to observed risk-ratio of 2.3). When considering recessive effect scores, we find that 2 out of the 3 individuals with the lowest scores have had melanoma. We conclude that individuals with functional damage to the MITF protein have substantially higher rates of melanoma. As the name of the gene suggests, MITF is a transcription factor that controls the development and function of melanocytes, which are pigment-producing cells. The causal connection of MITF to melanoma is well established^59^.

Unlike MITF, showing both dominant and recessive effects (which might be interpreted as approximating an additive effect), HOXB13 shows a clear dominant effect according to PWAS. Among the 479 males with the lowest dominant effect scores (0.4% of the male cohort), 99 of them (20.7%) have a history of prostate cancer, compared to 5.87% rate of prostate cancer in the rest of the male cohort (risk-ratio of 3.5). HOXB13 regulates cellular response to androgen, an important male hormone. Its link to prostate cancer and early onset of the disease has been established in both GWAS and family studies^60^.

A clear recessive effect is exemplified by SLC45A2 (also known by the names AIM1, MATP and OCA4). Among the 13,196 individuals with highest recessive effect scores, i.e. the 5% of the UKB cohort least likely to have two damaged copies of the gene, 648 of them (4.91%) are cases of non-melanoma skin cancer, compared to 6.99% of cases in the rest of the cohort (risk-ratio of 1.4). SLC45A2 is a transporter protein likely involved in the production of melanin. The gene has indeed been implicated in melanoma and skin cancer through albinism, a disorder known for recessive inheritance^50^.

These three examples demonstrate the applicability of PWAS effect scores to clinical risk assessment. Similar plots for all 110 significant PWAS associations are available in Supplementary Fig. S2.

To further examine the recessive associations, we list the 30 genomic regions with only significant recessive effects (Fig. 6A) in Table 3 (full summary statistics for these and all other significant regions are available in Supplementary Table S1 and Supplementary Table S3). We find that 29 of the 30 recessive regions support exactly one significant association of gene and cancer-type, and we therefore identify the regions by the significant genes (the only exception is the region of SEMA3F on chromosome 3, in which another gene, MST1R, is also significant, but less so). Notably, 9 of the 30 recessive genes are enzymes (or part of enzyme complexes), which are considered a standard model for recessive effects^61^. Additional 4 genes are transporters associated with the solute carrier family (or regulators of such transporters). In fact, of all 110 significant PWAS associations, these genes are the only transporters of that family (namely, all 4 genes associated with the solute carrier family that we have recovered exhibit pure recessive effects). Both SLC12A9 and SLC45A2, which we associate with melanoma and non-melanoma skin cancer, are implicated with pigmentation according to the Open Targets Platform^62^. We also observe that many of the 30 recessive genes are involved in cellular processes of apoptosis, ubiquitination and immunology. Interestingly, leukemia seems to be particularly enriched with recessive effects. Of the 16 genomic regions associated with leukemia according to PWAS, 12 show exclusive recessive effects and are listed in Table 3. The possibility of mistaking somatic mutations as germline variation in blood cancers should be taken into account when interpreting this finding. For example, the high prevalence of recessive heritability in leukemia might be a reflection of the “second-hit” hypothesis^63^. As an external validation scheme, Table 3 also lists published cancer genetic associations curated by the Open Targets Platform. We find that the cancer types reported by the Open Targets Platform are usually in agreement with our results. Nevertheless, most recessive genes appear to represent novel genetic associations, maybe as a result of recessive genetic studies being largely neglected in case–control cohorts. When considering the external validation used earlier (Fig. 3), only 2 of the 30 recessive genes are supported by clinical panels or catalogues of cancer drivers. Specifically, MUTYH and BIRC3 are both established as cancer drivers, and they both appear in clinical panels. Overall, these 30 recessive genes highlight the importance of modeling recessive inheritance in non-familial (case–control) cancer studies.

**Table 3:** Recessive cancer predisposition genes.

Gene symbol	Gene name	Chromosome bands	Molecular function	Cancer type	Cancer genetic associations according to open targets platform
PADI1	Peptidyl Arginine Deiminase 1	1p36.13	Enzyme	Melanoma	None
AKIRIN1	Akirin 1	1p34.3-p34.2	Regulator	Lung	None
MUTYH	Adenine DNA glyc osylase	1p34.1	Enzyme	Colorectal	Colorectal and other cancers
FCGR1A	High affinity immunoglobulin gamma Fc receptor I	1q21.2	Receptor	Leukemia	None
OR6K6	Olfactory receptor 6K6	1q23.1-q23.2	Receptor	Pancreatic	None
DPT	Dermatopontin	1q24.2	ECM	Leukemia	Leukemia
TNFSF18	Tumor necrosis factor ligand superfamily member 18	1q24.3-q25.1	Cytokine	Leukemia	None
XPC	DNA repair protein complementing XP-C cells	3p25.1	DNA binding	Skin	Skin
LYZL4	Lysozyme-like protein 4	3p22.1	Enzyme	Leukemia	None
SEMA3F	Semaphorin-3F	3p21.31-p21.2	Chemotaxis	Skin	None
POLR2H	DNA-directed RNA polymerases I, II, and III subunit RPABC3	3q27.1-q27.2	Enzyme	Melanoma	None
RCHY1	RING finger and CHY zinc finger domain-containing protein 1	4q13.3-q21.1	Enzyme	Leukemia	None
PCDH10	Protocadherin-10	4q28.3	Cell adhesion	Leukemia	None
SLC45A2	Membrane-associated transporter protein	5p13.3-p13.2	Transporter	Skin	Skin and melanoma
PTPN12	Tyrosine-protein phosphatase non-receptor type 12	7q11.23-q21.11	Enzyme	Skin	None
LMTK2	Serine/threonine-protein kinase LMTK2	7q21.3-q22.1	Enzyme	Prostate	Prostate
SLC12A9	Solute carrier family 12 member 9	7q22.1	Transporter	Melanoma	Skin
STN1	CST complex subunit STN1	10q24.33-q25.1	DNA binding	Pan-cancer	Many cancers
BIRC3	Baculoviral IAP repeat-containing protein 3	11q22.1-q22.2	Regulator	Leukemia	Leukemia and other blood cancers
JDP2	Jun dimerization protein 2	14q24.3	Regulator	Skin	Leukemia
UBE3A	Ubiquitin-protein ligase E3A	15q11.2-q12	Enzyme	Pancreatic	Leukemia
SLC12A6	Solute carrier family 12 member 6	15q14	Transporter	Lung	None
STX8	Syntaxin-8	17p13.1	Receptor	Melanoma	None
ATPAF2	ATP synthase mitochondrial F1 complex assembly factor 2	17p11.2	Enzyme complex	Lung	None
AVP	Vasopressin-neurophysin 2-copeptin	20p13	Hormone	Melanoma	None
SLC2A4RG	SLC2A4 regulator	20q13.33	Regulator	Prostate	Prostate and other cancers
CCT8	T-complex protein 1 subunit theta	21q21.3	Chaperone	Leukemia	None
SGSM3	Small G protein signaling modulator 3	22q13.1-q13.2	Regulator	Leukemia	Breast
RBMX2	RNA-binding motif protein, X-linked 2	Xq25-q26.1	RNA binding	Leukemia	None
ZNF449	Zinc finger protein 449	Xq26.3	DNA binding	Leukemia	None

## Discussion

We performed a comprehensive analysis of cancer predisposition in the UKB cohort and identified 145 significant genomic regions by combining both GWAS and PWAS. The two methods complemented each other. While GWAS identifies variants with additive genetic effects, PWAS identifies coding genes with dominant or recessive effects (or a combination of these effects).

The capacity of PWAS to detect non-additive effects has proven substantial in our analysis. We detected 30 genomic regions with pure recessive effects, indicating that recessive inheritance is substantial for cancer predisposition also outside of family studies. Many of the discovered genes with significant recessive effects had not been previously implicated with cancer predisposition (Table 3), indicating that non-additive effects are neglected in contemporary genetic association studies. The main reason for the scarcity of studies on recessive effects is that GWAS and current variant-level methods are not designed to capture such genetic effects, especially when compound heterozygosity is involved. By exposing the importance of recessive inheritance to cancer predisposition and presenting a methodology for recovering it, we aim to motivate future investigation of this underexplored source of cancer risk (and of other conditions).

Many of the genomic loci recovered in this study are supported by external evidence (clinical panels and cancer drivers), while many are novel associations (Fig. 3, Table 3). While proving definite causal links is beyond the scope of this work, many of our novel discoveries appear as promising candidates. In particular, genes defined as solitons (Table 1) are likely less sensitive to linkage-disequilibrium and are therefore more likely to prove causal.

Many of the reported genes are associated with substantial cancer risk (Fig. 6C). For example, we show that individuals in the male cohort with substantial damage to HOXB13 have an increased risk of prostate cancer (20.7%, compared to 5.9% in the rest of the male cohort). We provide a similar cancer risk analysis for all 110 PWAS gene associations (Supplementary Fig. S2). The capacity for risk assessment at the resolution of whole genes is enabled by PWAS through the aggregation of all variants affecting an individual. Integration of such data into the clinics could have a substantial impact on individual and familial cancer risk evaluation. Even when variants of unknown significance are observed after the initial study, PWAS can still estimate gene damage and compare it to the population distribution, thereby providing risk assessment.

The presented analysis was conducted over nine specific cancer types and pan-cancer. We observe that most cancer predisposition loci are cancer-type specific. Only 14 of the 145 loci are shared by two or more specific cancer types, with 7 of them shared only by melanoma and non-melanoma skin cancer. Only 2 loci are shared by three or more specific cancer types (Fig. 5). The pan-cancer analysis recovered only 3 unique associations not discovered in specific cancers. This observation is in stark contrast to somatic tumor genomics, where most discovered cancer drivers are pan-cancer^36^. Our results suggest that cancer predisposition of germline genetic variation, unlike positive selection of somatic mutations in tumors, are mostly cancer-type specific. The cancer type with the highest number of recovered loci is non-melanoma skin cancer, with 58 of the 145 genomic regions associated with that cancer type. While this could be explained by the high number of cases for that cancer type, it highlights a substantial polygenic signal for non-melanoma skin cancer. Such strong polygenicity is compatible with the substantial role of environmental factors such as UV radiation and viral infection in this cancer^64^.

An important limitation of our analysis is its restriction to the white British population, as the UKB cohort does not include a substantial number of individuals from other ethnicities required for sufficient statistical power (see Methods). This leads to a lack of validation of the discovered associations on other populations, as well as oversight of additional associations only present in other ethnic groups. Another limitation of the UKB is the lack of sequencing data for the vast majority of the cohort. In this analysis we relied solely on genotyping of ~ 800 K predefined genetic markers and ~ 600 K variants in coding regions imputed from the original set of markers ^26^. This limitation is particularly relevant for PWAS, which underestimates genetic damage when non-genotyped variants are involved, leading to diminished statistical power (but, critically, not to false discoveries)^33^.

This study demonstrates the power of exhaustive genomic and protein-centric analysis over large biobanks for expanding the discovery of cancer predisposition loci beyond family studies. In particular, we have demonstrated that recessive effects are also prevalent in case–control cohorts when looked for by suitable methods. We have also demonstrated the benefit of cohorts with diverse cancer-type annotations for cross-cancer comparisons. Finally, our results show promise towards individual clinical risk assessment of cancer.

## Methods

### Study cohort

From the entire UK Biobank (UKB) cohort of 502,520 individuals (application ID 26664)^25,26^, we filtered out 227,690 individuals (see Fig. 2) for the following reasons: i) 14 individuals had asked to withdraw from the UKB, ii) 92,922 were non-whites (according to self-reported ethnicity or their genetics), iii) 312 had self-reported sex mismatching their genetics, iv) 75,848 were family-related to other individuals in the cohort (from each group of related individuals we chose only one representative), and v) 58,594 didn’t have any recorded ICD-10 codes (which were required to determine cancer history).

Following these filtrations, we remained with a study cohort of 274,830 individuals, 56,634 (20.06%) of them had a history of cancer according to their ICD-10 codes (the number of cases and controls per cancer type are listed in Table 2).

In addition to cancer status, we also extracted from the UKB the following set of covariates: sex, year of birth, 40 genetic principal components (PCs), genotyping batch (105 categories) and UKB assessment center (25 categories). Altogether, 173 covariates (including a constant intercept) were included in the GWAS and PWAS analyses. We chose using 40 PCs, which is the maximal number of PCs provided by the UKB, to minimize the risk of false discoveries due to unaccounted population structure (at the cost of slightly reduced statistical power).

While this work would clearly benefit from trans-ethnic analysis (in particular, validation of the reported associations on independent cohorts of other ethnicities)^65^, the UKB does not include a sufficient number of samples from any non-white ethnic group to support such analysis. The largest minority group in the UKB is the Indian group which includes ~ 6000 samples. Such sample size is sufficient in the presence of continuous traits or very common binary traits, but not binary traits with a prevalence of 1–2% (which is the typical prevalence of most cancer types; see Table 2). Specifically, a sample size of ~ 6000 and a disease prevalence smaller than 2% entails only up to ~ 100 cases. In other words, under such settings, the number of events would be smaller than the number of variables (having 173 covariates), while PLINK recommends at least 10 events per variable^66^.

The computational pipeline for processing the UK Biobank data used in this work is an open-source project available at https://github.com/nadavbra/ukbb_parser.

### GWAS

To run GWAS, we used the PLINK software^66^ (https://www.cog-genomics.org/plink/2.0/; version v2.00a2.3LM 64-bit Intel, 24 Jan 2020). For example, to run GWAS for breast cancer over chromosome 1, we ran the following command:

*plink2 --glm --bed ukb_cal_chr1_v2.bed --bim ukb_snp_chr1_v2.bim --fam ukb2666_cal_chr1_v2_s488366.fam --pheno breast_cancer.txt --covar covariates.txt --out chr1_breast_cancer --1 hide-covar no-x-sex --mac 20 --covar-variance-standardize --freq --threads 32 --memory 100000.*

Where *ukb_cal_chr1_v2.bed*, *ukb_snp_chr1_v2.bim* and *ukb2666_cal_chr1_v2_s488366.fam* are the files with the genetic data of chr1, provided by the UKB. The files *breast_cancer.txt* and *covariates.txt* are PLINK-formatted tab-separated data files with the cancer phenotype (case–control status) and covariates of each of the samples in the study cohort, respectively. The *--glm* argument specifies the use of a linear/logistic model; the *--1* flag specifies that cases and controls are encoded as 1 and 0, respectively; the *hide-covar* flag indicates that summary statistics are to be reported only with respect to the phenotype (but not the individual covariates); the no-x-sex flag indicates that sex is not to be re-added as a covariate (since it’s already included among the provided covariates); *--mac 20* limits the analysis to variants with at least 20 occurrences of the minor allele (according to PLINK’s recommendation), so that the linear model is properly calibrated; *--covar-variance-standardize* linearly transforms all quantitative covariates to mean-zero, variance 1; *–freq* specifies that allele frequencies are to be calculated and reported; and *--threads 32 --memory 10,000* allocate 32 CPU cores and ~ 10 GB of RAM for the process.

We ran GWAS independently over ten cancer phenotypes (nine specific cancer types and pan-cancer) and over 26 chromosomes (22 autosomal chromosomes, chrX, chrY, chrXY and chrMY, as provided by the UKB). The complete GWAS results with all the summary statistics, including 883 variant-cancer associations below the exome-wide significance threshold (5E−07), are available per cancer-type in Supplementary Table S2.

### PWAS

Proteome-Wide Association Study (PWAS) is a protein-centric, gene-based method for genetic association studies (Fig. 1). The method is fully described and validated in a previous work^33^. In the case of cancer predisposition, PWAS detects protein-coding genes that are significantly more (or less) damaged in individuals with a history of cancer. To determine to what extent a given gene is damaged within a given individual (based on his or her genetics), PWAS employs a machine-learning model that assigns functional effect score to each variant affecting the coding sequence of the gene. Specifically, PWAS uses the FIRM predictor, a random-forest classifier that assigns each variant a score reflecting the probability of the gene to retain its function in the presence of that variant^36^. FIRM relies on 1109 numerical features capturing the rich proteomic context of the gene and the affecting variant. The main features included in FIRM are: (i) the location of the variant within the protein sequence, (ii) the reference and alternative amino acids, (iii) amino-acid scales (i.e. various numeric values assigned to amino acids, in different contexts of the protein and the variant); (iv) protein annotations extracted from UniProt^67^ (e.g. secondary structure, post-translational modifications, functional sites, etc.), and (v) Pfam domain and clan annotations^68^ (e.g. whether the variant is within or next to a domain). Following the assignment of effect scores to individual variants, PWAS aggregates the effect scores of all variants in the coding sequence of the gene (in the context of the individual’s genetics). Specifically, it calculates dominant and recessive effect scores which reflect the probabilities for at least one or at least two damaging events in the gene, respectively. Finally, PWAS tests whether the dominant and recessive effect scores of the gene are significantly different between cases and controls. Under the combined model (for which *p*-values are reported in this work), the tested null hypothesis is that the coefficients of both the dominant and recessive effect scores are zero when fitting a logistic regression model to explain cancer status. In other words, a significant PWAS association indicates that at least one of the two effect scores (or some linear combination of them) is associated with cancer predisposition.

We used version 1.0.4 of the PWAS software (available at https://github.com/‌nadavbra‌/‌pwas) and executed the standard PWAS pipeline (as specified on that GitHub page). We derived the dominant and recessive PWAS effect scores for the entire UKB cohort (which depend solely on the genetic information) and tested associations between 18,053 protein-coding genes and the ten cancer phenotypes. The complete PWAS results with full summary statistics, which include 110 FDR-significant gene-cancer associations, are available per cancer type in Supplementary Table S3.

### Multiple testing correction

We accounted for multiple testing by using the accepted exome-wide significance threshold ($p<5{E}^{-7}$) in GWAS, and false discovery rate (FDR) with the standard significance threshold ($q<0.05$) across all 18,053 tested genes in PWAS. Nonetheless, some interpretations of our results may warrant stricter significance threshold. First, the genome-wide significance threshold (5E−08) is more appropriate when considering non-exonic regions by GWAS. Second, as our analysis considers ten different cancer types, another factor of 10 in the significance threshold might be justified (e.g. 0.005 instead of 0.05 in PWAS). On the other hand, if one considers only a specific cancer type of interest, then this would not be unnecessary. A conservative interpretation of our results may warrant further division of the significance threshold by another factor of 2, to account for the fact that both GWAS and PWAS have been used (even though this might be somewhat too conservative, as the two methods are applied over the same data and are not completely independent). As always, *p*- and q-values are best interpreted as continuous measures of statistical evidence rather than dichotomous markers of significance. A prudent interpretation of statistical analysis should always consider their exact values, and not only whether or not they are below a certain threshold. Due to the considerations outlined here, GWAS results with *p*-values between 2.5E−09 and 5E−07 and PWAS results with q-values between 0.0025 and 0.05 may be seen as borderline. To allow easy interpretation of our results and comparison between GWAS and PWAS in exonic regions, we decided to stick with the simple thresholds of $p<5{E}^{-7}$ and $q<0.05$ throughout this work, while making available the full summary statistics across all regions (Supplementary Table S1), variants (Supplementary Table S2) and genes (Supplementary Table S3).

### Merging the significant associations into unique genomic regions

We merged the 883 significant GWAS associations and 110 significant PWAS associations into 145 unique genomic regions by extending each association by 500,000 base-pairs on both sides and iteratively merging overlapping regions. GWAS associations started as 1 bp and were extended into 1,000,001 bp regions (the position of the variant, plus 500,000 bp on each side) while PWAS associations started as the region of the gene’s coding-sequence (i.e. from the genomic coordinate of the start codon to the last before the stop codon). This merging procedure resulted in 145 non-overlapping genomic loci of sizes of at least 1Mbp, available in Supplementary Table S1. The largest obtained locus was chr6:30,145,049–34,892,998 (of size 4.7 Mbp), which covers the MHC region. This region contains 7049 variants genotyped by the UKB (223 of which are GWAS-significant) and 138 protein-coding genes tested by PWAS (7 of them are PWAS-significant).

### External cancer evidence

We validated each of the 145 cancer predisposition regions against three catalogues of cancer drivers (Census^34^, pan-software^35^ and FABRIC^36,37^) and two gene panels used in the clinics (CleanPlex TMB 500 and Invitae Multi-Cancer). The “Cancer Gene Census” was downloaded as a CSV file (cancer_gene_census.csv, from https://cancer.sanger.ac.uk/cosmic/download) containing 723 genes. Each of the Census genes is identified as a tumor-suppressor gene (TSG), oncogene or a fusion gene (or any combination of these three categories). The list of 515 genes in the CleanPlex TMB 500 panel was downloaded from https://www.paragongenomics.com/wp-content/uploads/2019/04/PS1008_CleanPlex_TMB-500-Panel_Gene-List.txt, and the list of 83 genes in the Invitae Multi-Cancer panel was downloaded from https://www.invitae.com/en/​physician/tests/01101/#info-panel-assay_information. The list of 83 Invitae Multi-Cancer genes was further extended by 50 extra genes, contributed by Vardiela Meiner from the Center for Clinical Genetics in Hadassah Medical Center (Supplementary Table S4). The Census gene list was downloaded in May 2020, and the two panel gene lists were downloaded in June 2020. The list of 299 pan-software driver genes was downloaded from Supplementary Table S1 of the publication^35^. The list of 593 FABRIC driver genes was downloaded from Supplementary Table S1 of the publication ^36^. We mapped the obtained lists of genes onto our 18,053 analyzed genes according to their UniProt ID (in the case of FABRIC) or gene symbols (in the case of the other resources). For each of the 145 genomic regions listed in Supplementary Table S1, we include all the genes in the region that are supported by each of these external catalogues.

In Fig. 3, we consider a genomic locus to be supported by clinical panels or driver catalogues if it contains at least one gene listed in those external resources. In Table 1 and Table 3, we consider the external evidence to be relevant only if it matches the exact gene listed on the table.

In Table 3 we also include cancer associations of the listed genes according to the Open Targets Platform^62^. We consider a reported association to be relevant only if (i) it is related to cancer or neoplasm, and (ii) it is supported by genetic association (as opposed to the overall association score reported by the platform, which also includes other sources of evidence, including somatic mutations).

### Determining whether PWAS-estimated gene damage is associated with elevated or reduced cancer risk

In Table 1 we indicate for each of the genes recovered by PWAS whether estimated protein damage is associated with cancer risk or protection, based on the signs the Cohen’s d summary statistics reported by PWAS. Cohen’s d is the (normalized) difference in mean gene effect score between cases and controls (calculated independently for both dominant and recessive effect scores). When negative, it indicates that cases have a lower mean effect score (i.e. more gene damage) compared to controls, meaning that gene damage is associated with elevated cancer risk. Conversely, a positive Cohen’s d indicates that gene damage is associated with reduced risk.

### Cancer risk as a function of PWAS effect scores

In Fig. 6C and Supplementary Fig. S2 we illustrate the potential clinical utility of PWAS associations by plotting the risk for the relevant cancer types as a function of dominant and recessive effect scores (which capture the overall amount of gene damage to the relevant gene, per individual). The underlying data for these figures (cancer case–control status and PWAS effect scores) was taken directly from the study cohort (see the relevant sections in the Methods). In the continuous line plots, we examine the entire distribution of PWAS effect scores by considering all possible cutoffs of the relevant cohort, i.e. all subsets with effect scores that are either below or above a certain threshold. Since the precise effect-score values are not very informative, we describe the relevant sub-populations by percentiles (e.g. “bottom 0.9%”, referring to the 0.9% of the cohort with the lowest scores, namely the 0.9% with the greatest estimated functional damage to the gene, or “top 5%”, referring to the 5% of the cohort with the highest scores, namely the 5% with the least estimated functional damage to the gene). Each of the continuous plots also includes a reference to the entire cohort, marked on the x-axis as “All”. Cutoffs of bottom percentiles are shown to the left of the entire-cohort reference point, and cutoffs of top percentiles are shown to its right (both in log scale). On the y-axis of these plots we show the estimated prevalence of the relevant cancer type in the study cohort. Note that the estimated cancer prevalence is not the naïve fraction of individuals who were diagnosed with the relevant cancer type (among the group of individuals described by the relevant partition). Rather, it is based on the 95% confidence interval for the fraction of cases in the relevant group, calculated by Wilson score interval’s method for binomial proportion. When the confidence interval overlaps with the baseline cancer rate (the dashed horizontal line in the plot), then the estimated prevalence is taken to be that baseline value. When the entire confidence interval is strictly above or below the baseline rate, then the more conservative bound of the confidence interval (that is closer to the baseline rate) is taken as the estimated prevalence. In other words, when the confidence interval is above the baseline rate, then its lower bound is taken, and when it is below the baseline rate, then its higher bound is taken. By relying on the 95% confidence interval, rather than naïve frequency of cases, more reliable estimates are obtained when dealing with small groups at the tails of the distributions (which could show wild random variations due to small numbers).

In addition to the continuous plots that capture the entire distribution of PWAS effect scores, we also illustrate the associations by choosing specific cutoffs showing the strongest differences. Specifically, we tested each cutoff by Fisher’s exact test for the association between the effect-score cutoff to cancer case–control status, selecting the cutoff with the lowest *p*-value (and display the plot only if it’s lower than 1E−03).

Note that these plots do not account for covariates, and should therefore not be considered as rigorous statistical evidence for the associations (unlike the summary *p*-values reported by PWAS, which account for covariates and consider the entire distribution of effect scores).

## Supplementary Information

Supplementary Figures.

Supplementary Table S1.

Supplementary Table S2.

Supplementary Table S3.

Supplementary Table S4.
