# Supplementary material for: Genetic association studies of alterations in protein function expose recessive effects on cancer predisposition
Source: Sci Rep. 2021 Jul 21;11:14901. doi: 10.1038/s41598-021-94252-y (PMC8295298; doi:10.1038/s41598-021-94252-y)

Supplementary Figures

Genetic association studies of alterations in protein function expose recessive effects on cancer predisposition

Nadav Brandes, Nathan Linial, Michal Linial

Contents:

Supplementary Fig. S1: Genome-wide cancer predisposition in all ten cancer-types (an extension of Fig. 4 in the main text; page 2).

Supplementary Fig. S2: Cancer risk across all 110 PWAS-significant genes (an extension of Fig. 6C in the main text; page 6).

Supplementary Fig. S1: Genome-wide cancer predisposition in all ten cancer-types

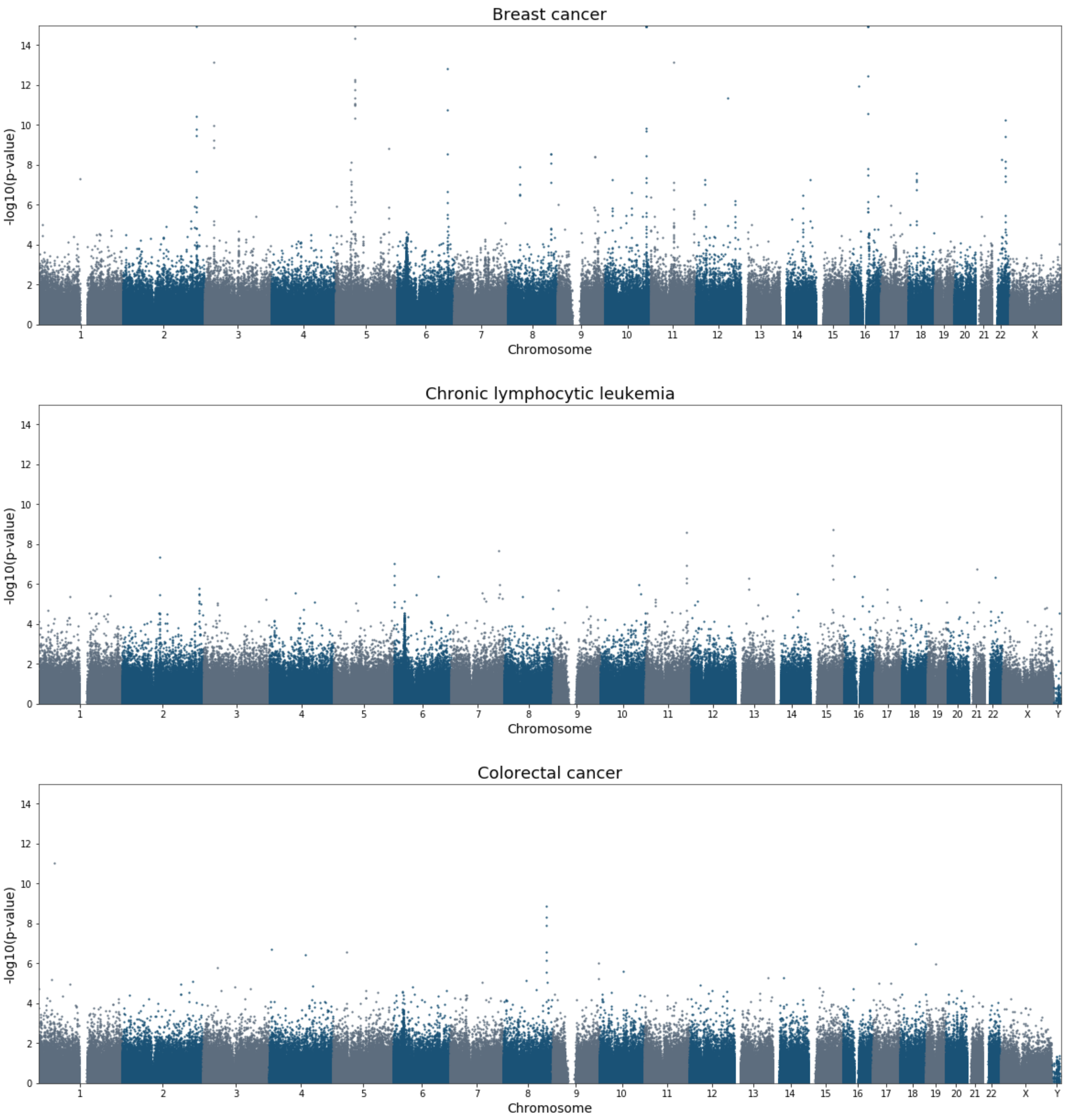

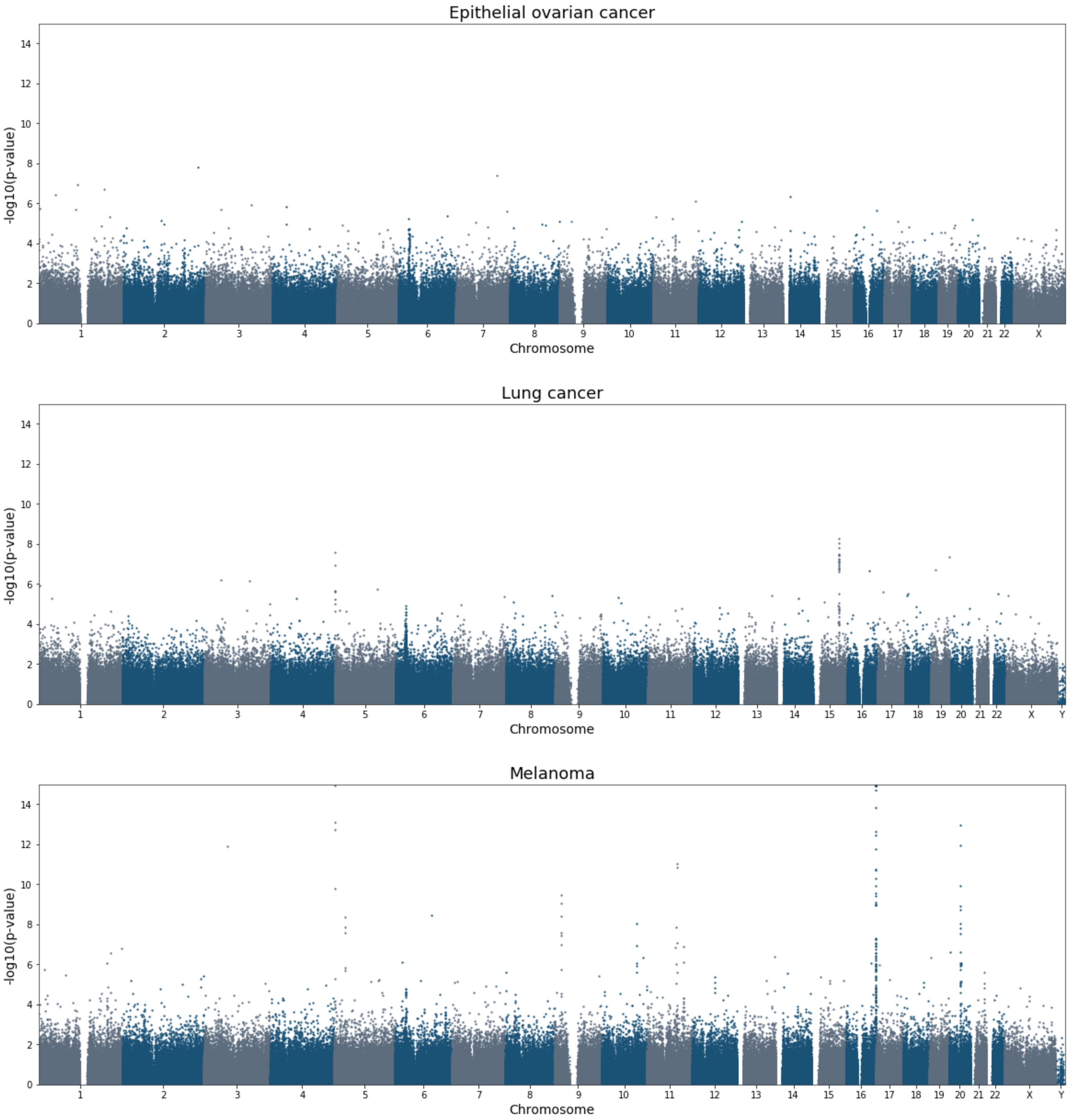
Epithelial ovarian cancer
-log10(p-value)
0
2
4
6
8
10
12
14
1
2
3
4
5
6
7
8
9
10
11
12
13
14
15
16
17
18
19
20
21
22
X
Chromosome
Lung cancer
-log10(p-value)
0
2
4
6
8
10
12
14
1
2
3
4
5
6
7
8
9
10
11
12
13
14
15
16
17
18
19
20
21
22
X
Y
Chromosome
Melanoma
-log10(p-value)
0
2
4
6
8
10
12
14
1
2
3
4
5
6
7
8
9
10
11
12
13
14
15
16
17
18
19
20
21
22
X
Y
Chromosome

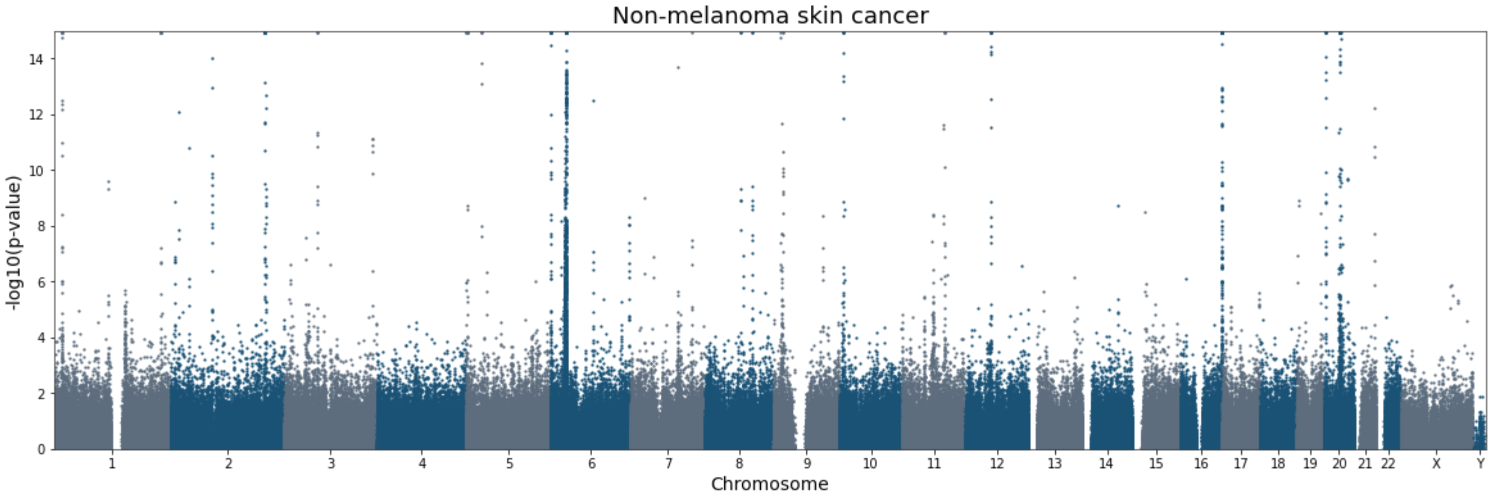
Non-melanoma skin cancer
-log10(p-value)
0 2 4 6 8 10 12 14
Chromosome
1 2 3 4 5 6 7 8 9 10 11 12 13 14 15 16 17 18 19 20 21 22 X Y

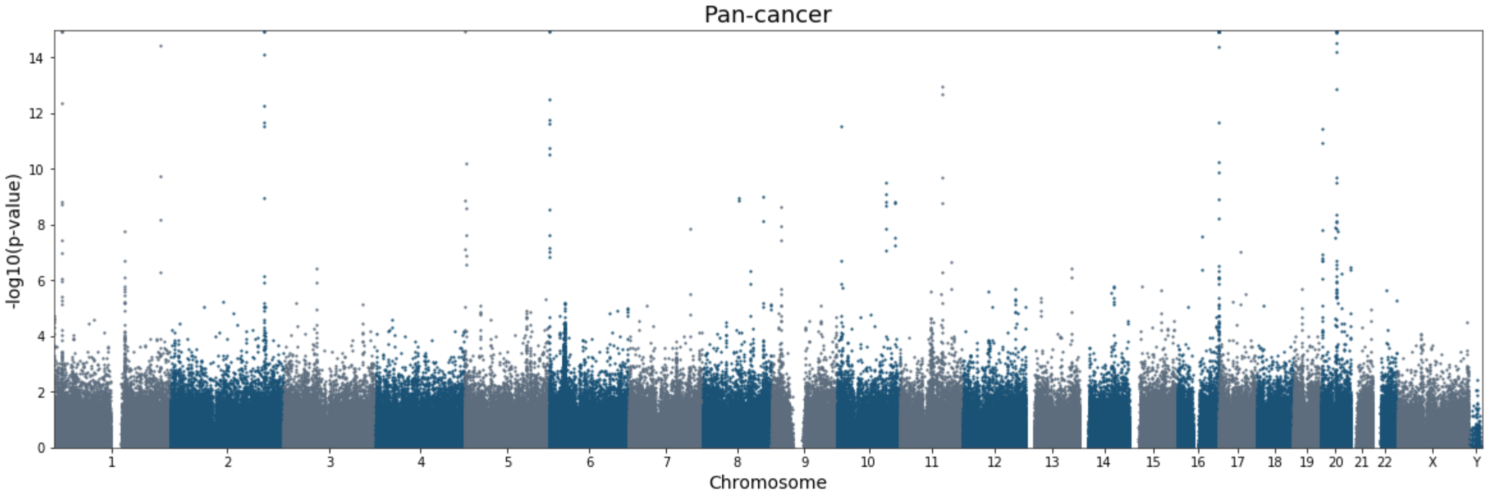
Pan-cancer
-log10(p-value)
0 2 4 6 8 10 12 14
Chromosome
1 2 3 4 5 6 7 8 9 10 11 12 13 14 15 16 17 18 19 20 21 22 X Y

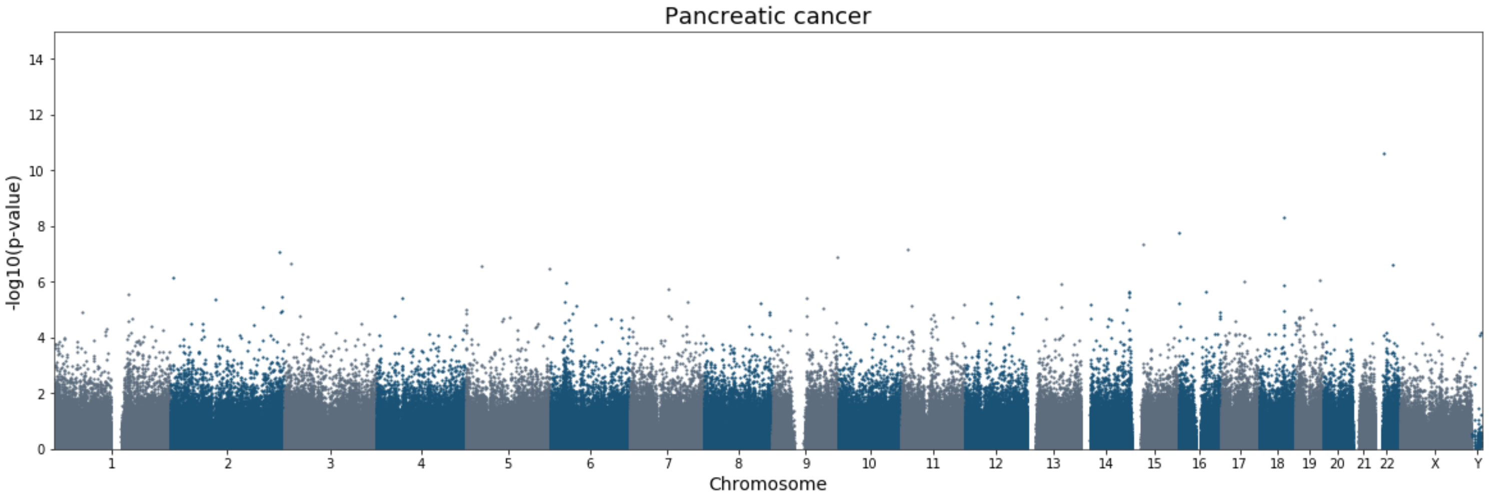
Pancreatic cancer
-log10(p-value)
0 2 4 6 8 10 12 14
Chromosome
1 2 3 4 5 6 7 8 9 10 11 12 13 14 15 16 17 18 19 20 21 22 X Y

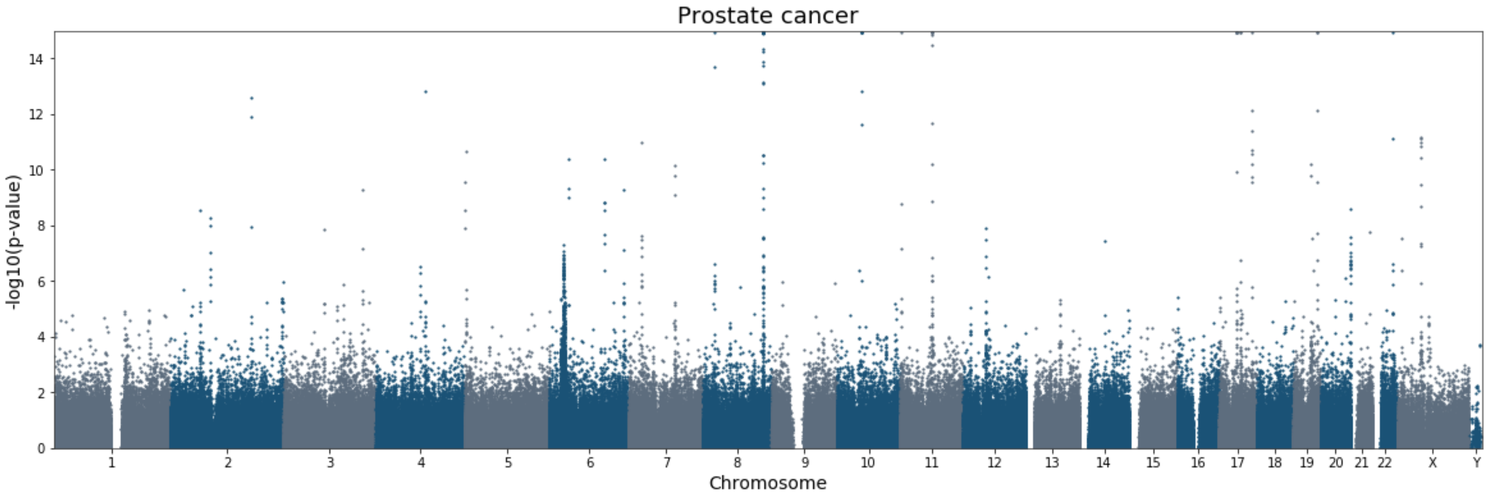

Prostate cancer
-log10(p-value)
Chromosome
0
2
4
6
8
10
12
14
1
2
3
4
5
6
7
8
9
10
11
12
13
14
15
16
17
18
19
20
21
22
X
Y

Supplementary Fig. S2: Cancer risk across all 110 PWAS-significant genes

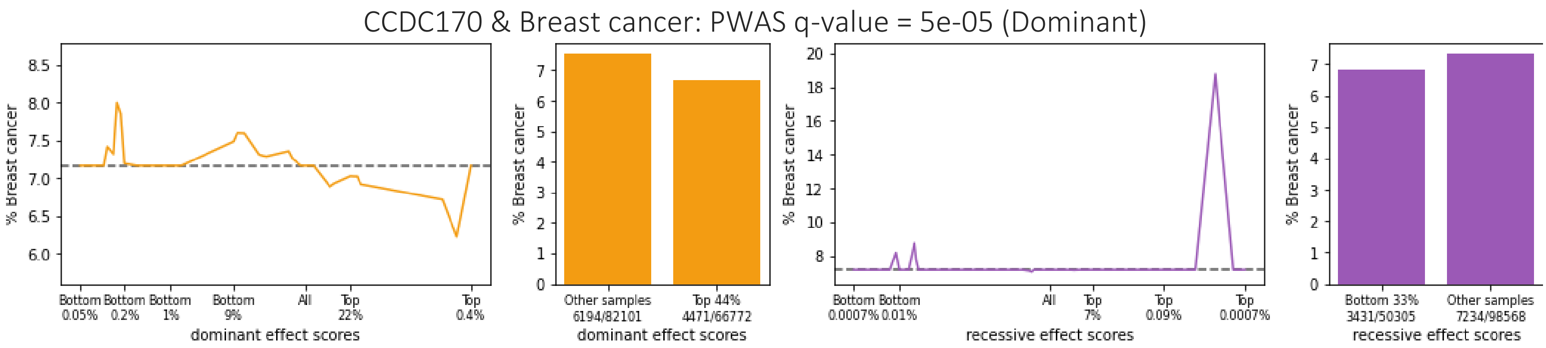

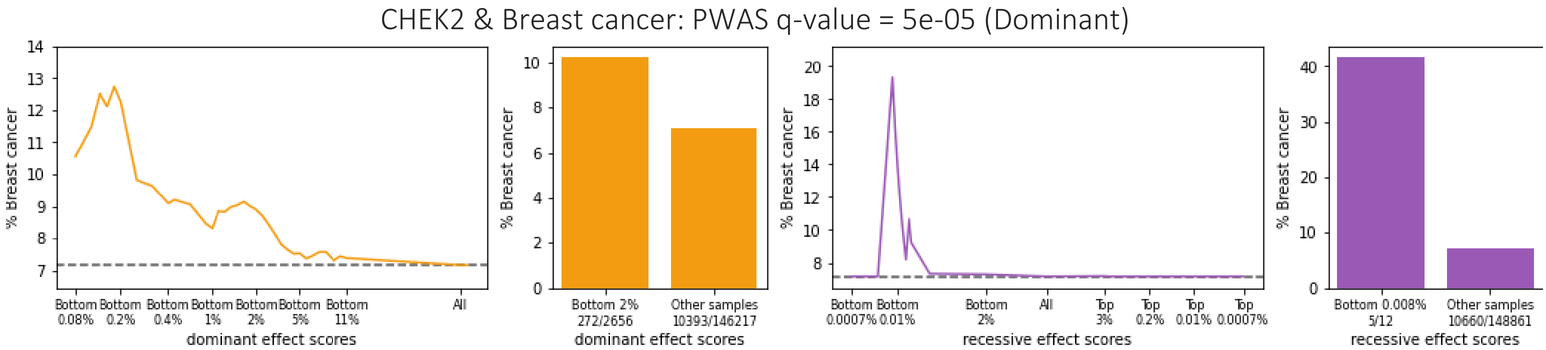

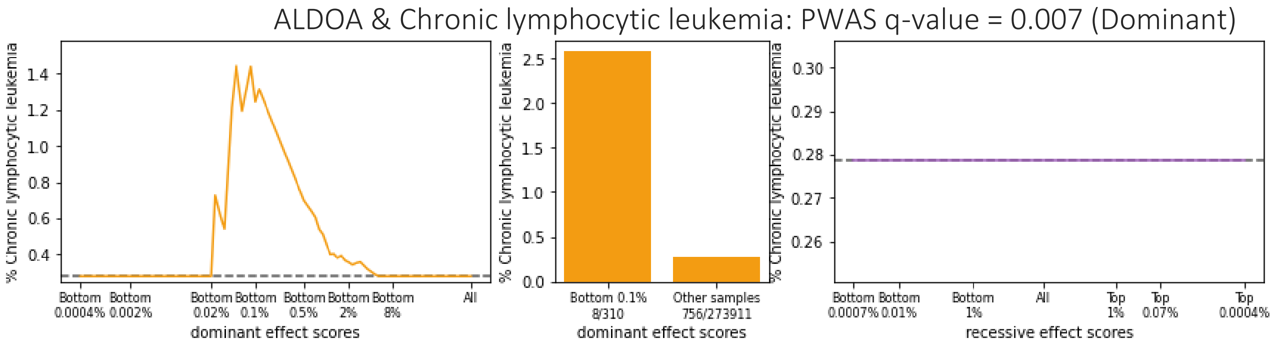

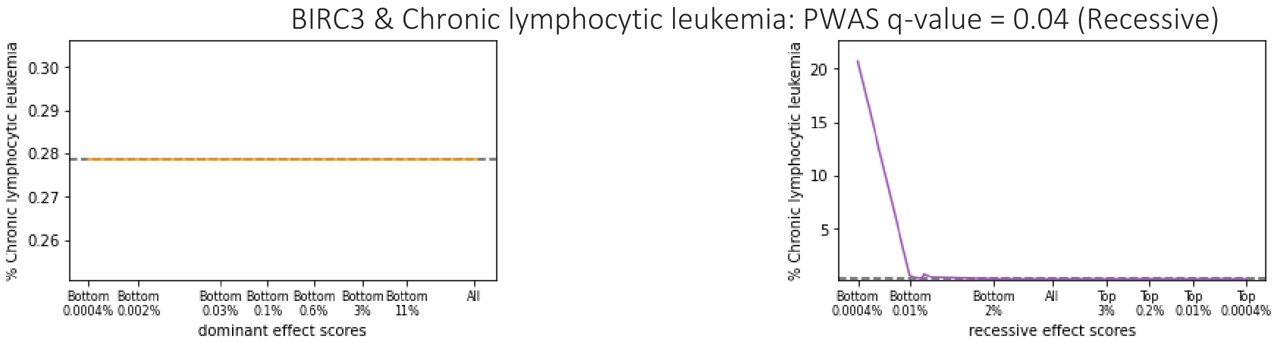

CCT8 & Chronic lymphocytic leukemia: PWAS q-value = 0.02 (Recessive)

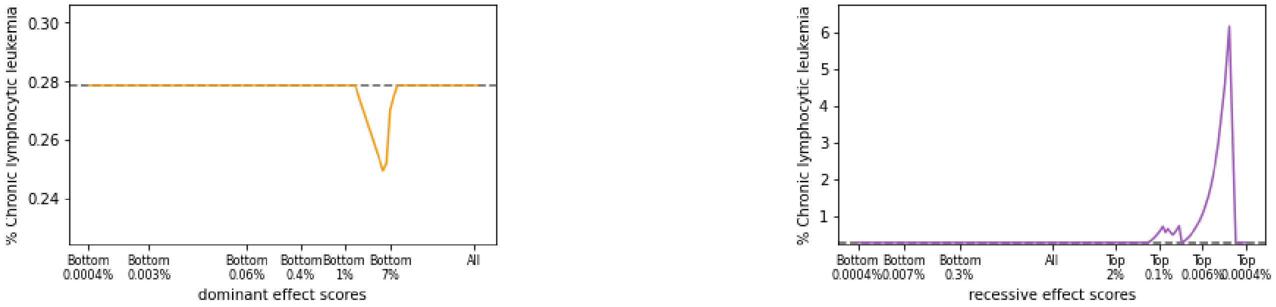

CTNNAL1 & Chronic lymphocytic leukemia: PWAS q-value = 0.05 (Dominant)

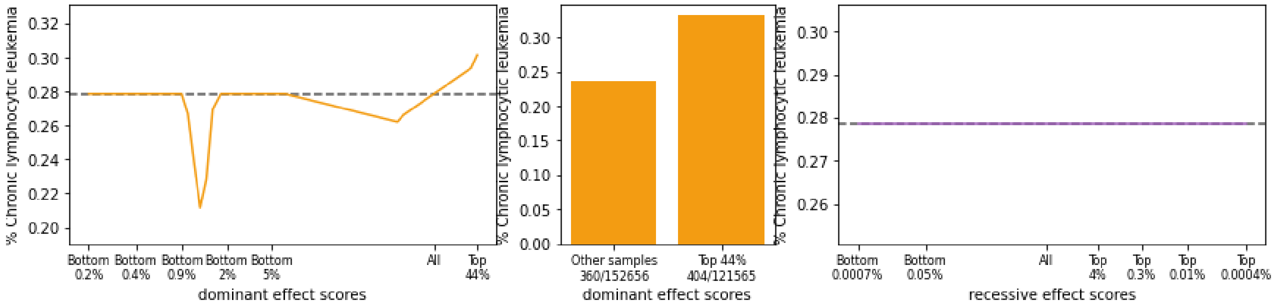

DPT & Chronic lymphocytic leukemia: PWAS q-value = 0.04 (Recessive)

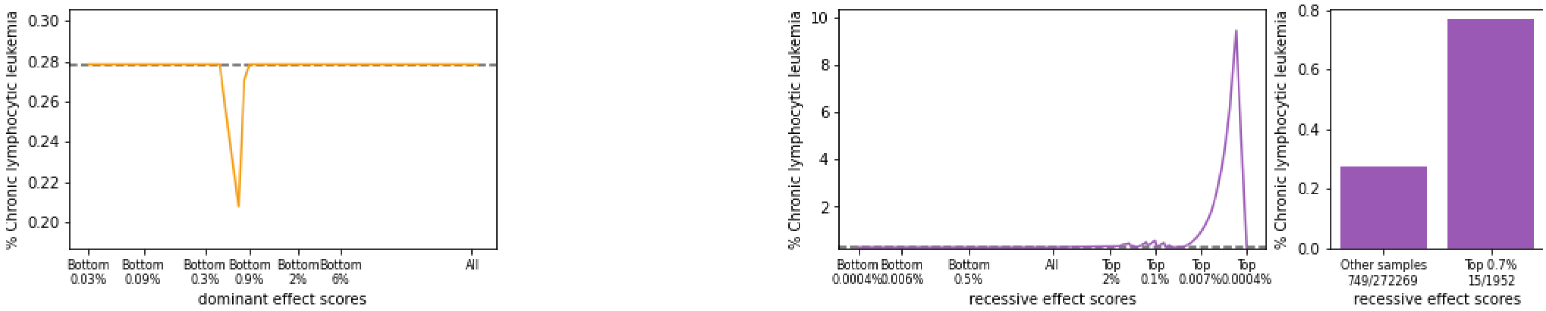

FAM160B1 & Chronic lymphocytic leukemia: PWAS q-value = 0.009 (None)

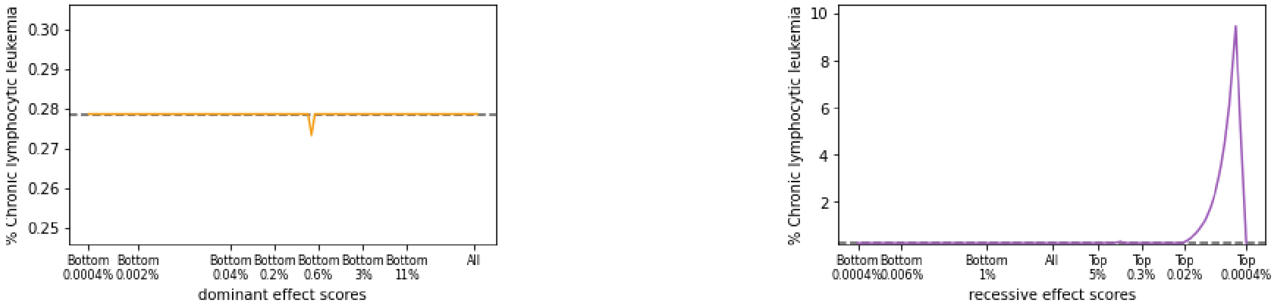

FCGR1A & Chronic lymphocytic leukemia: PWAS q-value = 0.04 (Recessive)

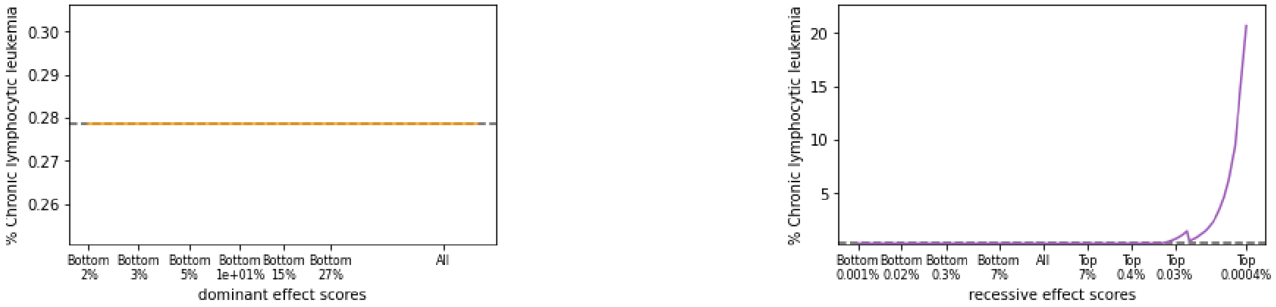

LYZL4 & Chronic lymphocytic leukemia: PWAS q-value = 0.02 (Recessive)

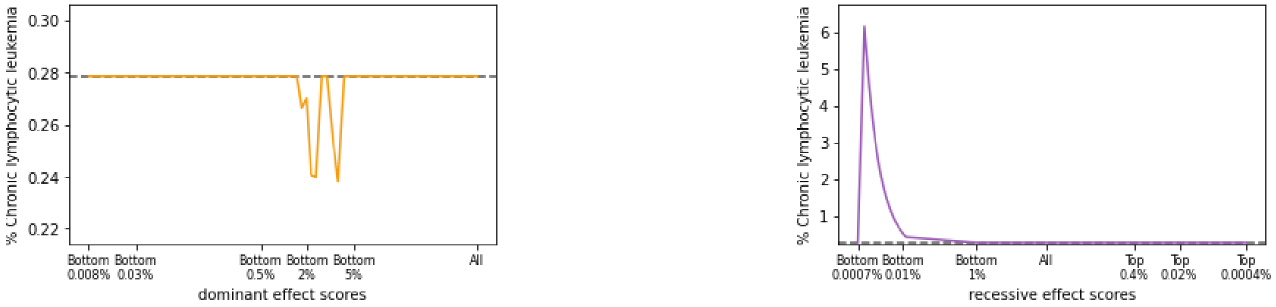

NLRP9 & Chronic lymphocytic leukemia: PWAS q-value = 0.02 (Recessive)

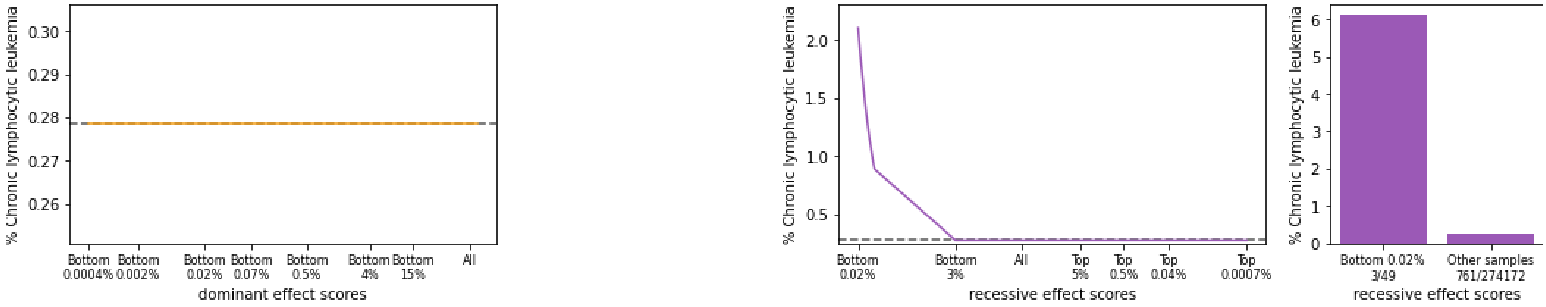

PCDH10 & Chronic lymphocytic leukemia: PWAS q-value = 0.02 (Recessive)

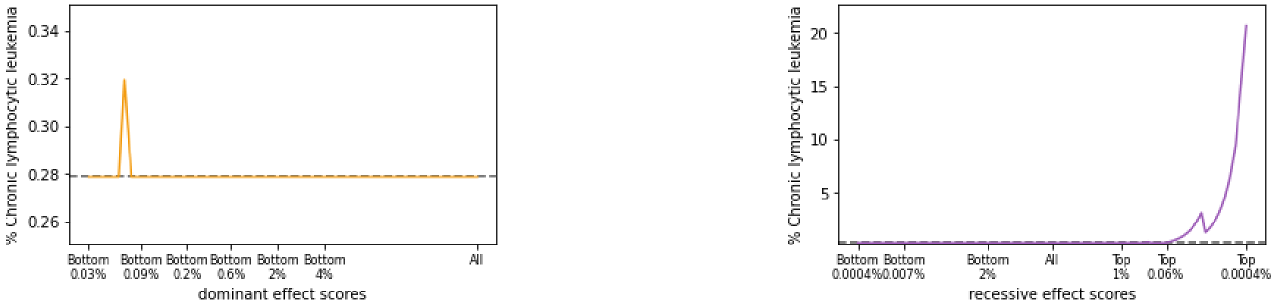

RBMX2 & Chronic lymphocytic leukemia: PWAS q-value = 0.03 (Recessive)

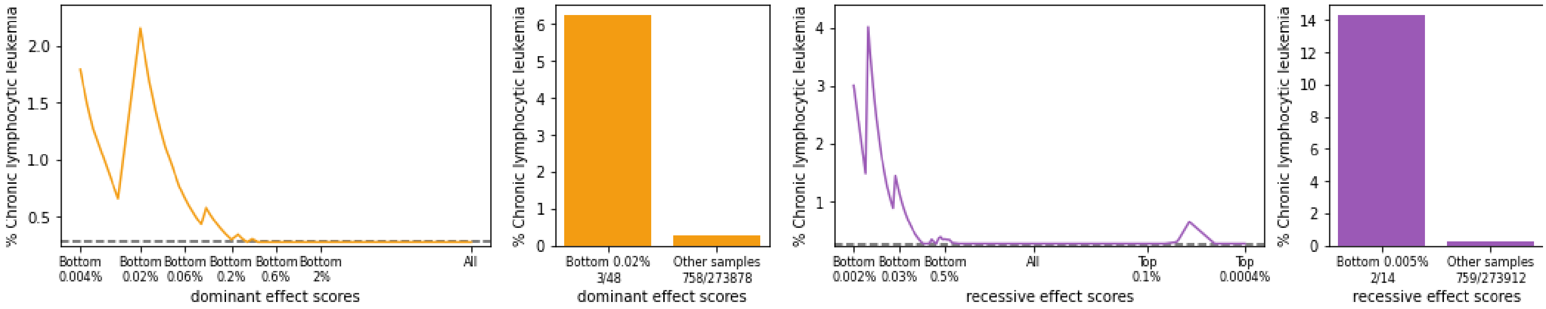

RCHY1 & Chronic lymphocytic leukemia: PWAS q-value = 0.01 (Recessive)

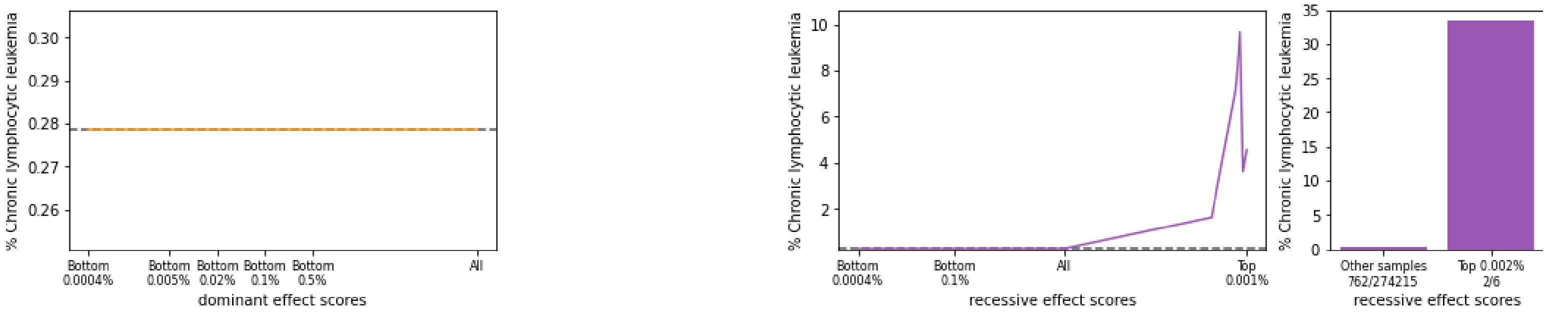

SGSM3 & Chronic lymphocytic leukemia: PWAS q-value = 0.05 (Recessive)

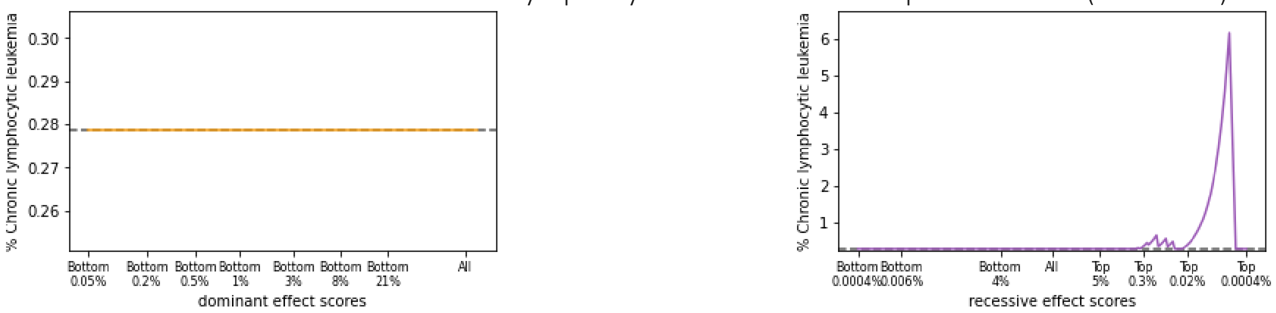

SRSF5 & Chronic lymphocytic leukemia: PWAS q-value = 0.01 (Dominant)

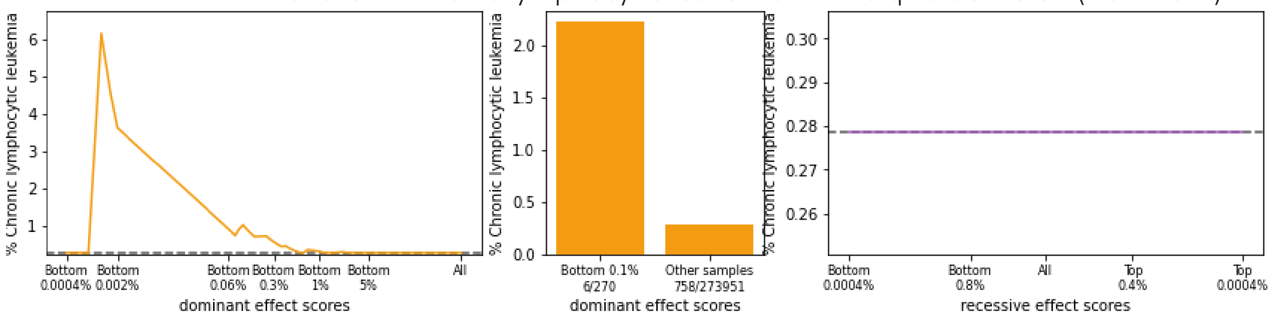

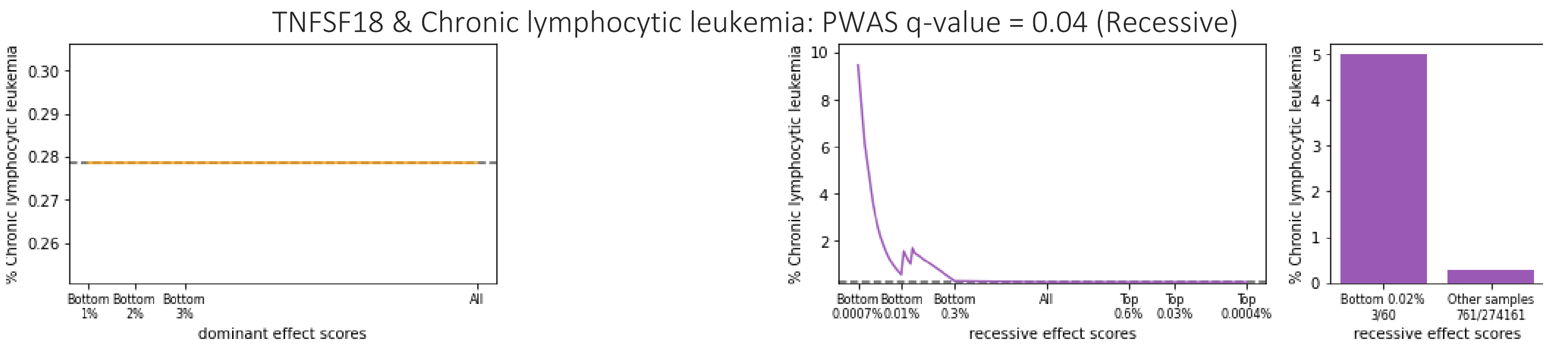
TNFSF18 & Chronic lymphocytic leukemia: PWAS q-value = 0.04 (Recessive)
% Chronic lymphocytic leukemia
0.30
0.29
0.28
0.27
0.26
Bottom 1%
Bottom 2%
Bottom 3%
All
dominant effect scores
10
8
6
4
2
Bottom 0.0007%
Bottom 0.01%
Bottom 0.3%
All
Top 0.6%
Top 0.03%
Top 0.0004%
recessive effect scores
5
4
3
2
1
0
Bottom 0.02% 3/60
Other samples 761/274161
recessive effect scores

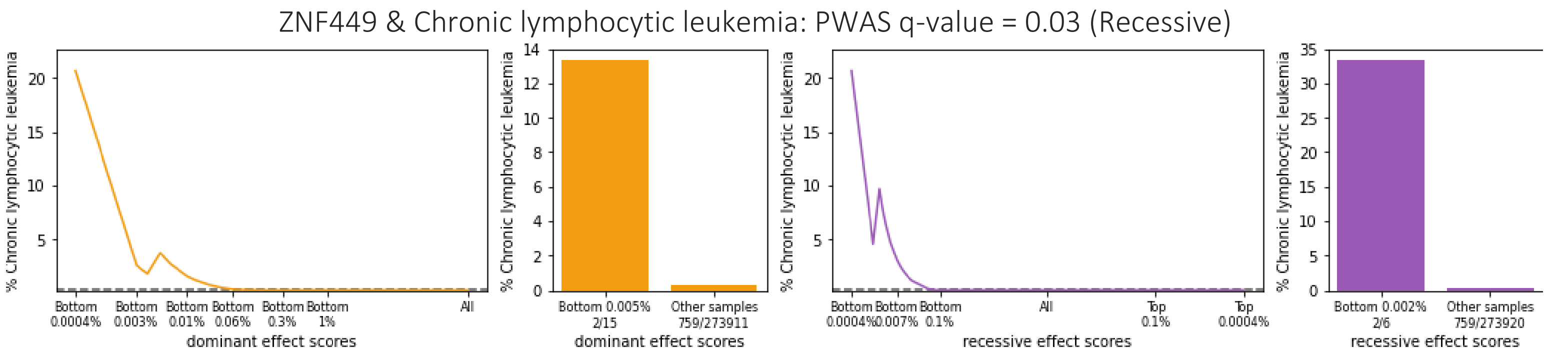
ZNF449 & Chronic lymphocytic leukemia: PWAS q-value = 0.03 (Recessive)
% Chronic lymphocytic leukemia
20
15
10
5
Bottom 0.0004%
Bottom 0.003%
Bottom 0.01%
Bottom 0.06%
Bottom 0.3%
Bottom 1%
All
dominant effect scores
14
12
10
8
6
4
2
0
Bottom 0.005% 2/15
Other samples 759/273911
dominant effect scores
Bottom 0.0004%
Bottom 0.007%
Bottom 0.1%
All
Top 0.1%
Top 0.0004%
recessive effect scores
35
30
25
20
15
10
5
0
Bottom 0.002% 2/6
Other samples 759/273920
recessive effect scores

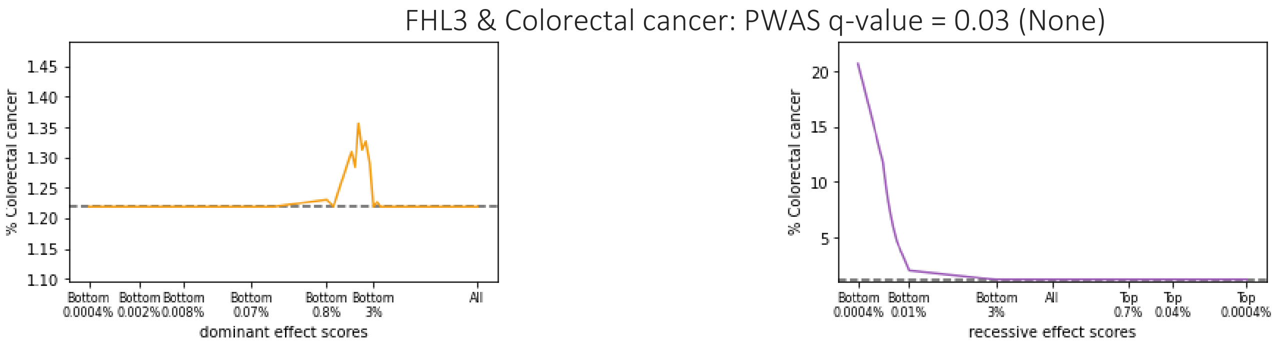
FHL3 & Colorectal cancer: PWAS q-value = 0.03 (None)
% Colorectal cancer
1.45
1.40
1.35
1.30
1.25
1.20
1.15
1.10
Bottom 0.0004%
Bottom 0.002%
Bottom 0.008%
Bottom 0.07%
Bottom 0.8%
Bottom 3%
All
dominant effect scores
20
15
10
5
Bottom 0.0004%
Bottom 0.01%
Bottom 3%
All
Top 0.7%
Top 0.04%
Top 0.0004%
recessive effect scores

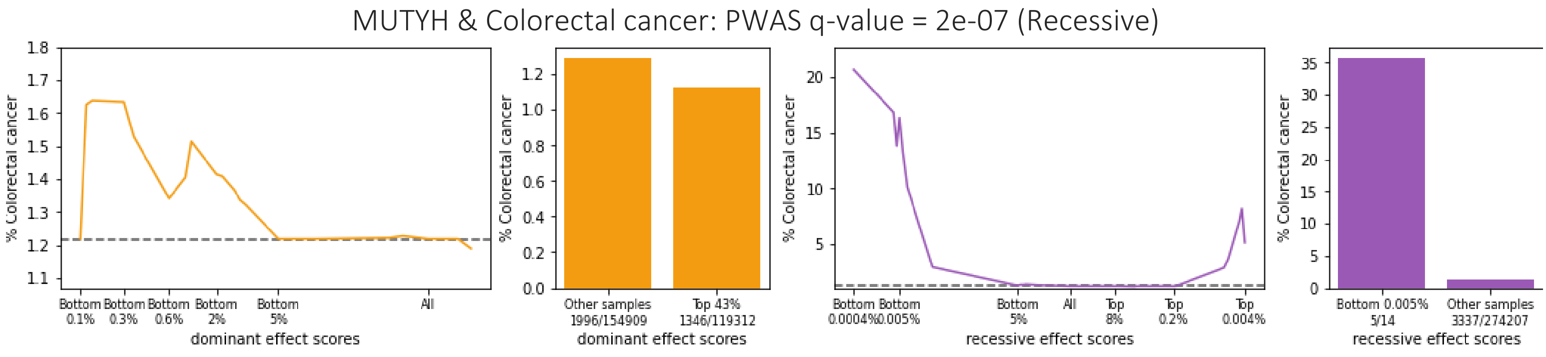
MUTYH & Colorectal cancer: PWAS q-value = 2e-07 (Recessive)
% Colorectal cancer
1.8
1.7
1.6
1.5
1.4
1.3
1.2
1.1
Bottom 0.1%
Bottom 0.3%
Bottom 0.6%
Bottom 2%
Bottom 5%
All
dominant effect scores
1.2
1.0
0.8
0.6
0.4
0.2
0.0
Other samples 1996/154909
Top 43% 1346/119312
dominant effect scores
20
15
10
5
Bottom 0.0004%
Bottom 0.005%
Bottom 5%
All
Top 8%
Top 0.2%
Top 0.004%
recessive effect scores
35
30
25
20
15
10
5
0
Bottom 0.005% 5/14
Other samples 3337/274207
recessive effect scores

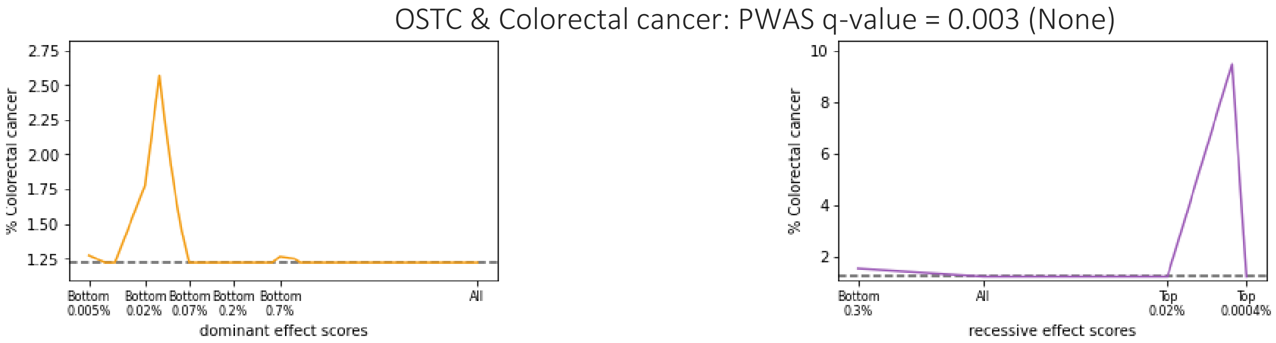
OSTC & Colorectal cancer: PWAS q-value = 0.003 (None)
% Colorectal cancer
Bottom 0.005%
Bottom 0.02%
Bottom 0.07%
Bottom 0.2%
Bottom 0.7%
All
dominant effect scores
Bottom 0.3%
Top 0.02%
Top 0.0004%
recessive effect scores

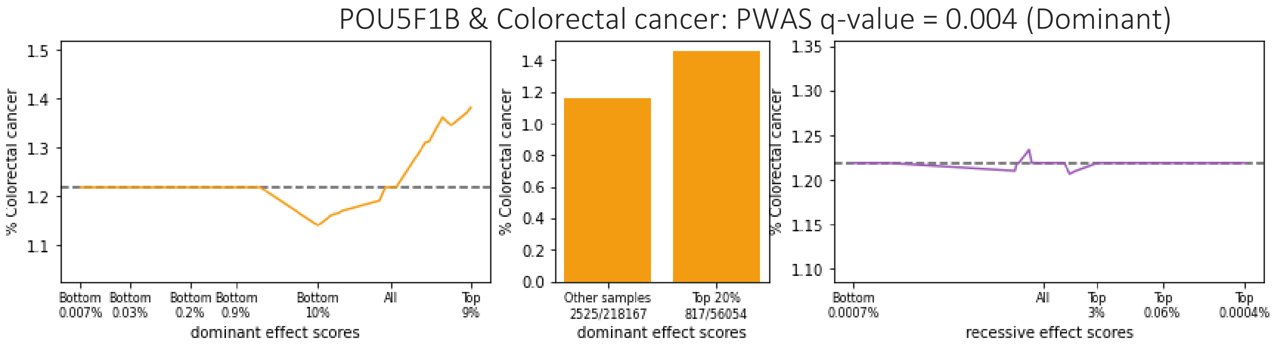
POU5F1B & Colorectal cancer: PWAS q-value = 0.004 (Dominant)
% Colorectal cancer
Bottom 0.007%
Bottom 0.03%
Bottom 0.2%
Bottom 0.9%
Bottom 10%
All
Top 9%
dominant effect scores
Other samples 2525/218167
Top 20% 817/56054
dominant effect scores
Bottom 0.0007%
All
Top 3%
Top 0.06%
Top 0.0004%
recessive effect scores

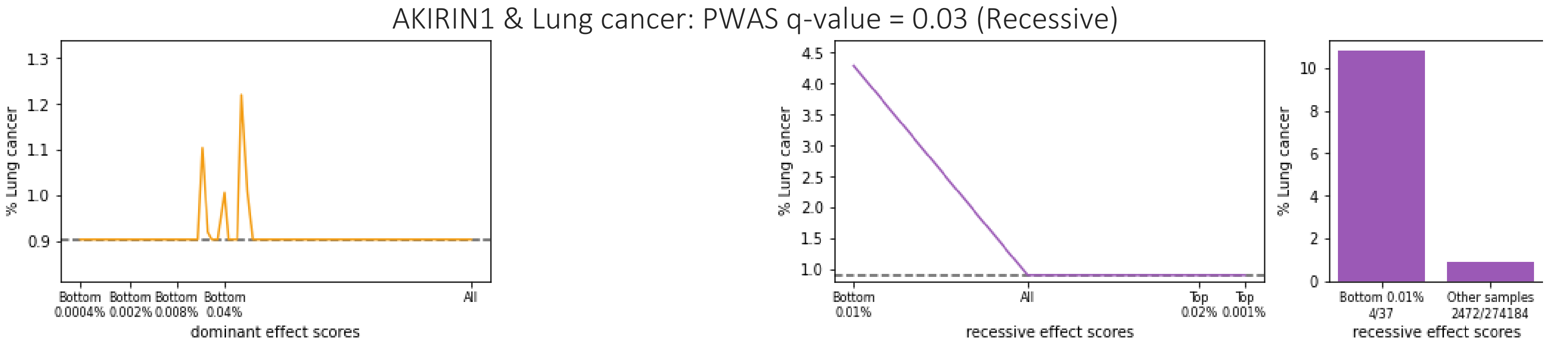
AKIRIN1 & Lung cancer: PWAS q-value = 0.03 (Recessive)
% Lung cancer
Bottom 0.0004%
Bottom 0.002%
Bottom 0.008%
Bottom 0.04%
All
dominant effect scores
Bottom 0.01%
All
Top 0.02%
Top 0.001%
recessive effect scores
Bottom 0.01% 4/37
Other samples 2472/274184
recessive effect scores

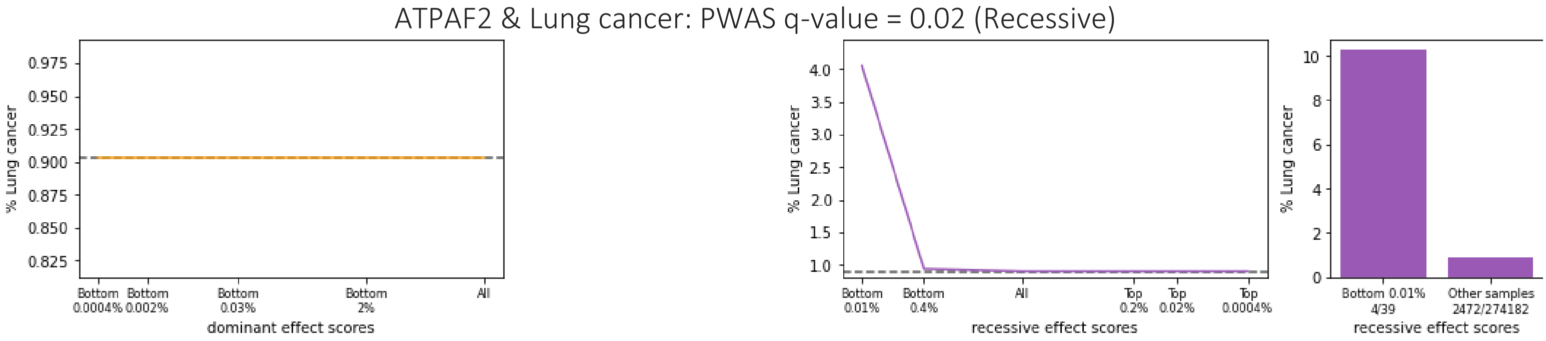
ATPAF2 & Lung cancer: PWAS q-value = 0.02 (Recessive)
% Lung cancer
Bottom 0.0004%
Bottom 0.002%
Bottom 0.03%
Bottom 2%
All
dominant effect scores
Bottom 0.01%
Bottom 0.4%
All
Top 0.2%
Top 0.02%
Top 0.0004%
recessive effect scores
Bottom 0.01% 4/39
Other samples 2472/274182
recessive effect scores

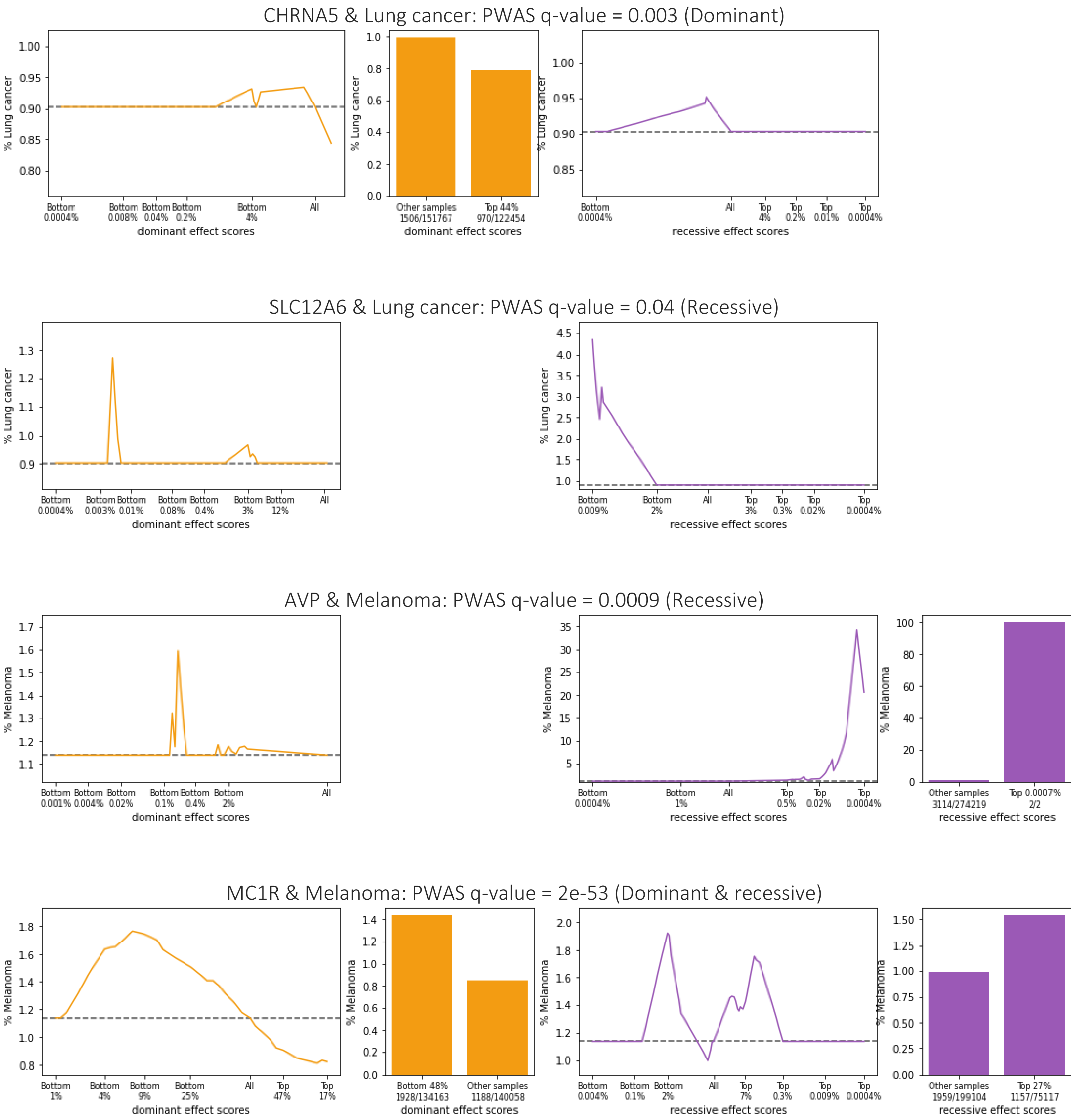
CHRNA5 & Lung cancer: PWAS q-value = 0.003 (Dominant)
% Lung cancer
Bottom 0.0004%
Bottom 0.008%
Bottom 0.04%
Bottom 0.2%
Bottom 4%
All
dominant effect scores
Other samples 1506/151767
Top 44% 970/122454
dominant effect scores
Top 4%
Top 0.2%
Top 0.01%
Top 0.0004%
recessive effect scores
SLC12A6 & Lung cancer: PWAS q-value = 0.04 (Recessive)
Bottom 0.003%
Bottom 0.01%
Bottom 0.08%
Bottom 0.4%
Bottom 3%
Bottom 12%
Bottom 0.009%
Bottom 2%
Top 3%
Top 0.3%
Top 0.02%
AVP & Melanoma: PWAS q-value = 0.0009 (Recessive)
% Melanoma
Bottom 0.001%
Bottom 0.004%
Bottom 0.02%
Bottom 0.1%
Bottom 0.4%
Bottom 1%
Top 0.5%
Other samples 3114/274219
Top 0.0007% 2/2
MC1R & Melanoma: PWAS q-value = 2e-53 (Dominant & recessive)
Bottom 9%
Bottom 25%
Top 47%
Top 17%
Bottom 48% 1928/134163
Other samples 1188/140058
Bottom 0.004%
Bottom 0.1%
Top 7%
Top 0.009%
Other samples 1959/199104
Top 27% 1157/75117

MITF & Melanoma: PWAS q-value = 1e-08 (Dominant & recessive)

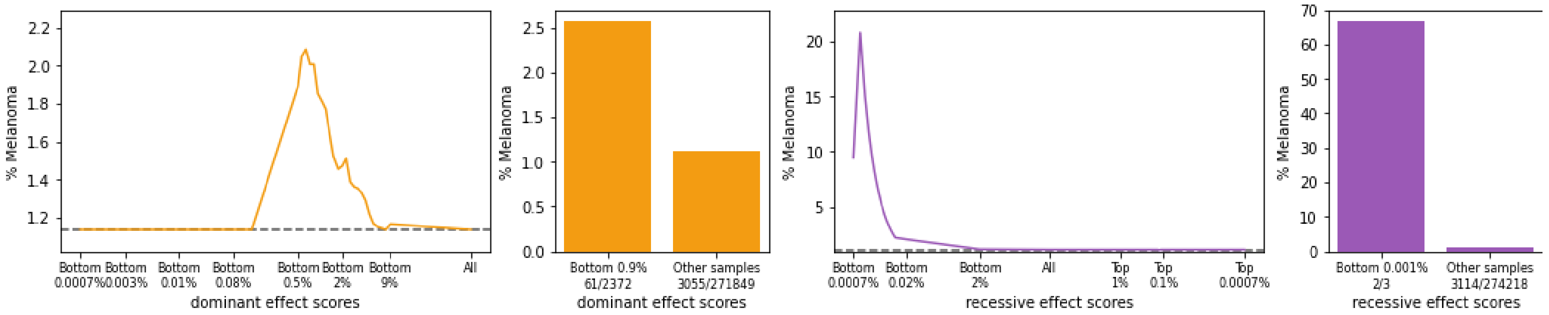

MTAP & Melanoma: PWAS q-value = 0.0001 (Dominant & recessive)

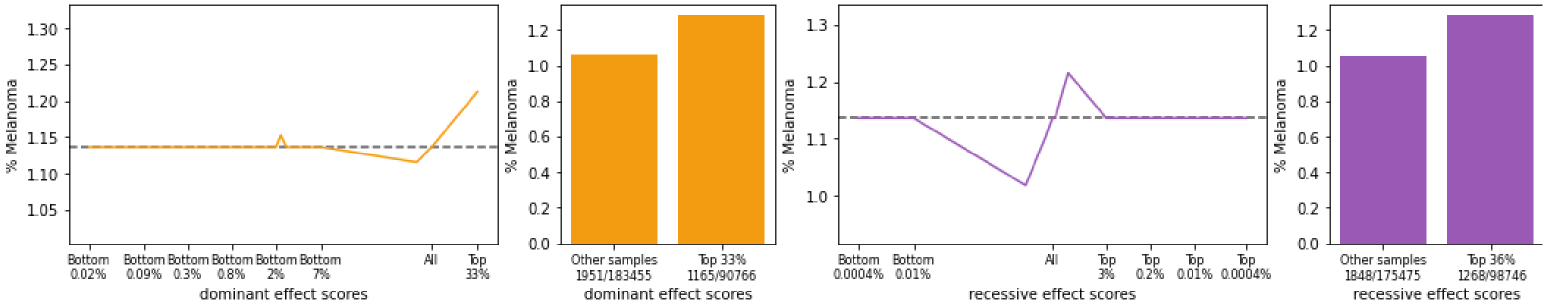

NCOA6 & Melanoma: PWAS q-value = 0.01 (Recessive)

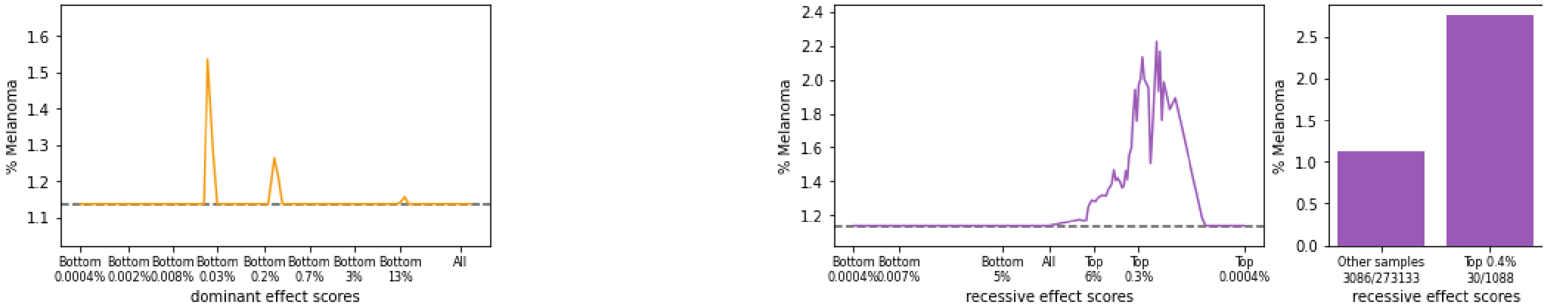

PADI1 & Melanoma: PWAS q-value = 0.004 (Recessive)

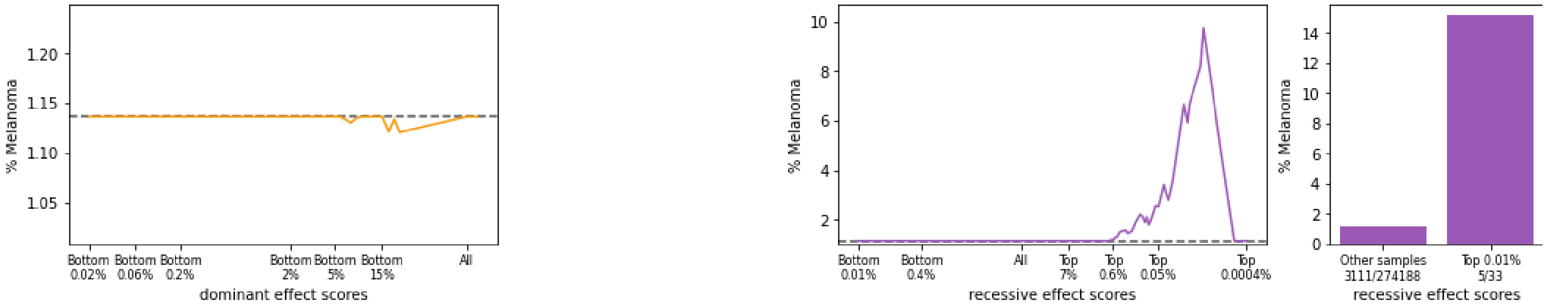

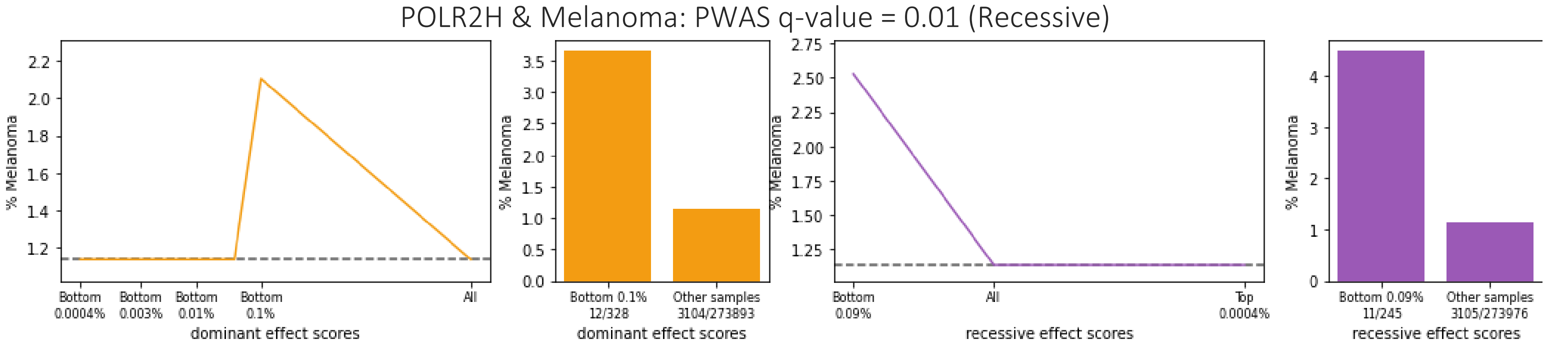
POLR2H & Melanoma: PWAS q-value = 0.01 (Recessive)
% Melanoma
Bottom 0.0004%
Bottom 0.003%
Bottom 0.01%
Bottom 0.1%
All
dominant effect scores
Bottom 0.1% 12/328
Other samples 3104/273893
dominant effect scores
Bottom 0.09%
All
Top 0.0004%
recessive effect scores
Bottom 0.09% 11/245
Other samples 3105/273976
recessive effect scores

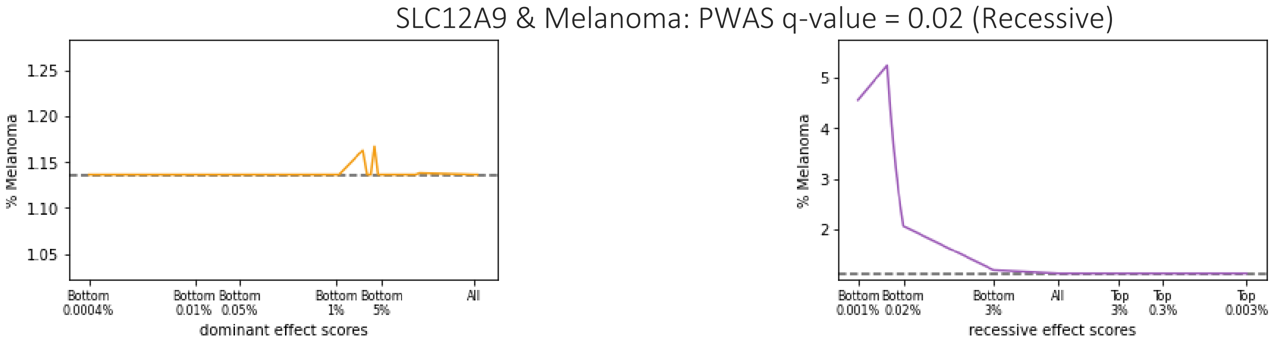
SLC12A9 & Melanoma: PWAS q-value = 0.02 (Recessive)
% Melanoma
Bottom 0.0004%
Bottom 0.01%
Bottom 0.05%
Bottom 1%
Bottom 5%
All
dominant effect scores
Bottom 0.001%
Bottom 0.02%
Bottom 3%
All
Top 3%
Top 0.3%
Top 0.003%
recessive effect scores

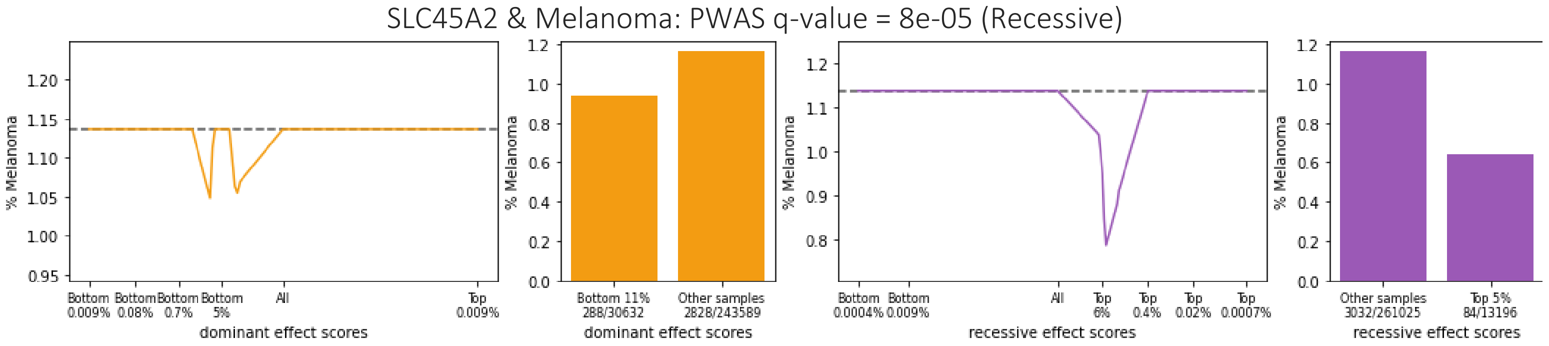
SLC45A2 & Melanoma: PWAS q-value = 8e-05 (Recessive)
% Melanoma
Bottom 0.009%
Bottom 0.08%
Bottom 0.7%
Bottom 5%
All
Top 0.009%
dominant effect scores
Bottom 11% 288/30632
Other samples 2828/243589
dominant effect scores
Bottom 0.0004%
Bottom 0.009%
All
Top 6%
Top 0.4%
Top 0.02%
Top 0.0007%
recessive effect scores
Other samples 3032/261025
Top 5% 84/13196
recessive effect scores

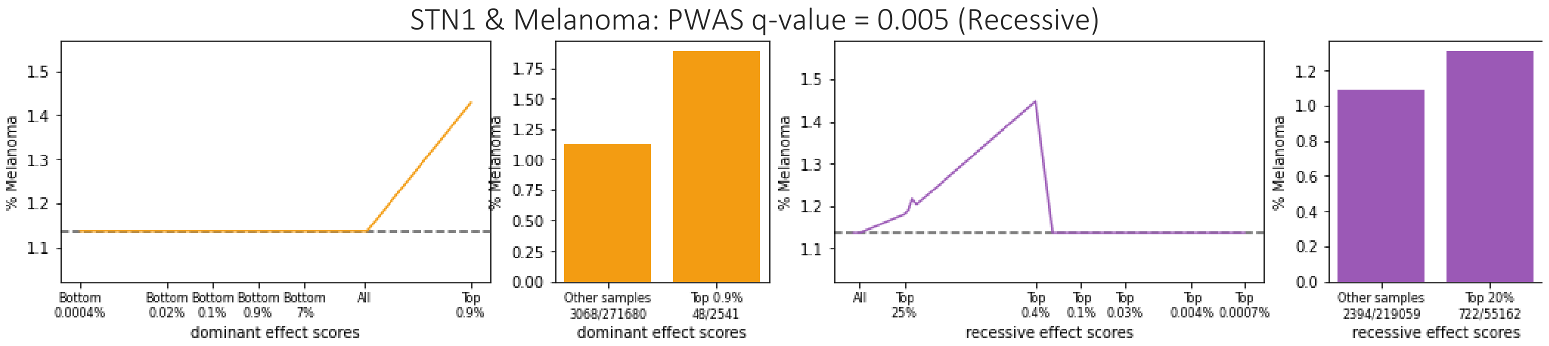
STN1 & Melanoma: PWAS q-value = 0.005 (Recessive)
% Melanoma
Bottom 0.0004%
Bottom 0.02%
Bottom 0.1%
Bottom 0.9%
Bottom 7%
All
Top 0.9%
dominant effect scores
Other samples 3068/271680
Top 0.9% 48/2541
dominant effect scores
All
Top 25%
Top 0.4%
Top 0.1%
Top 0.03%
Top 0.004%
Top 0.0007%
recessive effect scores
Other samples 2394/219059
Top 20% 722/55162
recessive effect scores

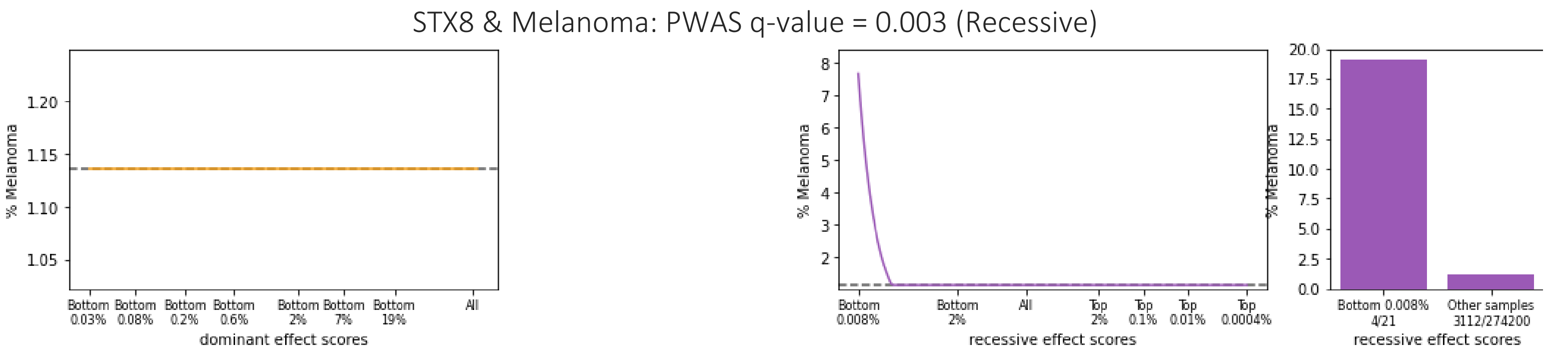
STX8 & Melanoma: PWAS q-value = 0.003 (Recessive)
% Melanoma
dominant effect scores
recessive effect scores
Bottom 0.008% 4/21
Other samples 3112/274200

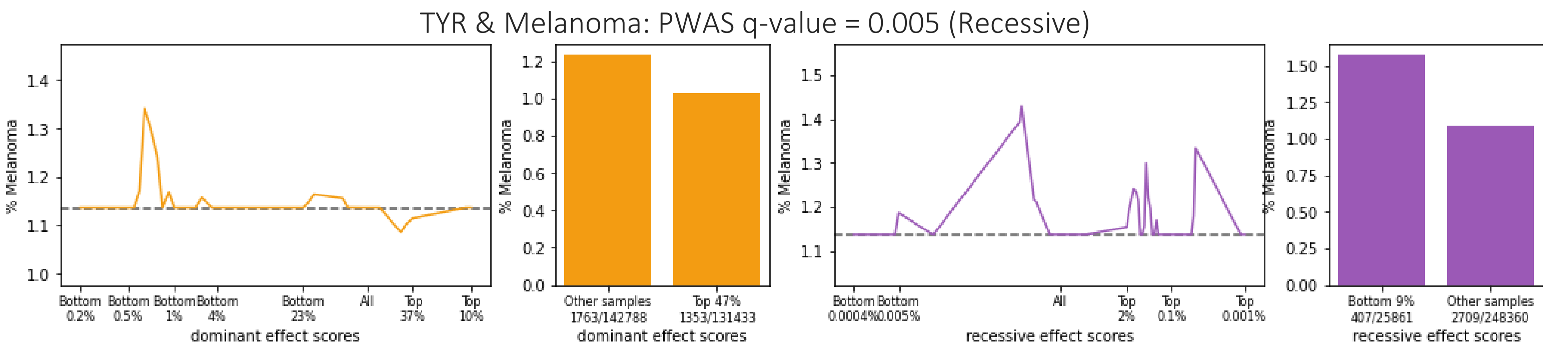
TYR & Melanoma: PWAS q-value = 0.005 (Recessive)
% Melanoma
dominant effect scores
Other samples 1763/142788
Top 47% 1353/131433
recessive effect scores
Bottom 9% 407/25861
Other samples 2709/248360

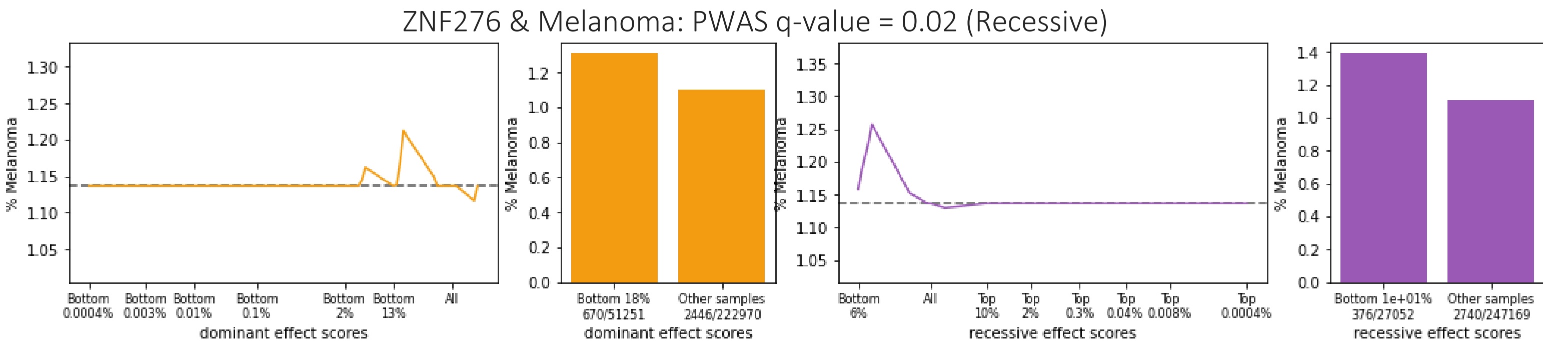
ZNF276 & Melanoma: PWAS q-value = 0.02 (Recessive)
% Melanoma
dominant effect scores
Bottom 18% 670/51251
Other samples 2446/222970
recessive effect scores
Bottom 1e+01% 376/27052
Other samples 2740/247169

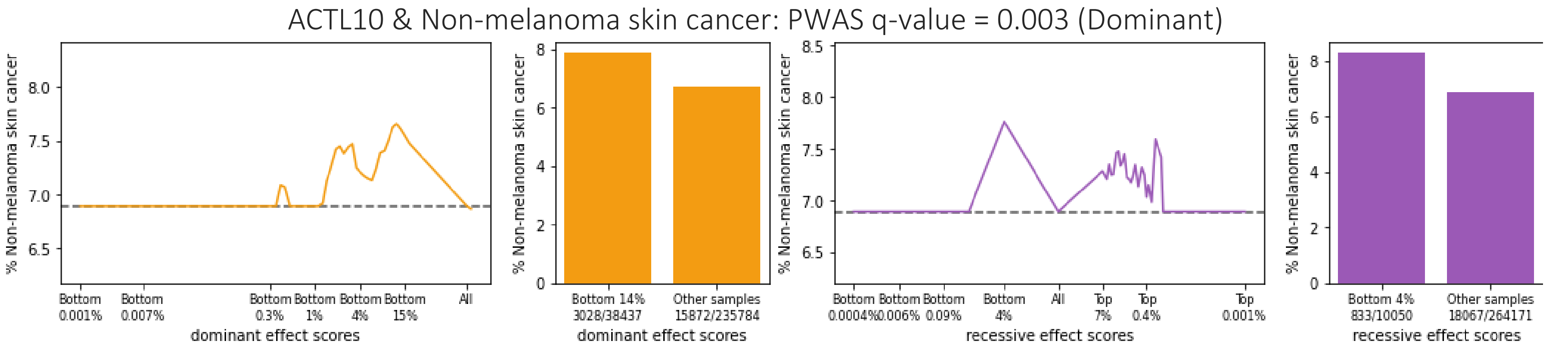
ACTL10 & Non-melanoma skin cancer: PWAS q-value = 0.003 (Dominant)
% Non-melanoma skin cancer
dominant effect scores
Bottom 14% 3028/38437
Other samples 15872/235784
recessive effect scores
Bottom 4% 833/10050
Other samples 18067/264171

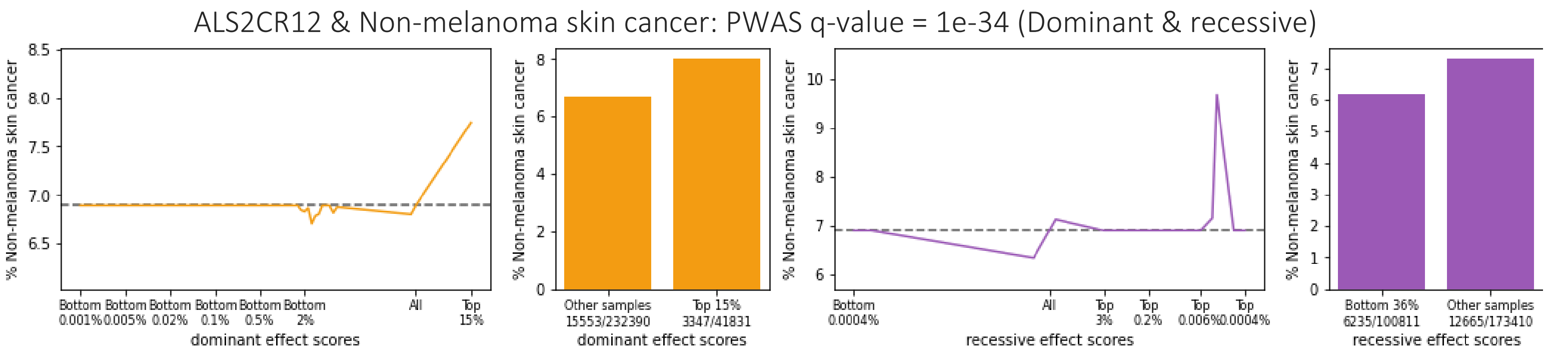
ALS2CR12 & Non-melanoma skin cancer: PWAS q-value = 1e-34 (Dominant & recessive)
% Non-melanoma skin cancer
Bottom 0.001% Bottom 0.005% Bottom 0.02% Bottom 0.1% Bottom 0.5% Bottom 2% All Top 15%
dominant effect scores
Other samples 15553/232390
Top 15% 3347/41831
dominant effect scores
Bottom 0.0004% All Top 3% Top 0.2% Top 0.006% Top 0.0004%
recessive effect scores
Bottom 36% 6235/100811
Other samples 12665/173410
recessive effect scores

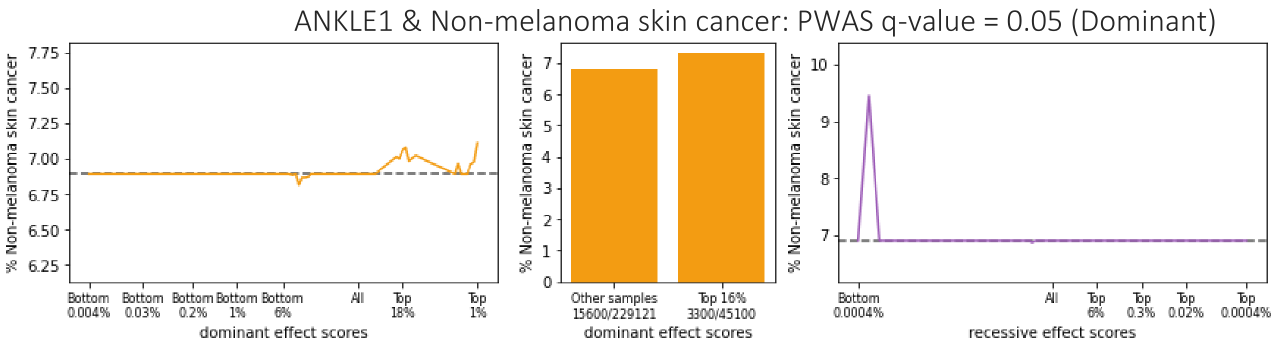
ANKLE1 & Non-melanoma skin cancer: PWAS q-value = 0.05 (Dominant)
% Non-melanoma skin cancer
Bottom 0.004% Bottom 0.03% Bottom 0.2% Bottom 1% Bottom 6% All Top 18% Top 1%
dominant effect scores
Other samples 15600/229121
Top 16% 3300/45100
dominant effect scores
Bottom 0.0004% All Top 6% Top 0.3% Top 0.02% Top 0.0004%
recessive effect scores

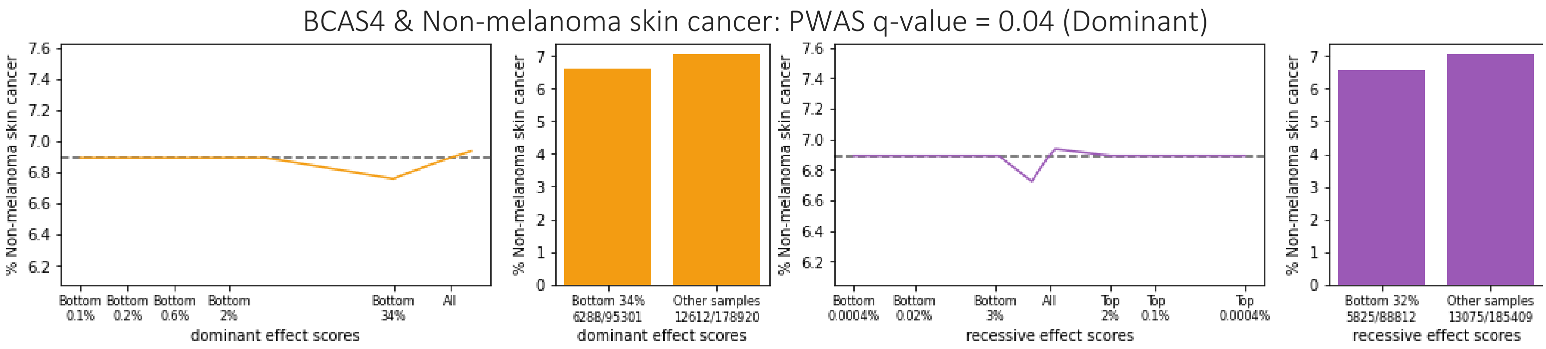
BCAS4 & Non-melanoma skin cancer: PWAS q-value = 0.04 (Dominant)
% Non-melanoma skin cancer
Bottom 0.1% Bottom 0.2% Bottom 0.6% Bottom 2% Bottom 34% All
dominant effect scores
Bottom 34% 6288/95301
Other samples 12612/178920
dominant effect scores
Bottom 0.0004% Bottom 0.02% Bottom 3% All Top 2% Top 0.1% Top 0.0004%
recessive effect scores
Bottom 32% 5825/88812
Other samples 13075/185409
recessive effect scores

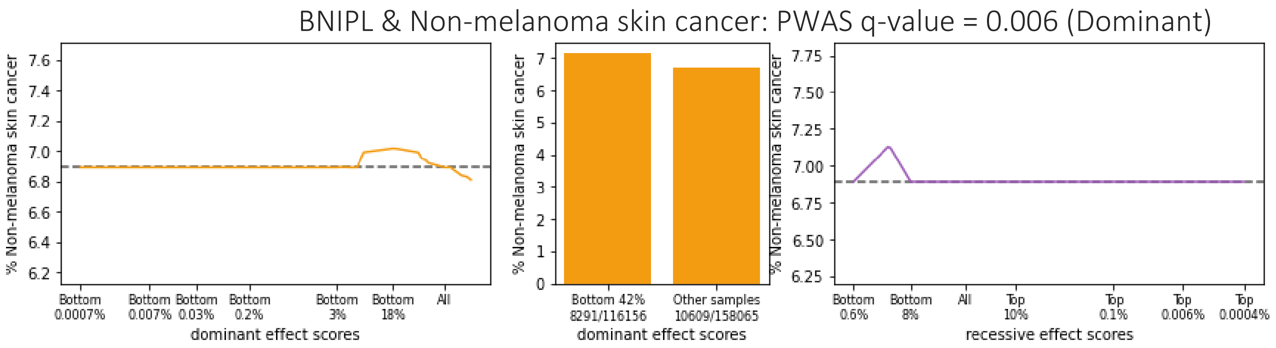
BNIPL & Non-melanoma skin cancer: PWAS q-value = 0.006 (Dominant)
% Non-melanoma skin cancer
Bottom 0.0007% Bottom 0.007% Bottom 0.03% Bottom 0.2% Bottom 3% Bottom 18% All
dominant effect scores
Bottom 42% 8291/116156
Other samples 10609/158065
dominant effect scores
Bottom 0.6% Bottom 8% All Top 10% Top 0.1% Top 0.006% Top 0.0004%
recessive effect scores

BPIFB3 & Non-melanoma skin cancer: PWAS q-value = 0.008 (Dominant)

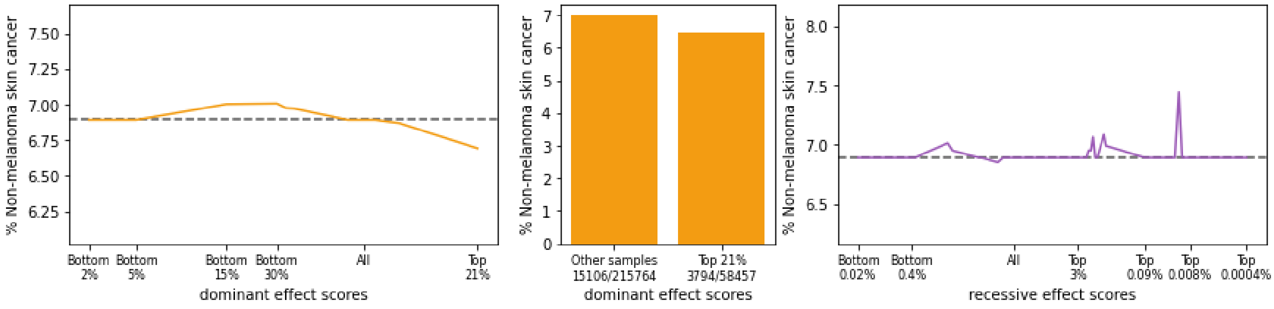

C6orf15 & Non-melanoma skin cancer: PWAS q-value = 0.03 (None)

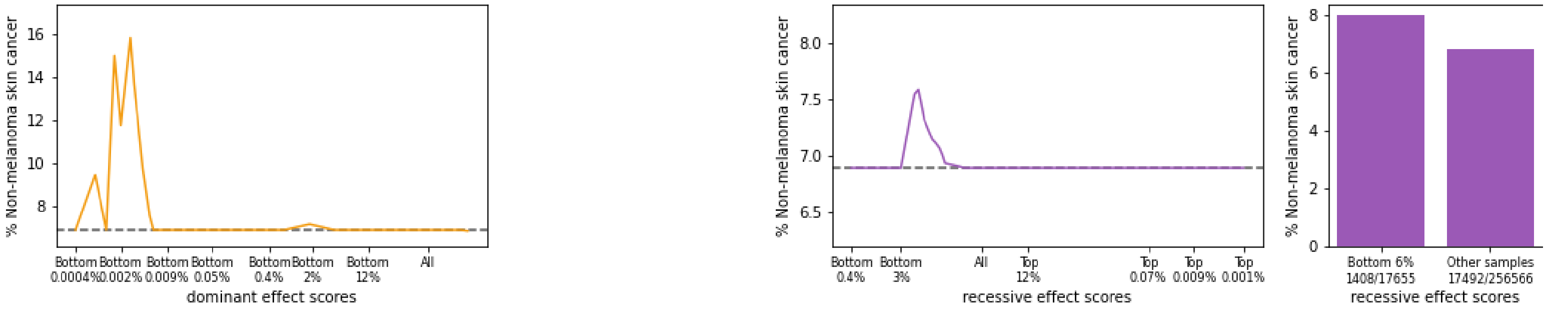

CCDC88B & Non-melanoma skin cancer: PWAS q-value = 0.02 (None)

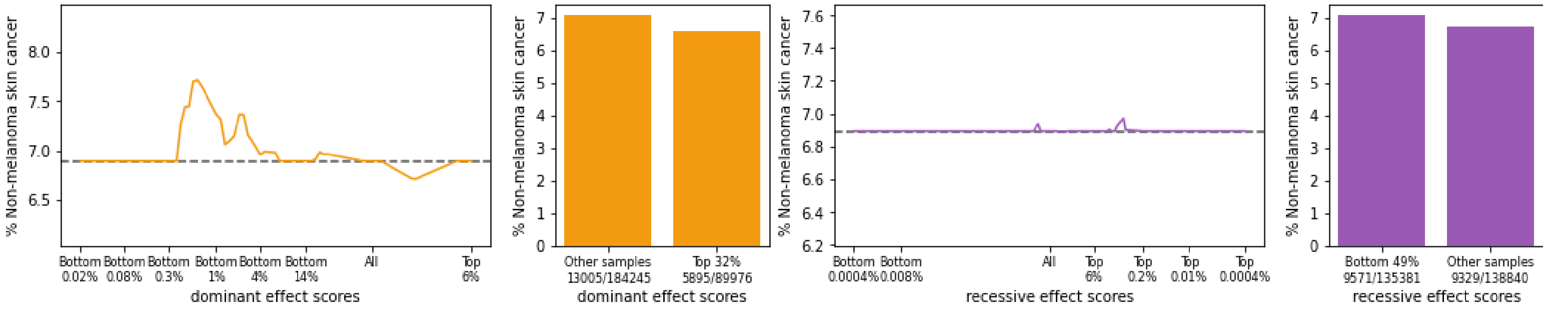

CCHCR1 & Non-melanoma skin cancer: PWAS q-value = 0.001 (Recessive)

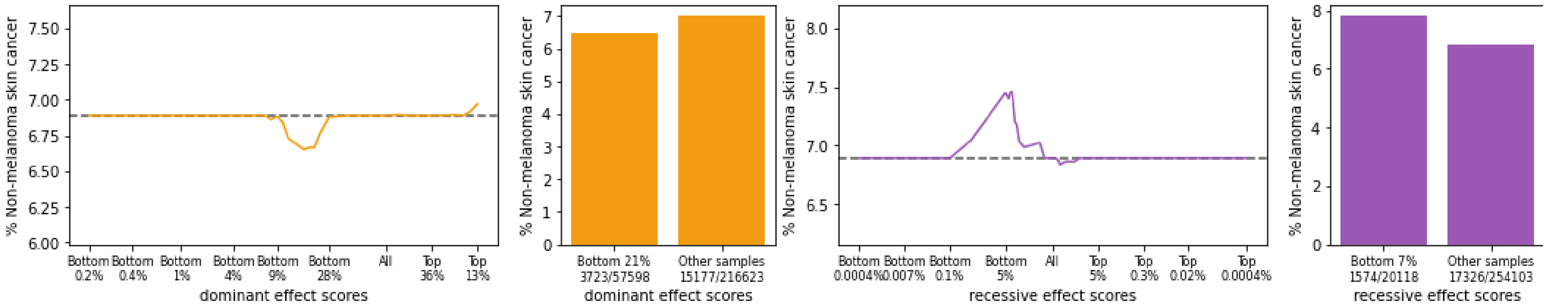

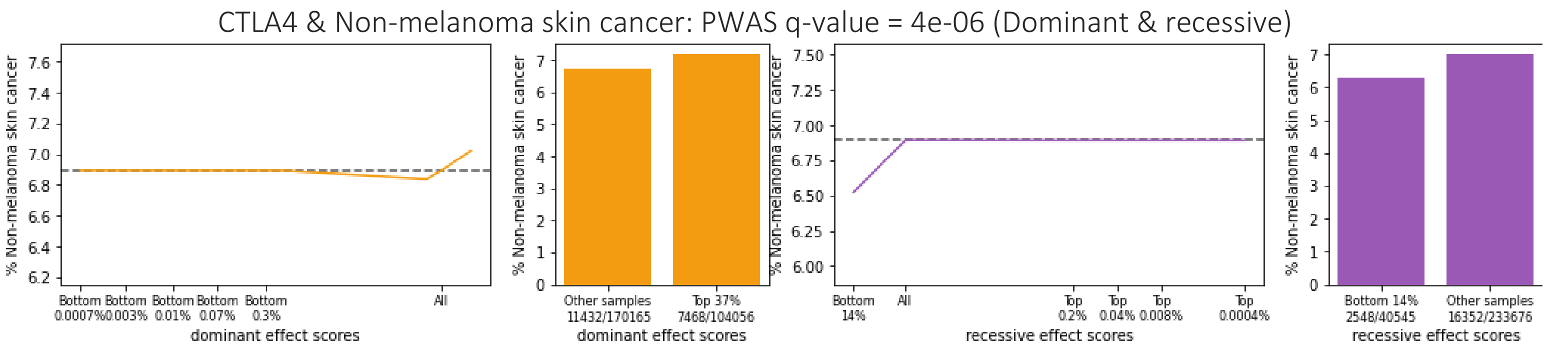
CTLA4 & Non-melanoma skin cancer: PWAS q-value = 4e-06 (Dominant & recessive)
% Non-melanoma skin cancer
Bottom 0.0007%
Bottom 0.003%
Bottom 0.01%
Bottom 0.07%
Bottom 0.3%
All
dominant effect scores
Other samples 11432/170165
Top 37% 7468/104056
dominant effect scores
Bottom 14%
All
Top 0.2%
Top 0.04%
Top 0.008%
Top 0.0004%
recessive effect scores
Bottom 14% 2548/40545
Other samples 16352/233676
recessive effect scores

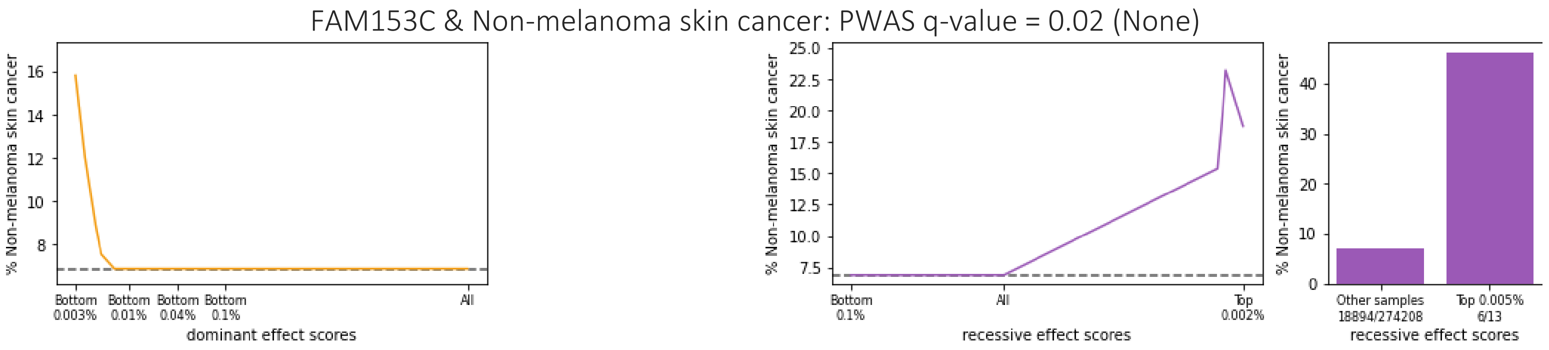
FAM153C & Non-melanoma skin cancer: PWAS q-value = 0.02 (None)
% Non-melanoma skin cancer
Bottom 0.003%
Bottom 0.01%
Bottom 0.04%
Bottom 0.1%
All
dominant effect scores
Bottom 0.1%
All
Top 0.002%
recessive effect scores
Other samples 18894/274208
Top 0.005% 6/13
recessive effect scores

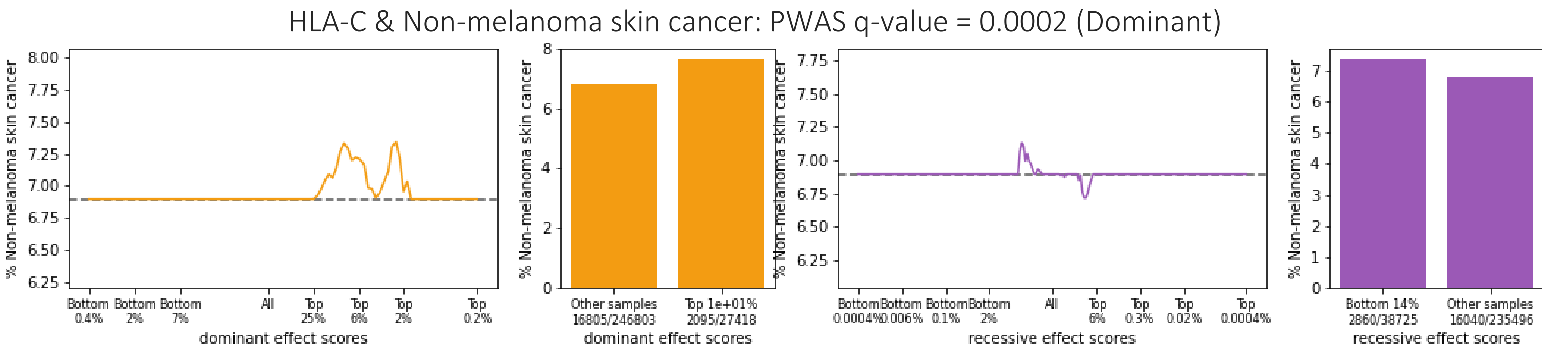
HLA-C & Non-melanoma skin cancer: PWAS q-value = 0.0002 (Dominant)
% Non-melanoma skin cancer
Bottom 0.4%
Bottom 2%
Bottom 7%
All
Top 25%
Top 6%
Top 2%
Top 0.2%
dominant effect scores
Other samples 16805/246803
Top 1e+01% 2095/27418
dominant effect scores
Bottom 0.0004%
Bottom 0.006%
Bottom 0.1%
Bottom 2%
All
Top 6%
Top 0.3%
Top 0.02%
Top 0.0004%
recessive effect scores
Bottom 14% 2860/38725
Other samples 16040/235496
recessive effect scores

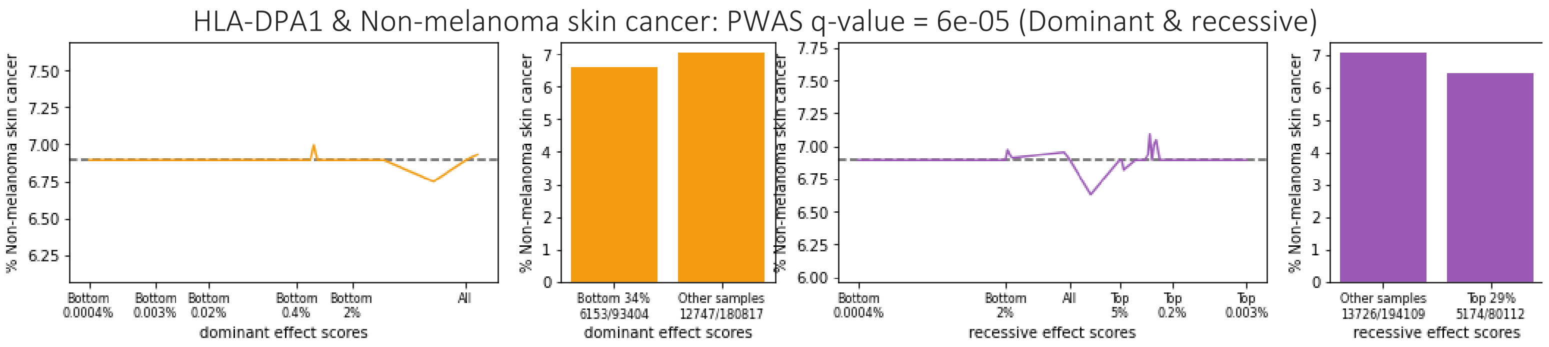
HLA-DPA1 & Non-melanoma skin cancer: PWAS q-value = 6e-05 (Dominant & recessive)
% Non-melanoma skin cancer
Bottom 0.0004%
Bottom 0.003%
Bottom 0.02%
Bottom 0.4%
Bottom 2%
All
dominant effect scores
Bottom 34% 6153/93404
Other samples 12747/180817
dominant effect scores
Bottom 0.0004%
Bottom 2%
All
Top 5%
Top 0.2%
Top 0.003%
recessive effect scores
Other samples 13726/194109
Top 29% 5174/80112
recessive effect scores

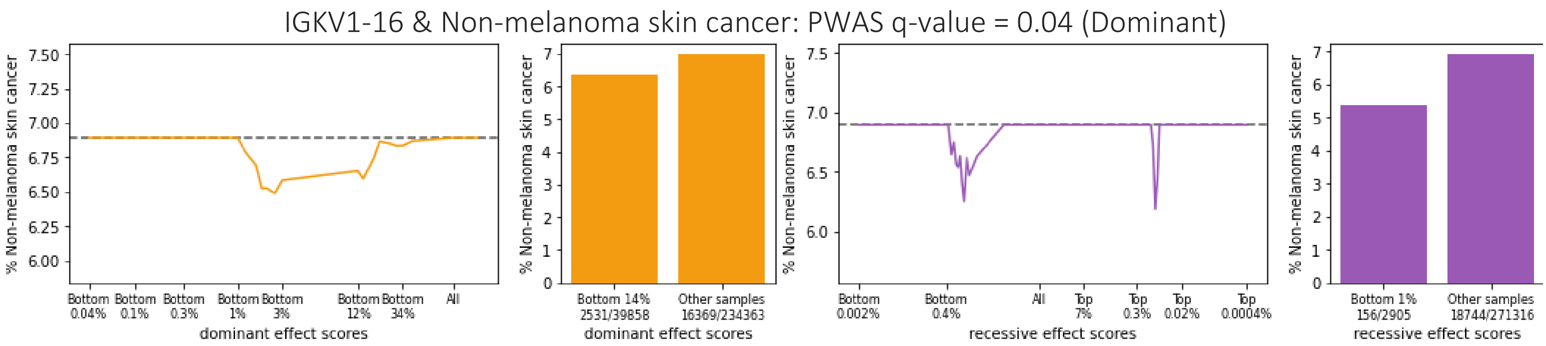
IGKV1-16 & Non-melanoma skin cancer: PWAS q-value = 0.04 (Dominant)
% Non-melanoma skin cancer
dominant effect scores
Bottom 14% 2531/39858
Other samples 16369/234363
recessive effect scores
Bottom 1% 156/2905
Other samples 18744/271316

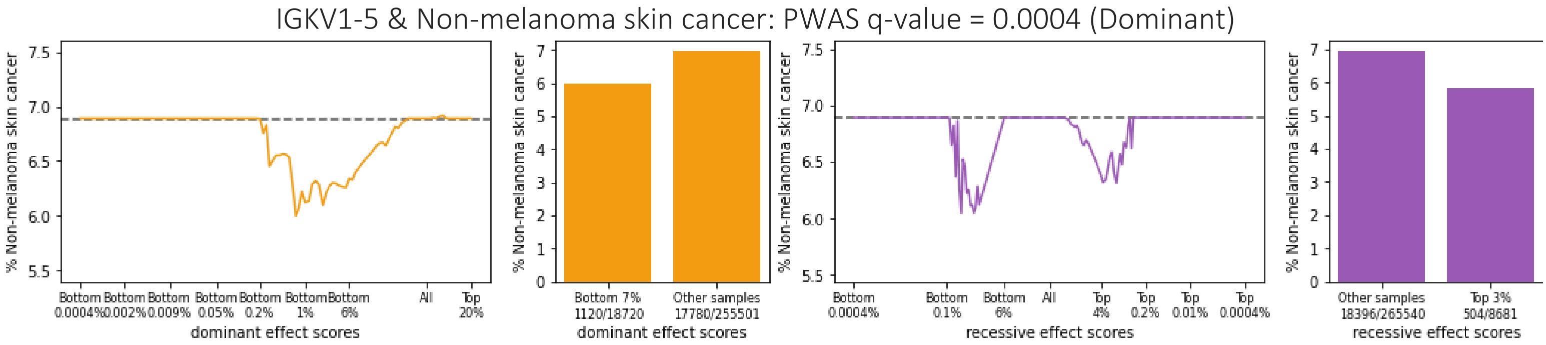
IGKV1-5 & Non-melanoma skin cancer: PWAS q-value = 0.0004 (Dominant)
% Non-melanoma skin cancer
dominant effect scores
Bottom 7% 1120/18720
Other samples 17780/255501
recessive effect scores
Other samples 18396/265540
Top 3% 504/8681

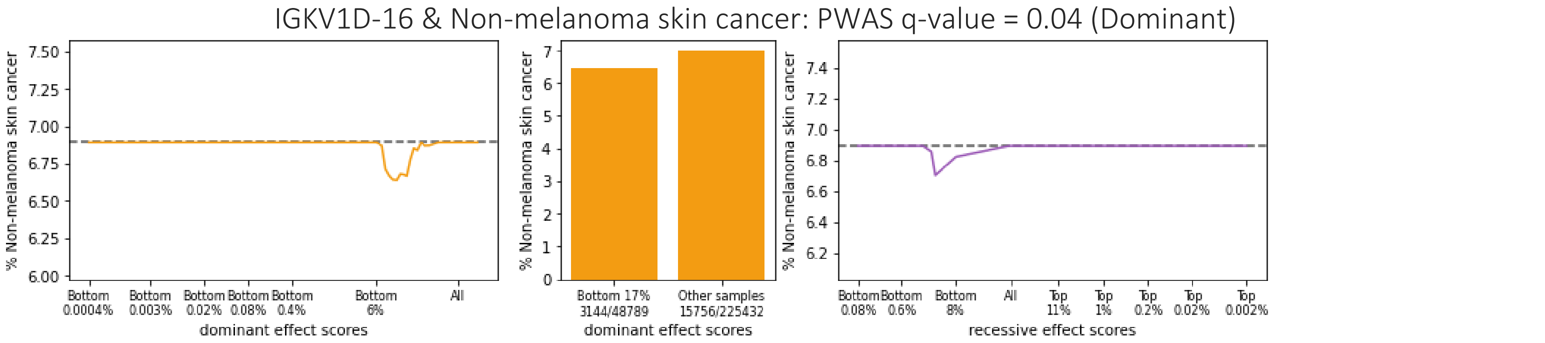
IGKV1D-16 & Non-melanoma skin cancer: PWAS q-value = 0.04 (Dominant)
% Non-melanoma skin cancer
dominant effect scores
Bottom 17% 3144/48789
Other samples 15756/225432
recessive effect scores

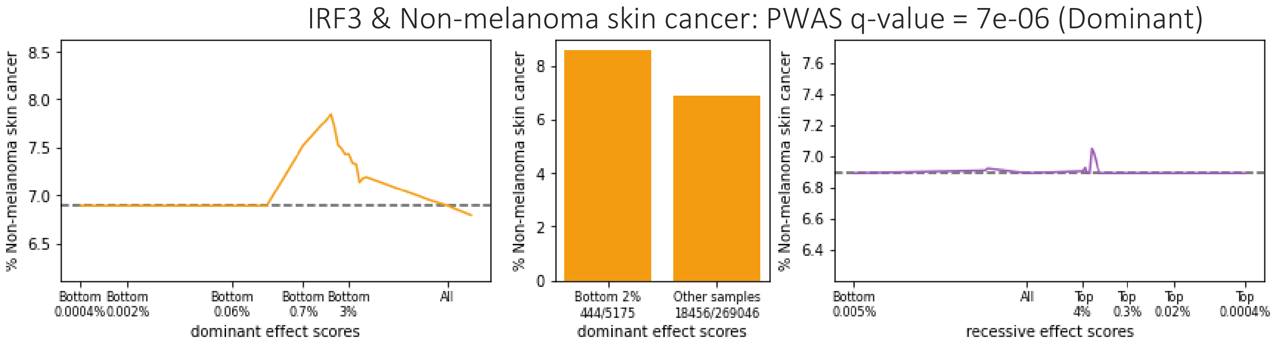
IRF3 & Non-melanoma skin cancer: PWAS q-value = 7e-06 (Dominant)
% Non-melanoma skin cancer
dominant effect scores
Bottom 2% 444/5175
Other samples 18456/269046
recessive effect scores

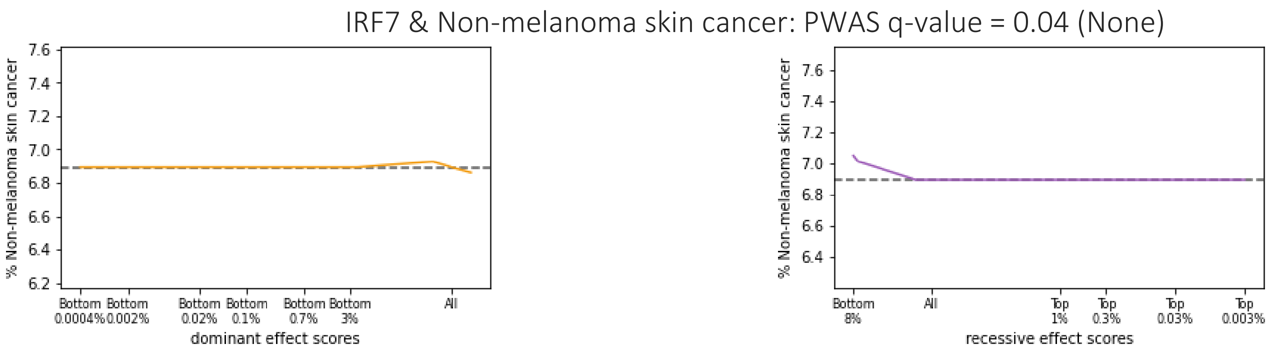
IRF7 & Non-melanoma skin cancer: PWAS q-value = 0.04 (None)
% Non-melanoma skin cancer
Bottom 0.0004%
Bottom 0.002%
Bottom 0.02%
Bottom 0.1%
Bottom 0.7%
Bottom 3%
All
dominant effect scores
Bottom 8%
All
Top 1%
Top 0.3%
Top 0.03%
Top 0.003%
recessive effect scores

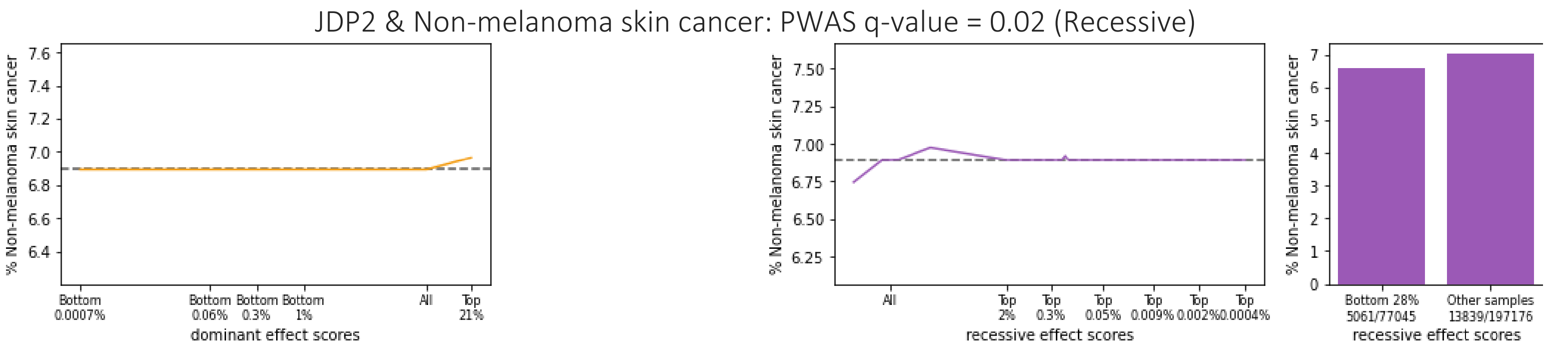
JDP2 & Non-melanoma skin cancer: PWAS q-value = 0.02 (Recessive)
% Non-melanoma skin cancer
Bottom 0.0007%
Bottom 0.06%
Bottom 0.3%
Bottom 1%
All
Top 21%
dominant effect scores
All
Top 2%
Top 0.3%
Top 0.05%
Top 0.009%
Top 0.002%
Top 0.0004%
recessive effect scores
Bottom 28% 5061/77045
Other samples 13839/197176
recessive effect scores

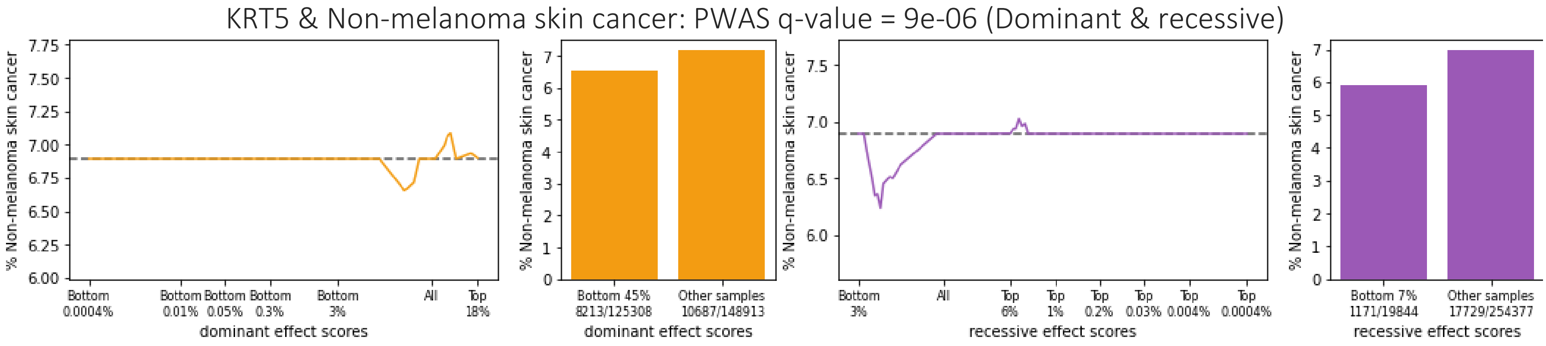
KRT5 & Non-melanoma skin cancer: PWAS q-value = 9e-06 (Dominant & recessive)
% Non-melanoma skin cancer
Bottom 0.0004%
Bottom 0.01%
Bottom 0.05%
Bottom 0.3%
Bottom 3%
All
Top 18%
dominant effect scores
Bottom 45% 8213/125308
Other samples 10687/148913
dominant effect scores
Bottom 3%
All
Top 6%
Top 1%
Top 0.2%
Top 0.03%
Top 0.004%
Top 0.0004%
recessive effect scores
Bottom 7% 1171/19844
Other samples 17729/254377
recessive effect scores

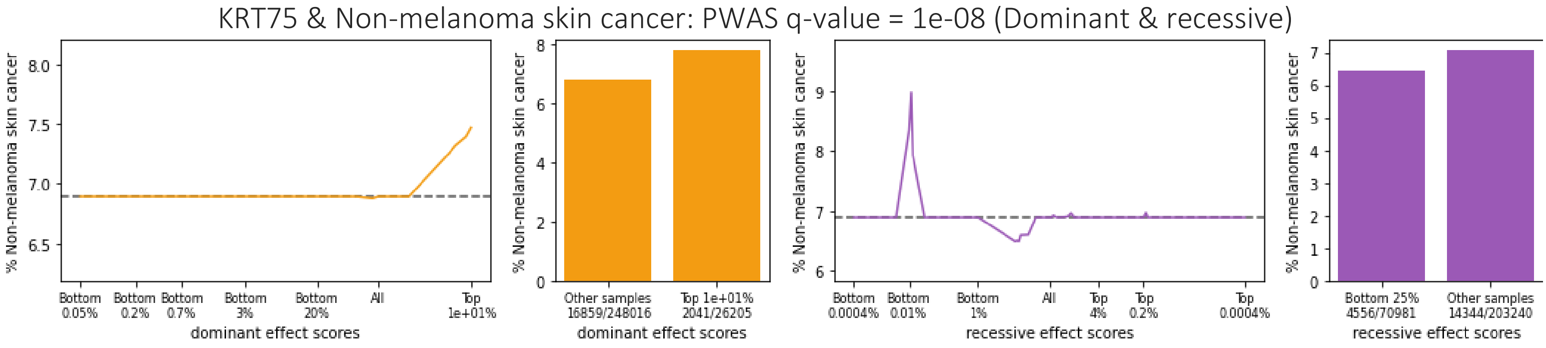
KRT75 & Non-melanoma skin cancer: PWAS q-value = 1e-08 (Dominant & recessive)
% Non-melanoma skin cancer
Bottom 0.05%
Bottom 0.2%
Bottom 0.7%
Bottom 3%
Bottom 20%
All
Top 1e+01%
dominant effect scores
Other samples 16859/248016
Top 1e+01% 2041/26205
dominant effect scores
Bottom 0.0004%
Bottom 0.01%
Bottom 1%
All
Top 4%
Top 0.2%
Top 0.0004%
recessive effect scores
Bottom 25% 4556/70981
Other samples 14344/203240
recessive effect scores

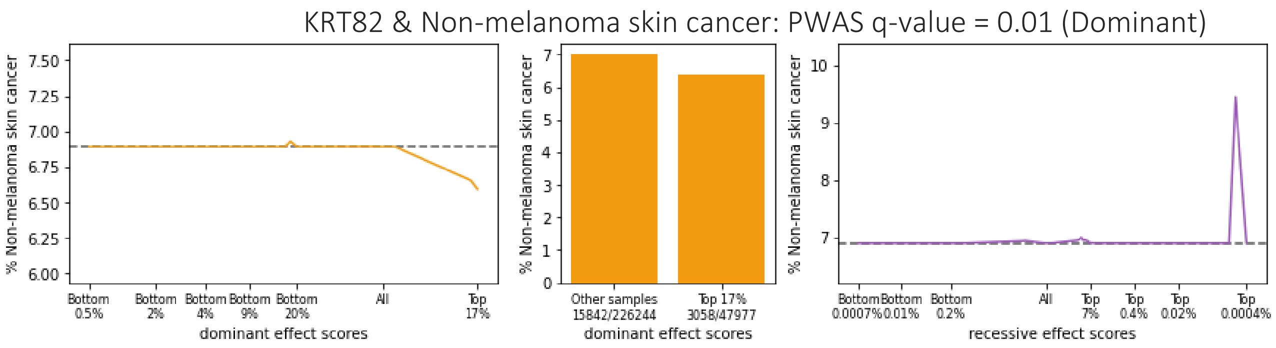
KRT82 & Non-melanoma skin cancer: PWAS q-value = 0.01 (Dominant)
% Non-melanoma skin cancer
dominant effect scores
Other samples 15842/226244
Top 17% 3058/47977
recessive effect scores

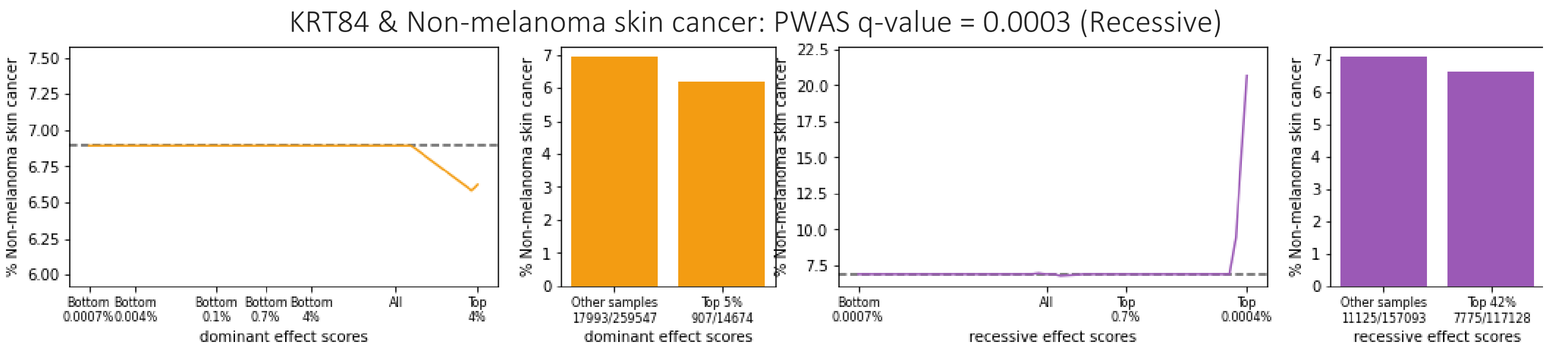
KRT84 & Non-melanoma skin cancer: PWAS q-value = 0.0003 (Recessive)
% Non-melanoma skin cancer
dominant effect scores
Other samples 17993/259547
Top 5% 907/14674
recessive effect scores
Other samples 11125/157093
Top 42% 7775/117128

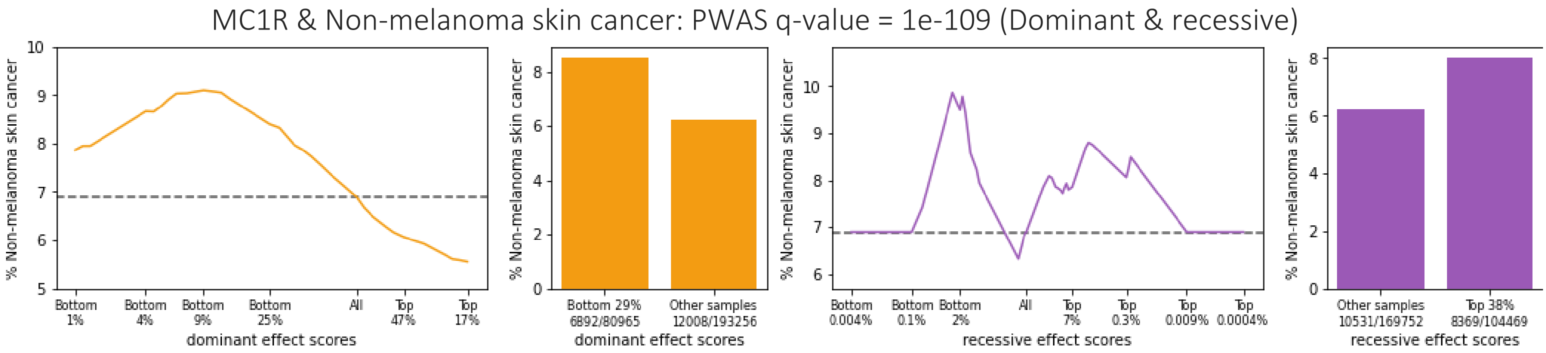
MC1R & Non-melanoma skin cancer: PWAS q-value = 1e-109 (Dominant & recessive)
% Non-melanoma skin cancer
dominant effect scores
Bottom 29% 6892/80965
Other samples 12008/193256
recessive effect scores
Other samples 10531/169752
Top 38% 8369/104469

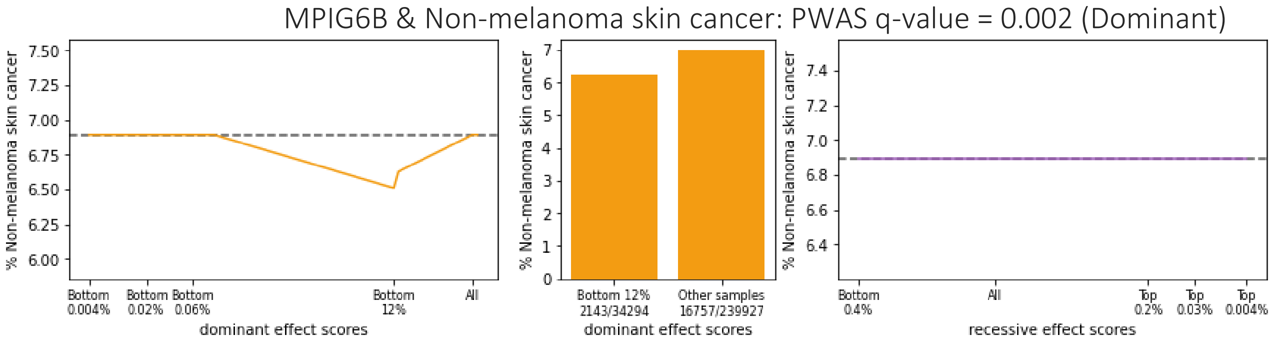
MPIG6B & Non-melanoma skin cancer: PWAS q-value = 0.002 (Dominant)
% Non-melanoma skin cancer
dominant effect scores
Bottom 12% 2143/34294
Other samples 16757/239927
recessive effect scores

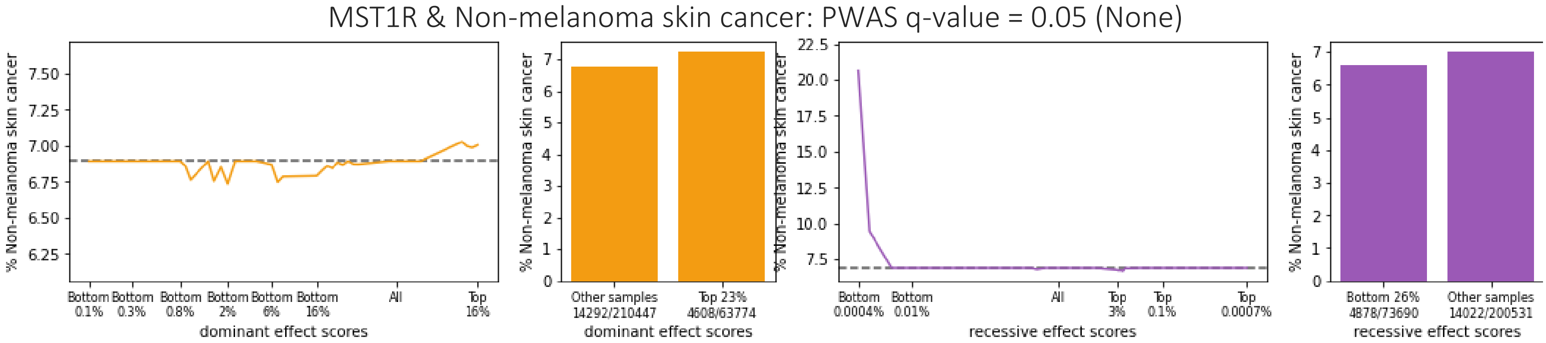
MST1R & Non-melanoma skin cancer: PWAS q-value = 0.05 (None)
% Non-melanoma skin cancer
dominant effect scores
Other samples 14292/210447
Top 23% 4608/63774
recessive effect scores
Bottom 26% 4878/73690
Other samples 14022/200531

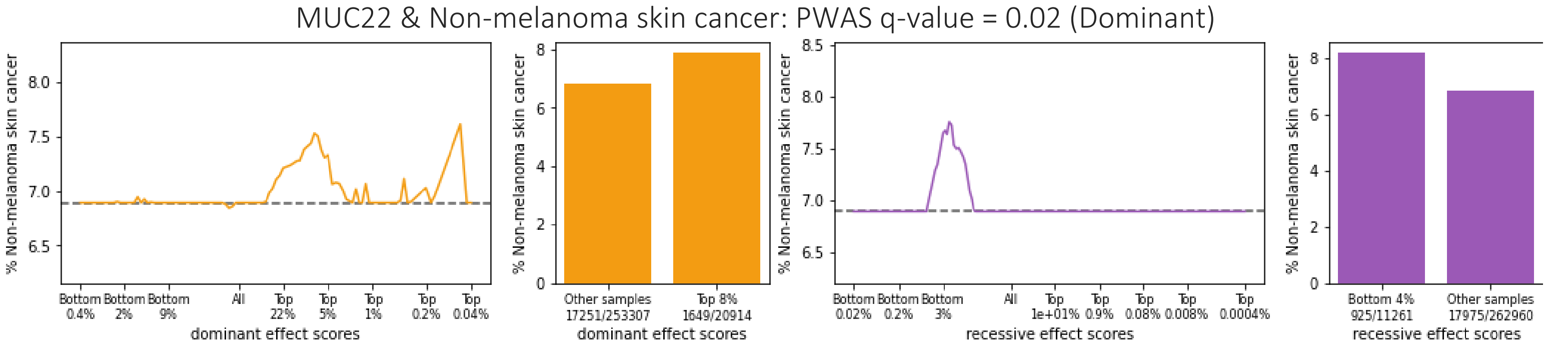
MUC22 & Non-melanoma skin cancer: PWAS q-value = 0.02 (Dominant)
% Non-melanoma skin cancer
dominant effect scores
Other samples 17251/253307
Top 8% 1649/20914
recessive effect scores
Bottom 4% 925/11261
Other samples 17975/262960

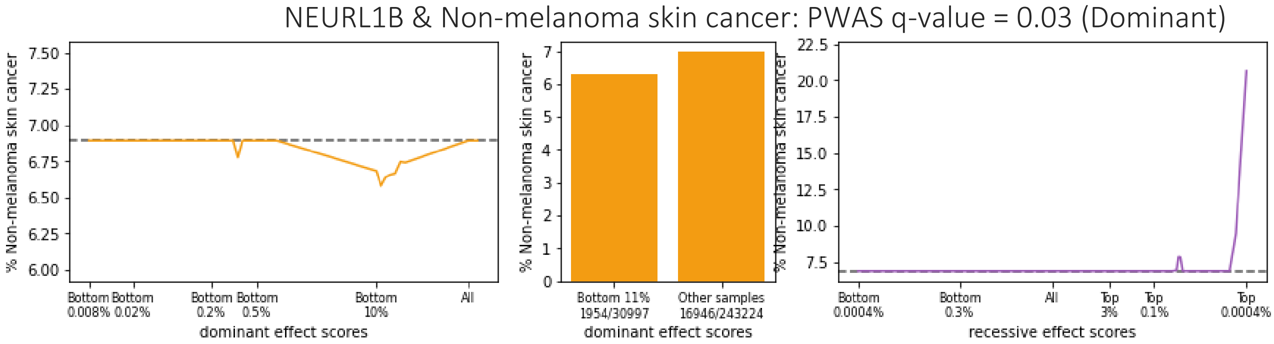
NEURL1B & Non-melanoma skin cancer: PWAS q-value = 0.03 (Dominant)
% Non-melanoma skin cancer
dominant effect scores
Bottom 11% 1954/30997
Other samples 16946/243224
recessive effect scores

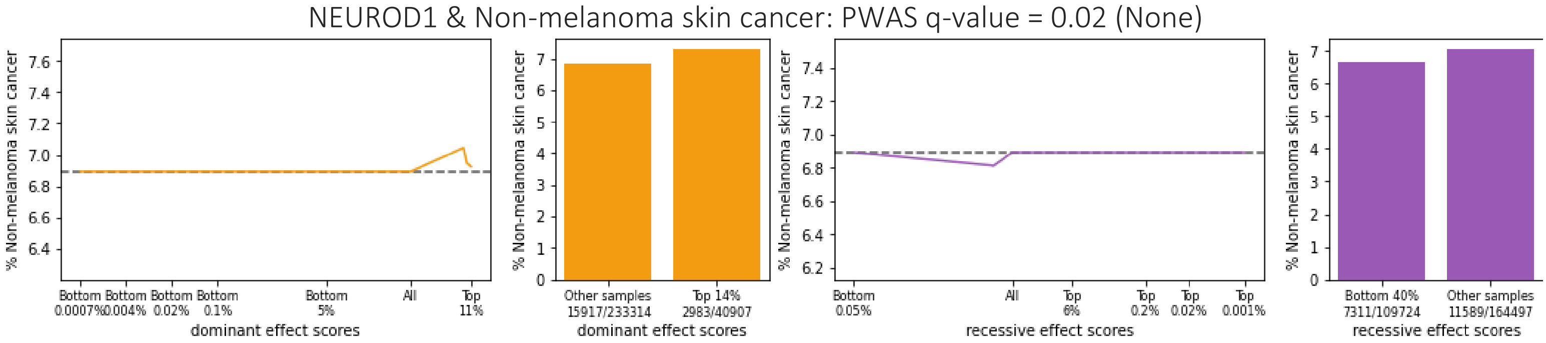
NEUROD1 & Non-melanoma skin cancer: PWAS q-value = 0.02 (None)
% Non-melanoma skin cancer
dominant effect scores
Other samples 15917/233314
Top 14% 2983/40907
recessive effect scores
Bottom 40% 7311/109724
Other samples 11589/164497

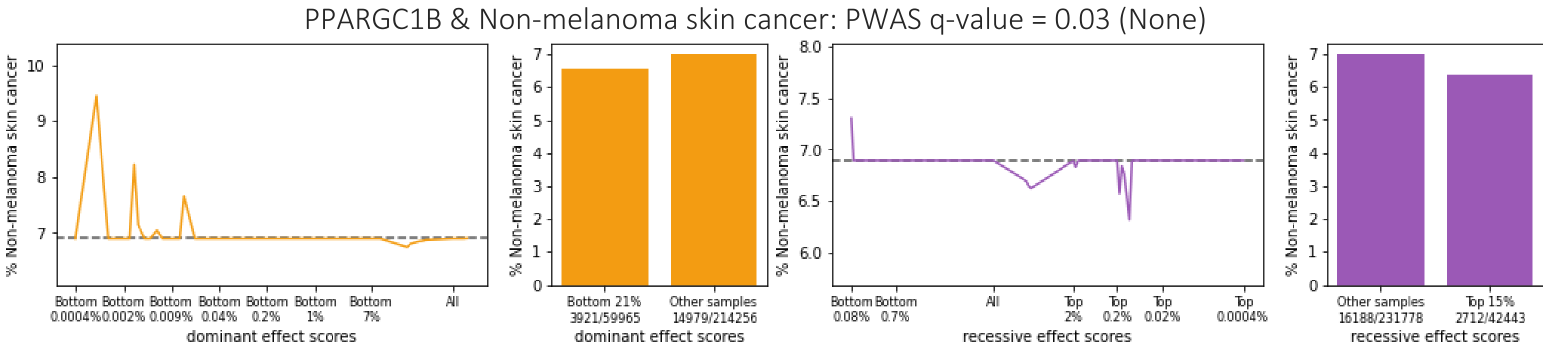
PPARGC1B & Non-melanoma skin cancer: PWAS q-value = 0.03 (None)
% Non-melanoma skin cancer
dominant effect scores
Bottom 21% 3921/59965
Other samples 14979/214256
recessive effect scores
Other samples 16188/231778
Top 15% 2712/42443

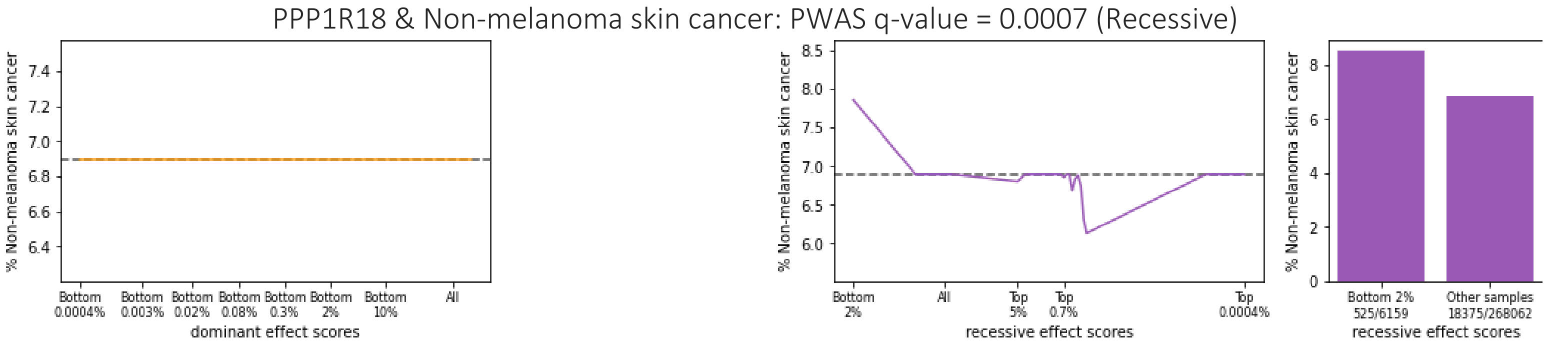
PPP1R18 & Non-melanoma skin cancer: PWAS q-value = 0.0007 (Recessive)
% Non-melanoma skin cancer
dominant effect scores
recessive effect scores
Bottom 2% 525/6159
Other samples 18375/268062

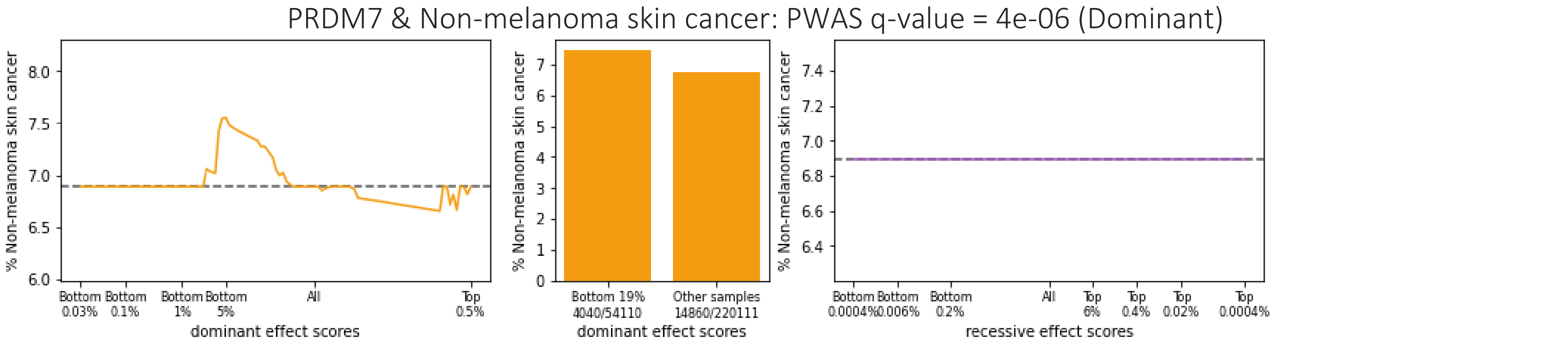
PRDM7 & Non-melanoma skin cancer: PWAS q-value = 4e-06 (Dominant)
% Non-melanoma skin cancer
dominant effect scores
Bottom 19% 4040/54110
Other samples 14860/220111
recessive effect scores

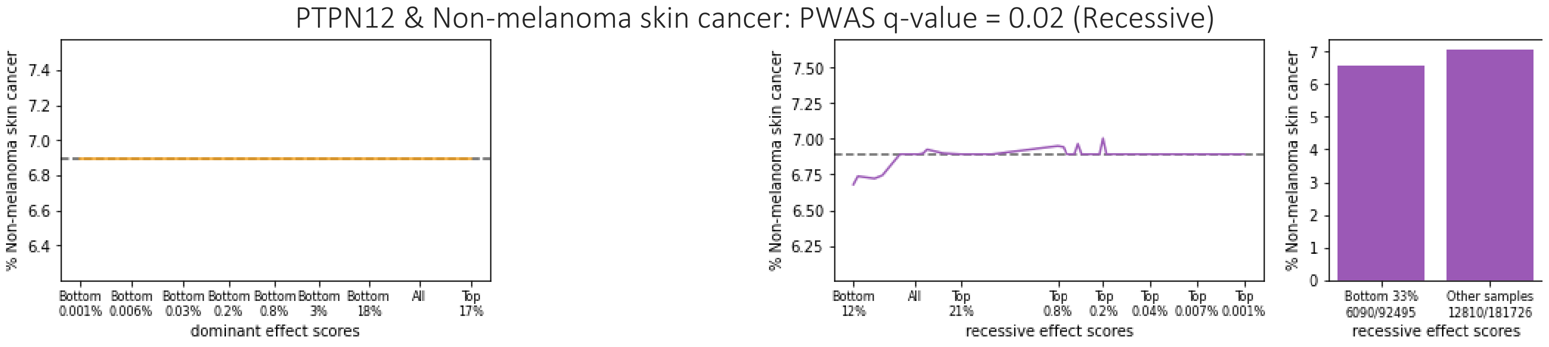
PTPN12 & Non-melanoma skin cancer: PWAS q-value = 0.02 (Recessive)
% Non-melanoma skin cancer
dominant effect scores
recessive effect scores
Bottom 33% 6090/92495
Other samples 12810/181726

RALY & Non-melanoma skin cancer: PWAS q-value = 0.005 (Dominant)

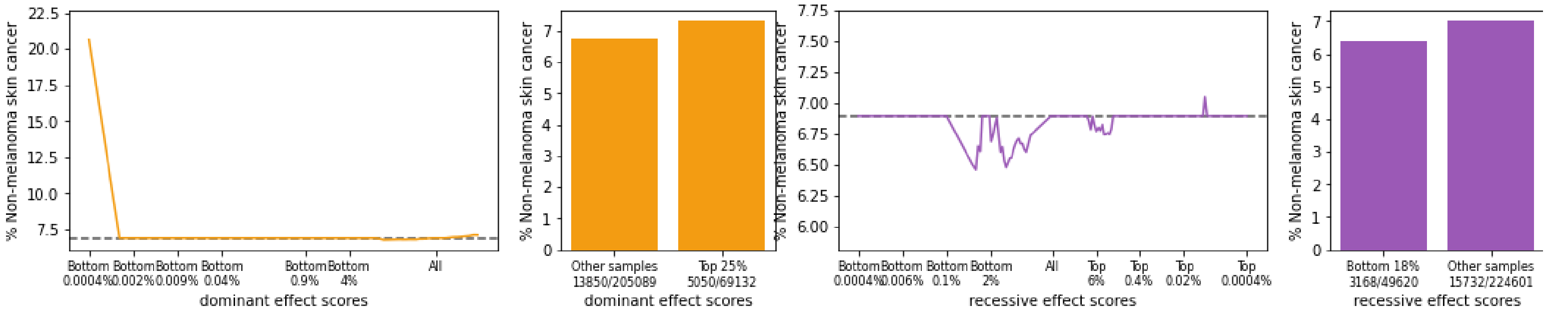

RBM17 & Non-melanoma skin cancer: PWAS q-value = 0.02 (None)

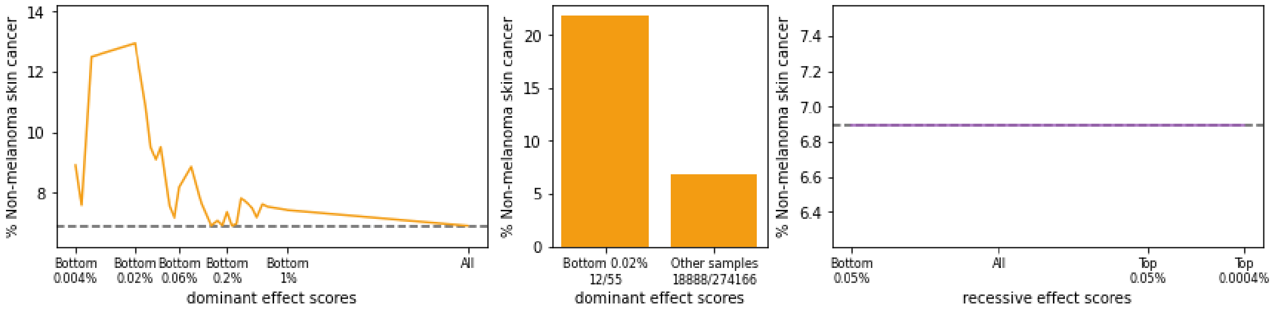

RPL13 & Non-melanoma skin cancer: PWAS q-value = 0.0004 (Dominant)

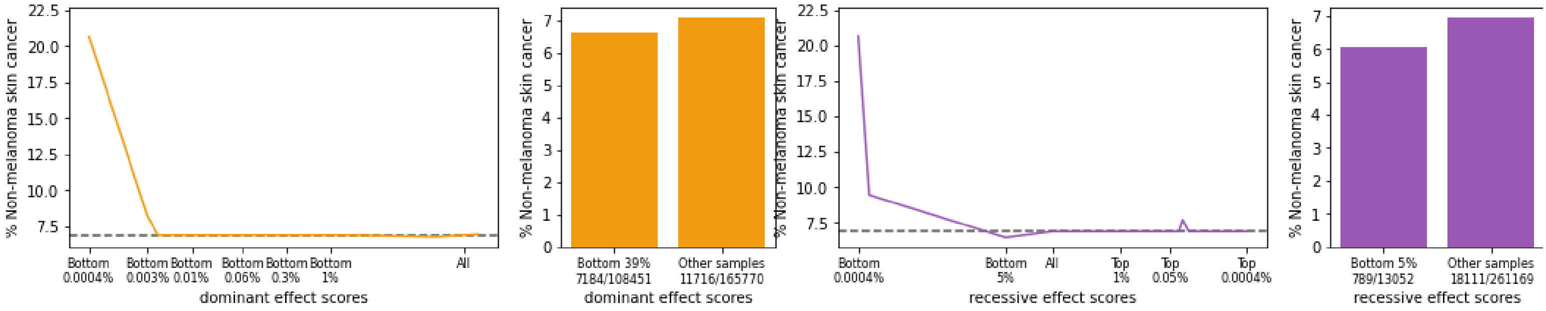

RPRD2 & Non-melanoma skin cancer: PWAS q-value = 0.008 (Recessive)

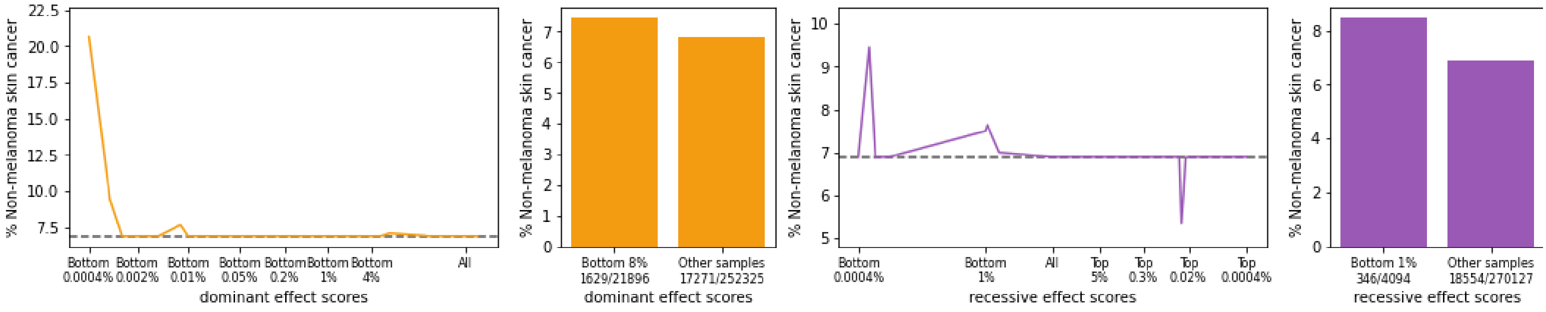

RPS6KA4 & Non-melanoma skin cancer: PWAS q-value = 0.01 (None)

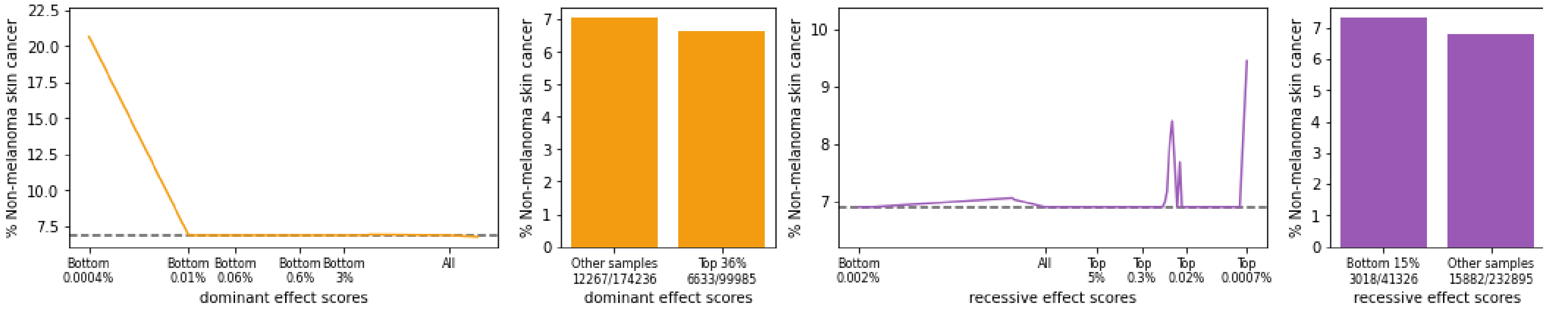

RSC1A1 & Non-melanoma skin cancer: PWAS q-value = 0.04 (None)

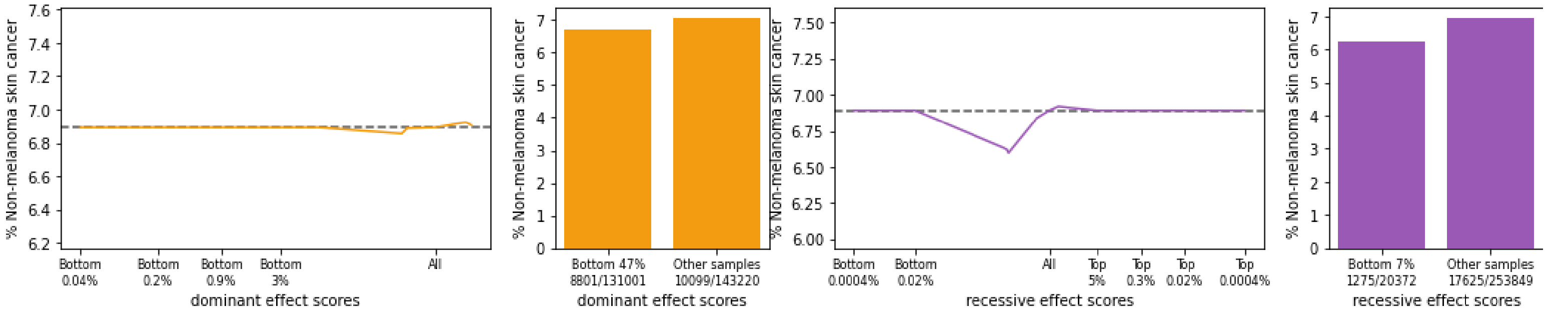

SEMA3F & Non-melanoma skin cancer: PWAS q-value = 0.008 (Recessive)

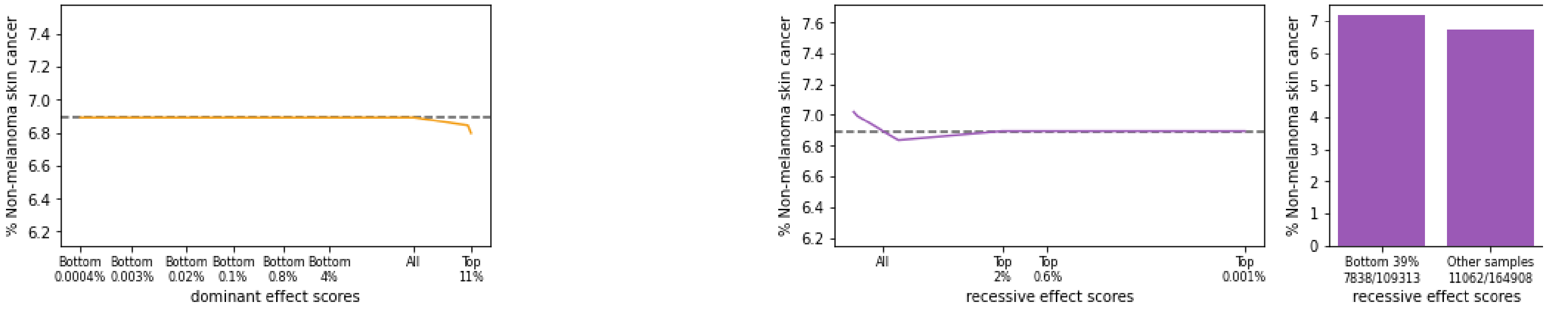

SLC45A2 & Non-melanoma skin cancer: PWAS q-value = 1e-20 (Recessive)

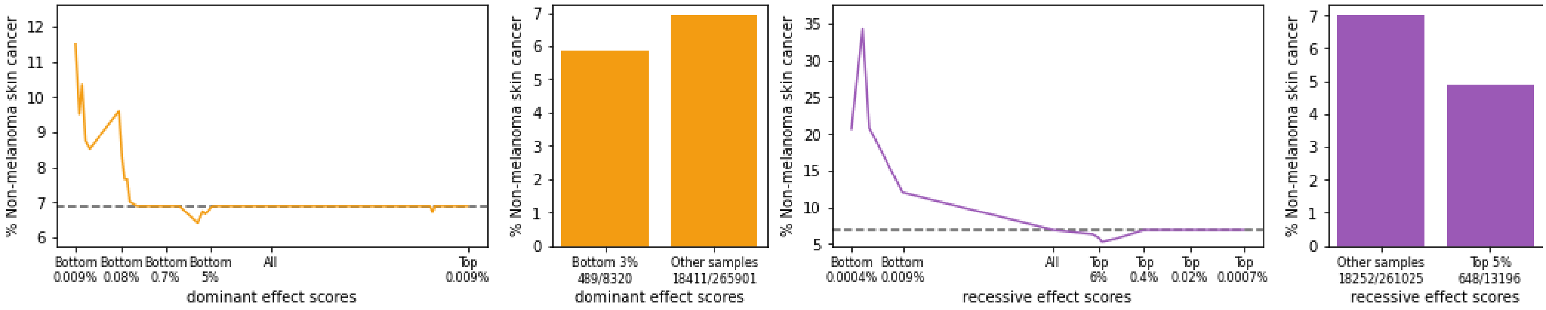

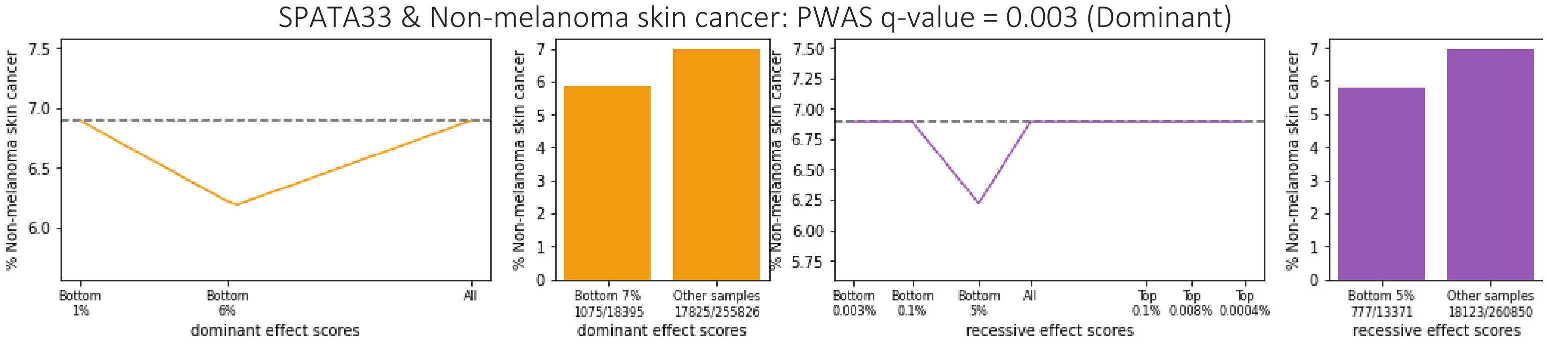
SPATA33 & Non-melanoma skin cancer: PWAS q-value = 0.003 (Dominant)
% Non-melanoma skin cancer
Bottom 1%
Bottom 6%
All
dominant effect scores
Bottom 7% 1075/18395
Other samples 17825/255826
dominant effect scores
Bottom 0.003%
Bottom 0.1%
Bottom 5%
All
Top 0.1%
Top 0.008%
Top 0.0004%
recessive effect scores
Bottom 5% 777/13371
Other samples 18123/260850
recessive effect scores

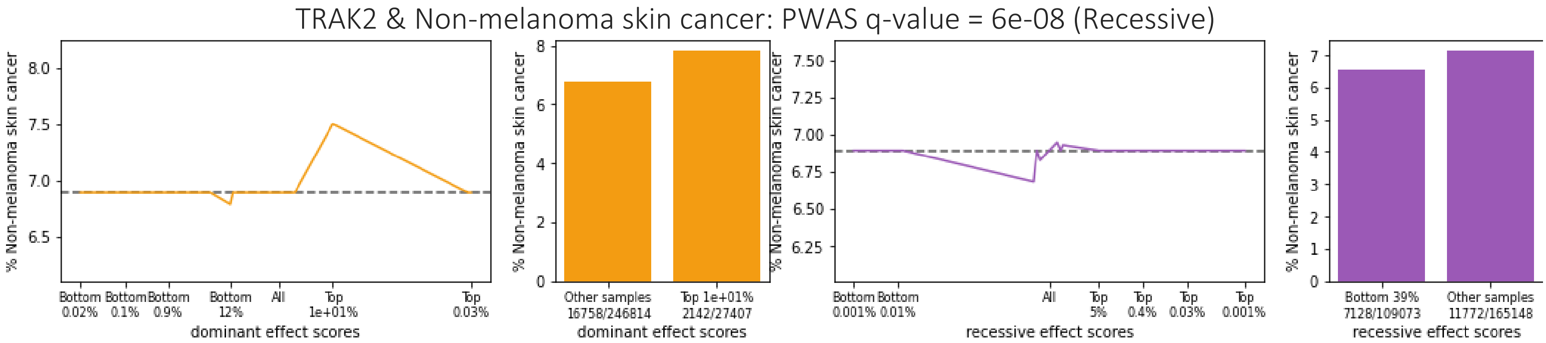
TRAK2 & Non-melanoma skin cancer: PWAS q-value = 6e-08 (Recessive)
% Non-melanoma skin cancer
Bottom 0.02%
Bottom 0.1%
Bottom 0.9%
Bottom 12%
All
Top 1e+01%
Top 0.03%
dominant effect scores
Other samples 16758/246814
Top 1e+01% 2142/27407
dominant effect scores
Bottom 0.001%
Bottom 0.01%
All
Top 5%
Top 0.4%
Top 0.03%
Top 0.001%
recessive effect scores
Bottom 39% 7128/109073
Other samples 11772/165148
recessive effect scores

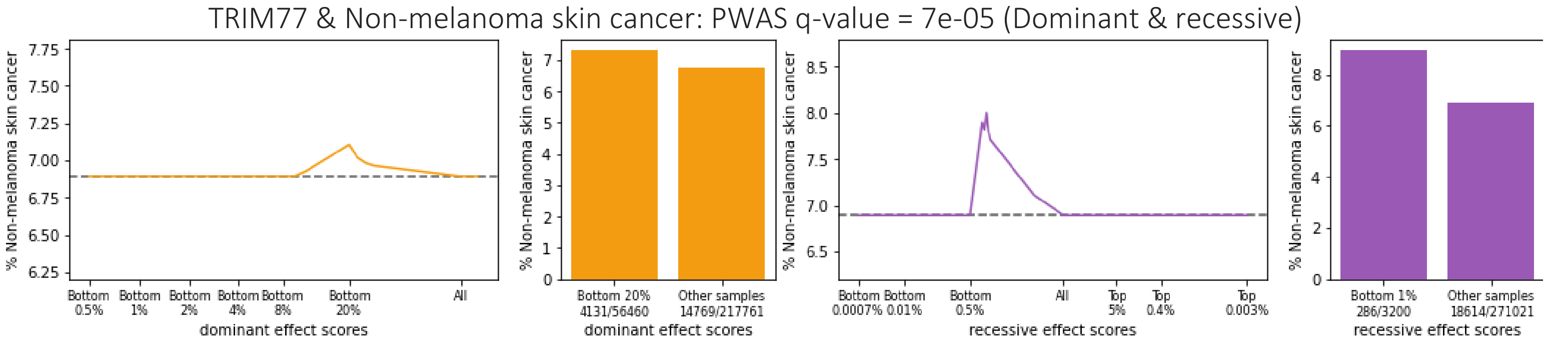
TRIM77 & Non-melanoma skin cancer: PWAS q-value = 7e-05 (Dominant & recessive)
% Non-melanoma skin cancer
Bottom 0.5%
Bottom 1%
Bottom 2%
Bottom 4%
Bottom 8%
Bottom 20%
All
dominant effect scores
Bottom 20% 4131/56460
Other samples 14769/217761
dominant effect scores
Bottom 0.0007%
Bottom 0.01%
Bottom 0.5%
All
Top 5%
Top 0.4%
Top 0.003%
recessive effect scores
Bottom 1% 286/3200
Other samples 18614/271021
recessive effect scores

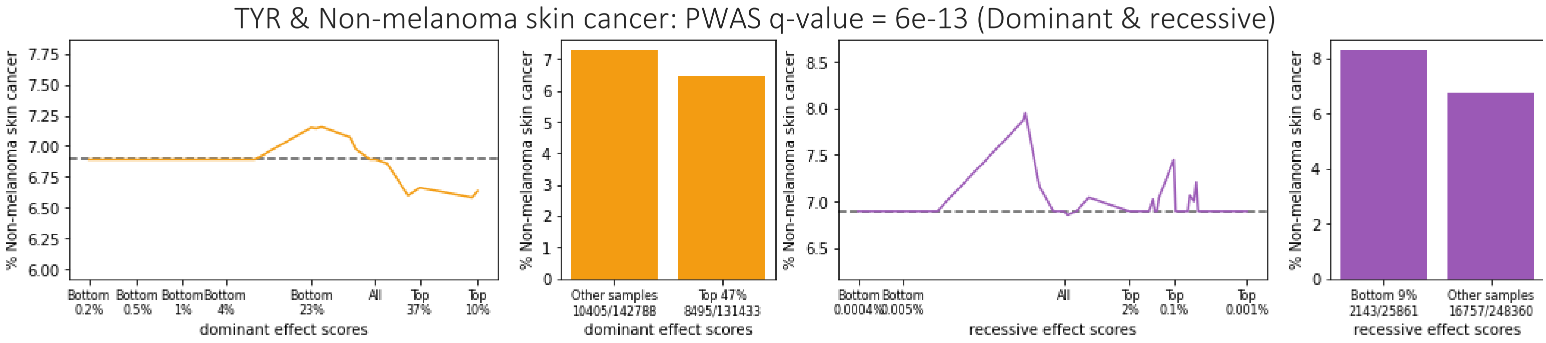
TYR & Non-melanoma skin cancer: PWAS q-value = 6e-13 (Dominant & recessive)
% Non-melanoma skin cancer
Bottom 0.2%
Bottom 0.5%
Bottom 1%
Bottom 4%
Bottom 23%
All
Top 37%
Top 10%
dominant effect scores
Other samples 10405/142788
Top 47% 8495/131433
dominant effect scores
Bottom 0.0004%
Bottom 0.005%
All
Top 2%
Top 0.1%
Top 0.001%
recessive effect scores
Bottom 9% 2143/25861
Other samples 16757/248360
recessive effect scores

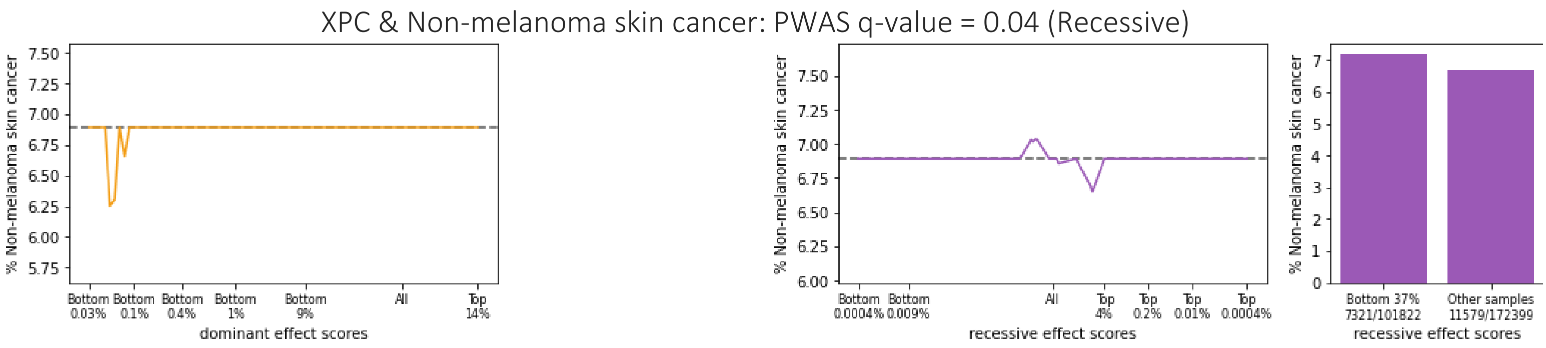
XPC & Non-melanoma skin cancer: PWAS q-value = 0.04 (Recessive)
% Non-melanoma skin cancer
dominant effect scores
recessive effect scores
Bottom 37% 7321/101822
Other samples 11579/172399

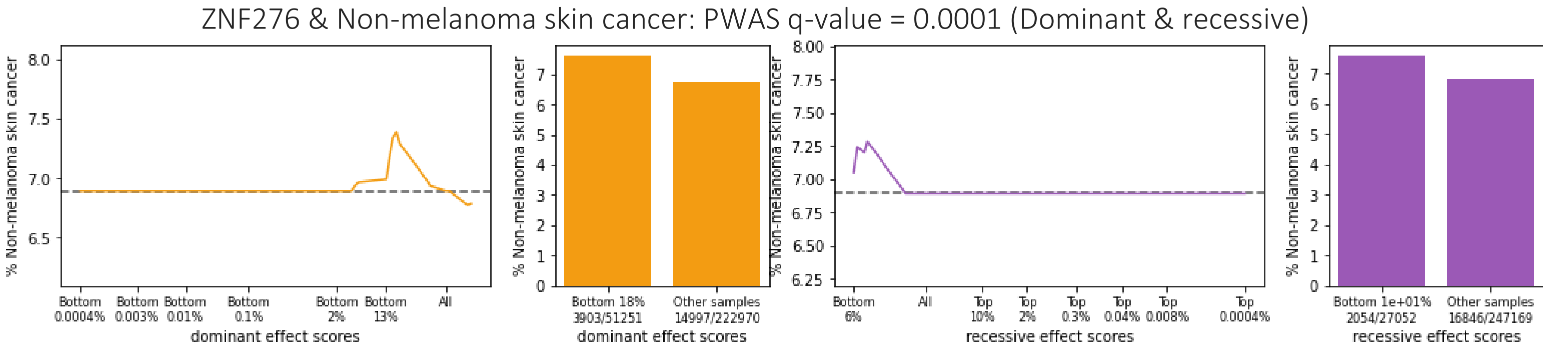
ZNF276 & Non-melanoma skin cancer: PWAS q-value = 0.0001 (Dominant & recessive)
% Non-melanoma skin cancer
dominant effect scores
Bottom 18% 3903/51251
Other samples 14997/222970
recessive effect scores
Bottom 1e+01% 2054/27052
Other samples 16846/247169

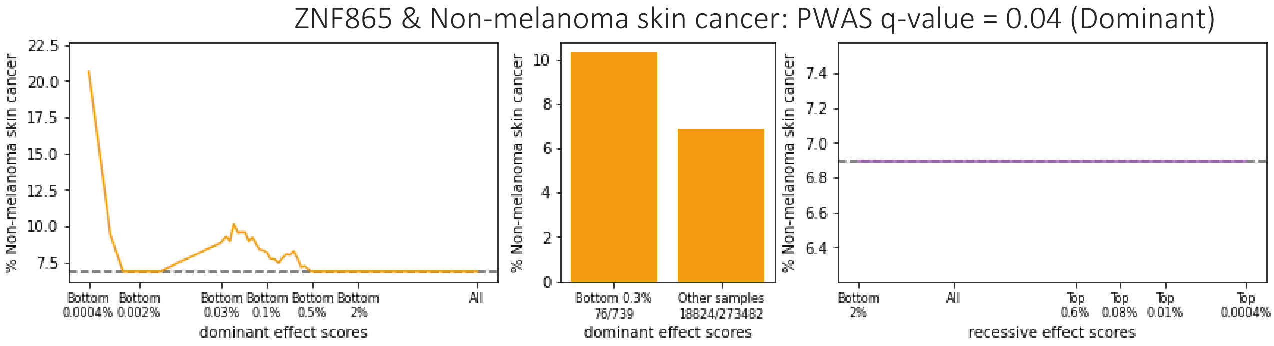
ZNF865 & Non-melanoma skin cancer: PWAS q-value = 0.04 (Dominant)
% Non-melanoma skin cancer
dominant effect scores
Bottom 0.3% 76/739
Other samples 18824/273482
recessive effect scores

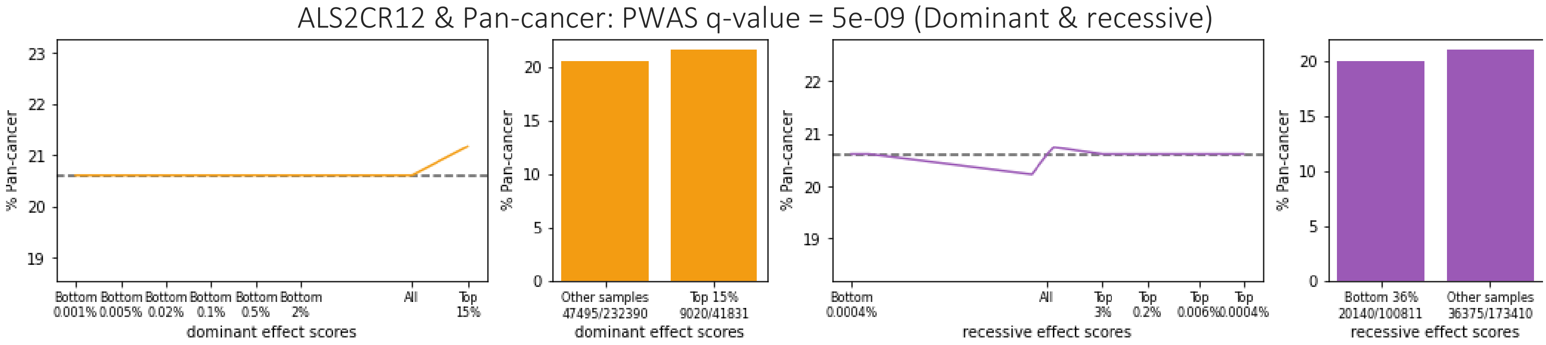
ALS2CR12 & Pan-cancer: PWAS q-value = 5e-09 (Dominant & recessive)
% Pan-cancer
dominant effect scores
Other samples 47495/232390
Top 15% 9020/41831
recessive effect scores
Bottom 36% 20140/100811
Other samples 36375/173410

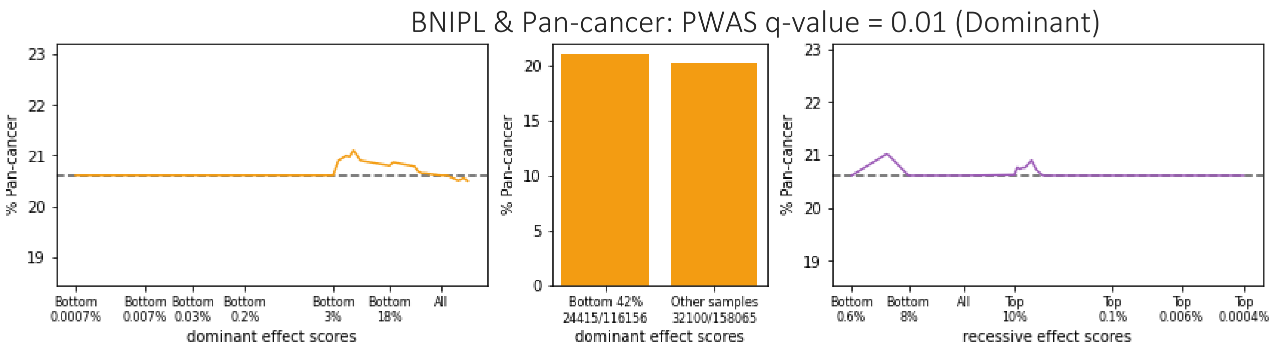
BNIPL & Pan-cancer: PWAS q-value = 0.01 (Dominant)
% Pan-cancer
Bottom 0.0007%
Bottom 0.007%
Bottom 0.03%
Bottom 0.2%
Bottom 3%
Bottom 18%
All
dominant effect scores
Bottom 42% 24415/116156
Other samples 32100/158065
dominant effect scores
Bottom 0.6%
Bottom 8%
All
Top 10%
Top 0.1%
Top 0.006%
Top 0.0004%
recessive effect scores

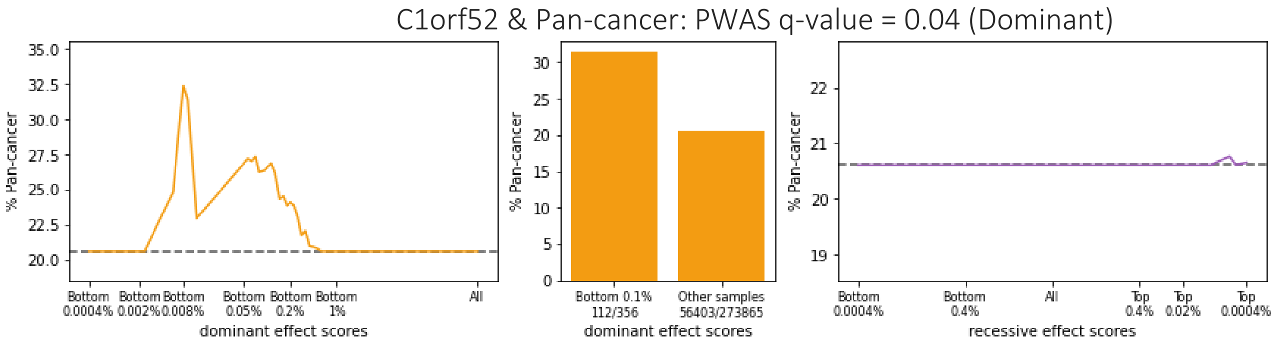
C1orf52 & Pan-cancer: PWAS q-value = 0.04 (Dominant)
% Pan-cancer
Bottom 0.0004%
Bottom 0.002%
Bottom 0.008%
Bottom 0.05%
Bottom 0.2%
Bottom 1%
All
dominant effect scores
Bottom 0.1% 112/356
Other samples 56403/273865
dominant effect scores
Bottom 0.0004%
Bottom 0.4%
All
Top 0.4%
Top 0.02%
Top 0.0004%
recessive effect scores

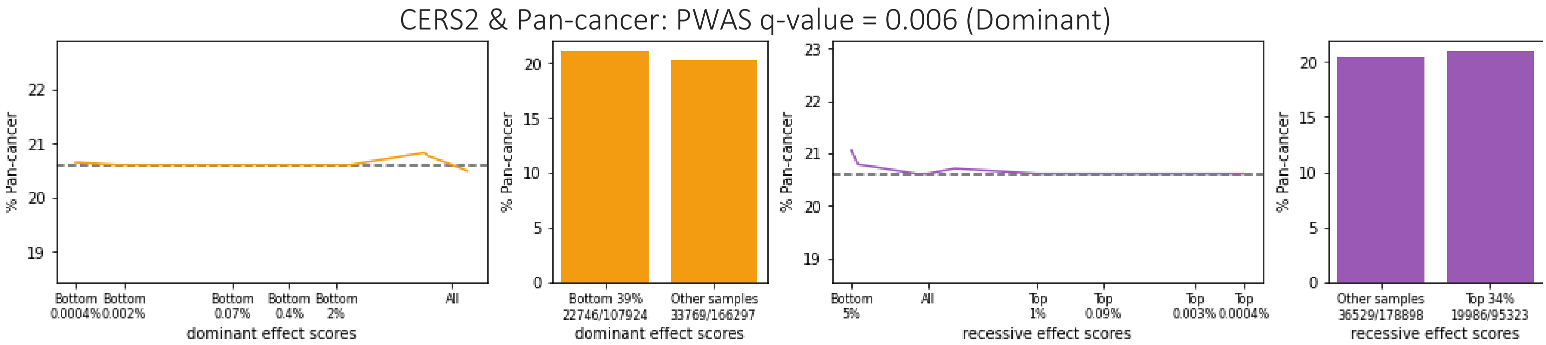
CERS2 & Pan-cancer: PWAS q-value = 0.006 (Dominant)
% Pan-cancer
Bottom 0.0004%
Bottom 0.002%
Bottom 0.07%
Bottom 0.4%
Bottom 2%
All
dominant effect scores
Bottom 39% 22746/107924
Other samples 33769/166297
dominant effect scores
Bottom 5%
All
Top 1%
Top 0.09%
Top 0.003%
Top 0.0004%
recessive effect scores
Other samples 36529/178898
Top 34% 19986/95323
recessive effect scores

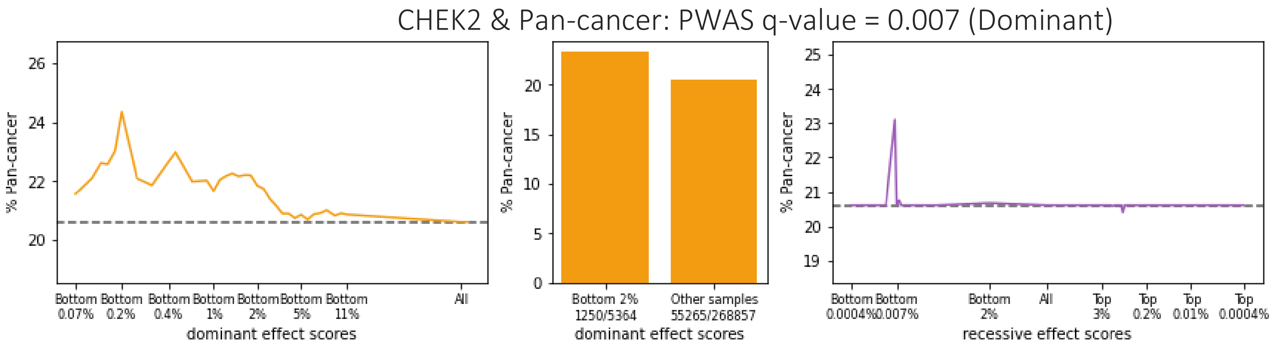
CHEK2 & Pan-cancer: PWAS q-value = 0.007 (Dominant)
% Pan-cancer
Bottom 0.07%
Bottom 0.2%
Bottom 0.4%
Bottom 1%
Bottom 2%
Bottom 5%
Bottom 11%
All
dominant effect scores
Bottom 2% 1250/5364
Other samples 55265/268857
dominant effect scores
Bottom 0.0004%
Bottom 0.007%
Bottom 2%
All
Top 3%
Top 0.2%
Top 0.01%
Top 0.0004%
recessive effect scores

CRYAA & Pan-cancer: PWAS q-value = 0.02 (Dominant)

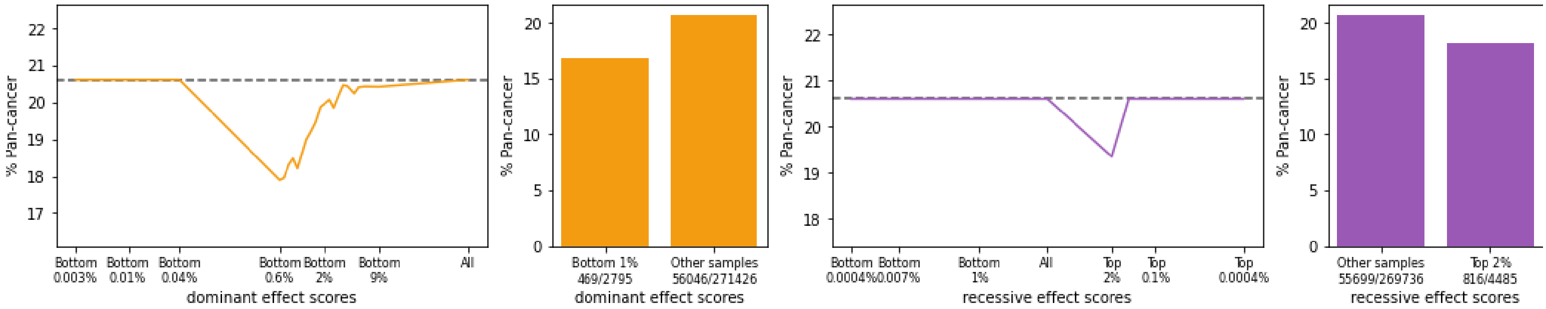

HLA-DPA1 & Pan-cancer: PWAS q-value = 0.01 (None)

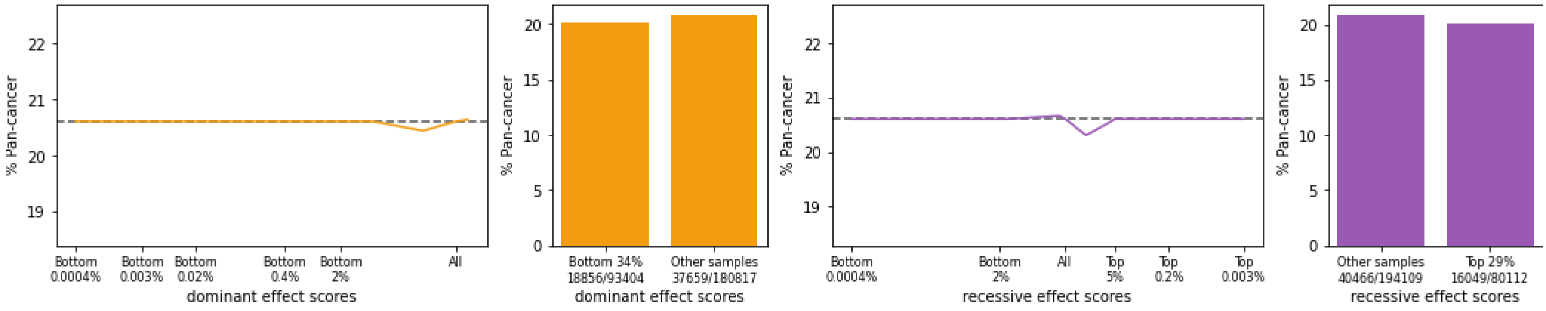

HOXB13 & Pan-cancer: PWAS q-value = 0.01 (Dominant)

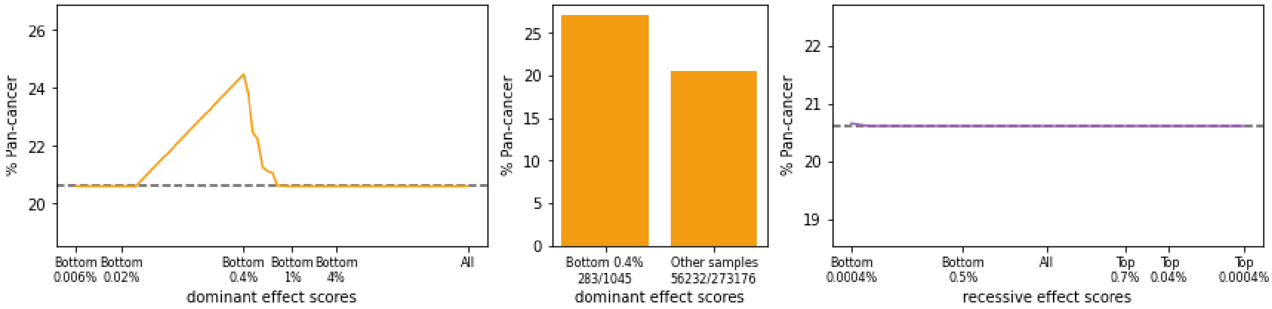

MC1R & Pan-cancer: PWAS q-value = 6e-34 (Dominant & recessive)

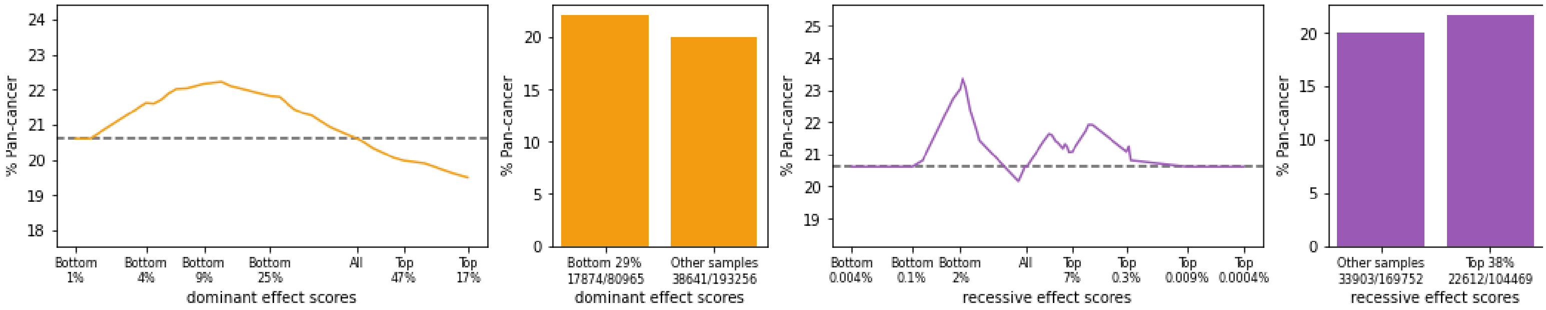

SH2B3 & Pan-cancer: PWAS q-value = 0.02 (None)

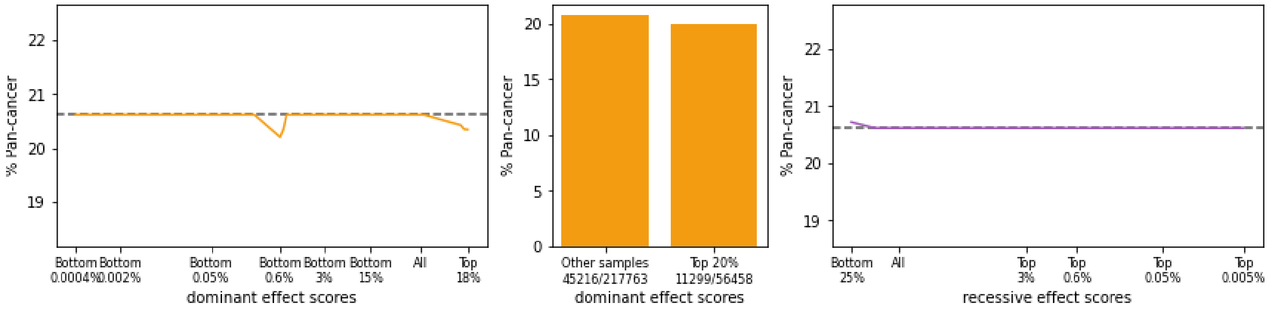

SLC45A2 & Pan-cancer: PWAS q-value = 0.02 (Recessive)

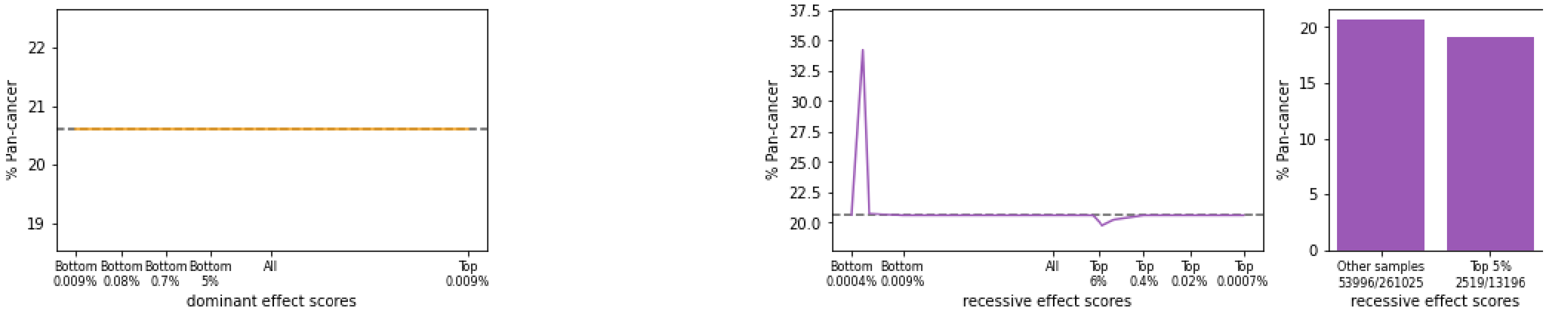

STN1 & Pan-cancer: PWAS q-value = 9e-06 (Recessive)

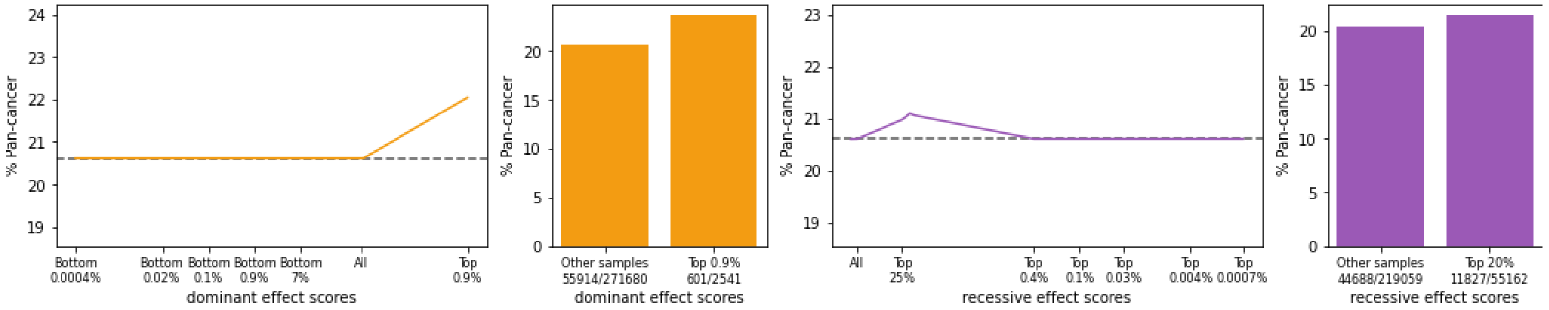

TYR & Pan-cancer: PWAS q-value = 1e-06 (Dominant & recessive)

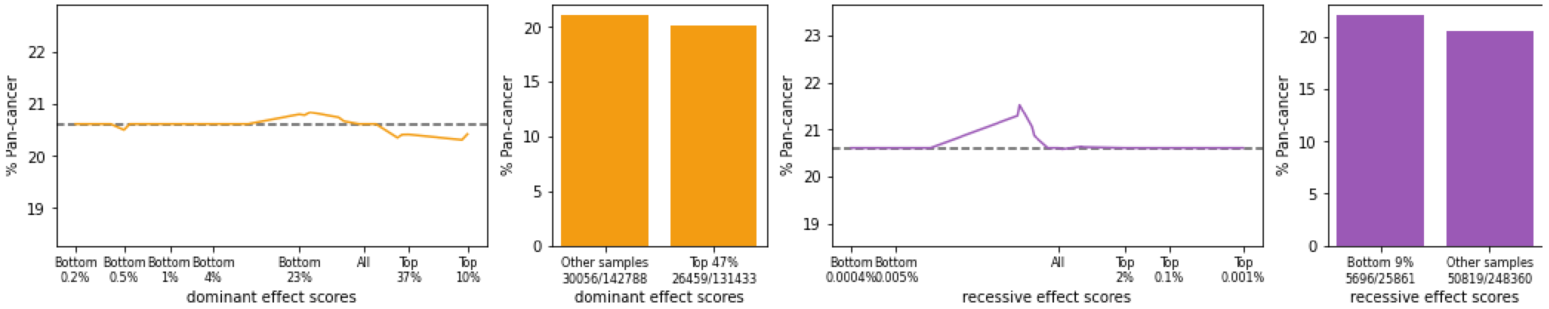

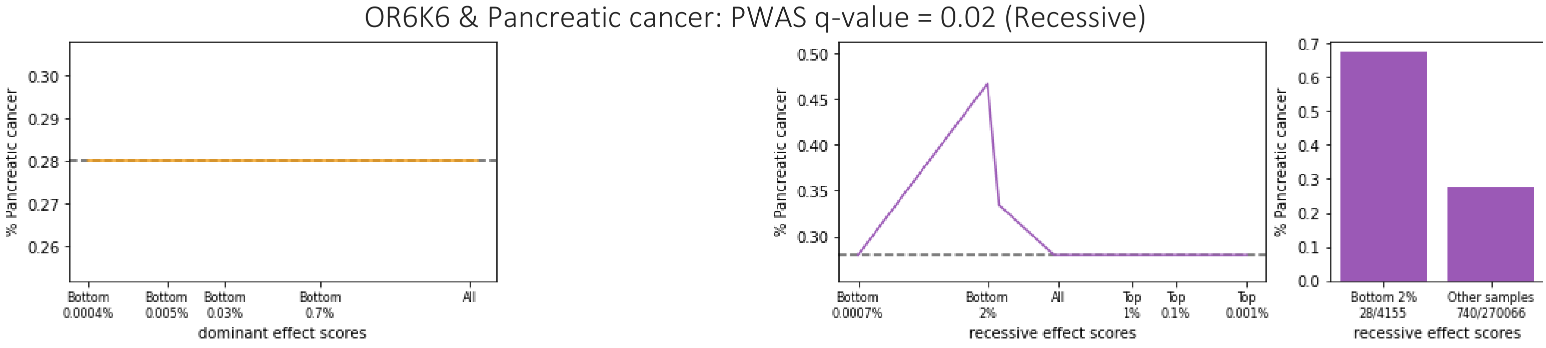
OR6K6 & Pancreatic cancer: PWAS q-value = 0.02 (Recessive)
% Pancreatic cancer
dominant effect scores
Bottom 0.0004%
Bottom 0.005%
Bottom 0.03%
Bottom 0.7%
All
recessive effect scores
Bottom 0.0007%
Bottom 2%
Top 1%
Top 0.1%
Top 0.001%
Bottom 2% 28/4155
Other samples 740/270066

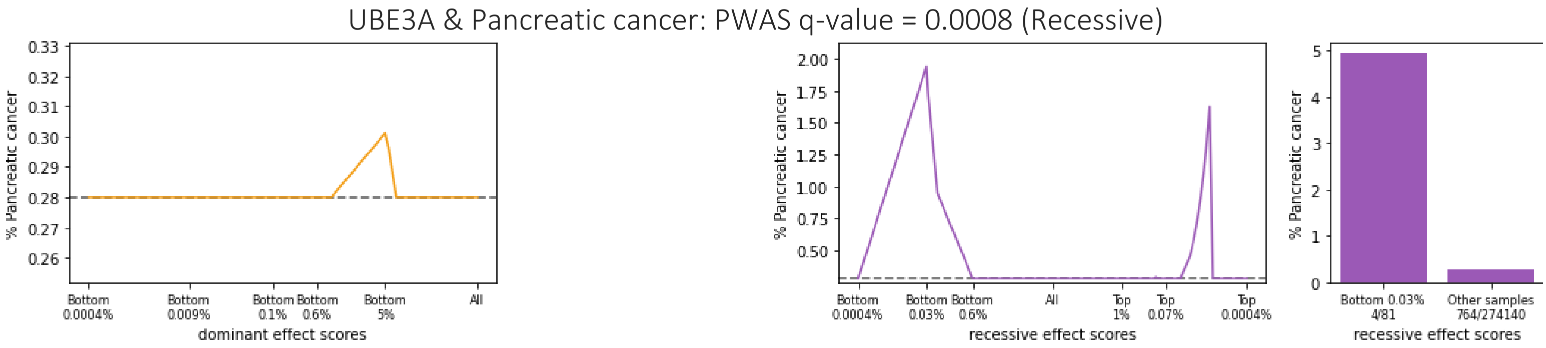
UBE3A & Pancreatic cancer: PWAS q-value = 0.0008 (Recessive)
% Pancreatic cancer
dominant effect scores
Bottom 0.0004%
Bottom 0.009%
Bottom 0.1%
Bottom 0.6%
Bottom 5%
All
recessive effect scores
Bottom 0.0004%
Bottom 0.03%
Bottom 0.6%
Top 1%
Top 0.07%
Top 0.0004%
Bottom 0.03% 4/81
Other samples 764/274140

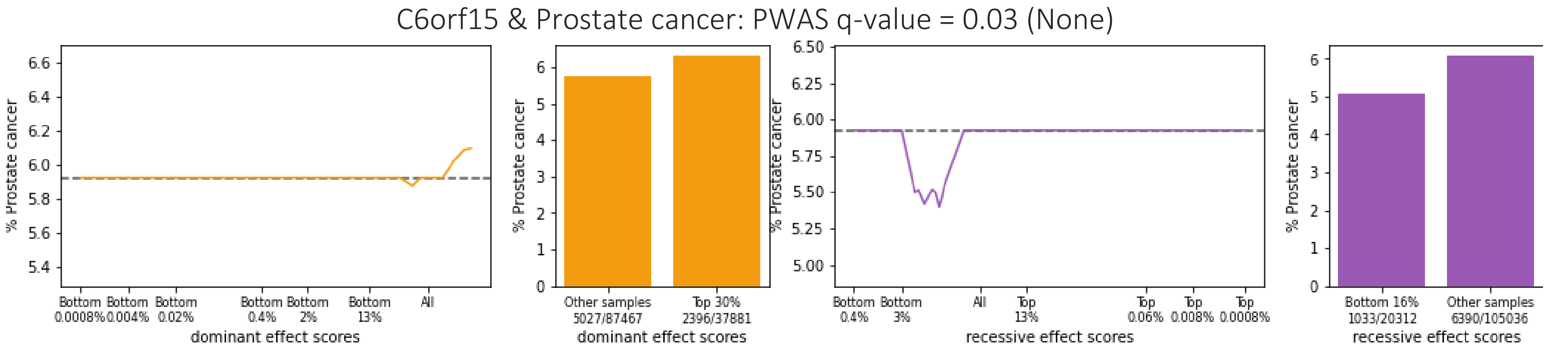
C6orf15 & Prostate cancer: PWAS q-value = 0.03 (None)
% Prostate cancer
dominant effect scores
Bottom 0.0008%
Bottom 0.004%
Bottom 0.02%
Bottom 0.4%
Bottom 2%
Bottom 13%
All
Other samples 5027/87467
Top 30% 2396/37881
recessive effect scores
Bottom 0.4%
Bottom 3%
Top 13%
Top 0.06%
Top 0.008%
Top 0.0008%
Bottom 16% 1033/20312
Other samples 6390/105036

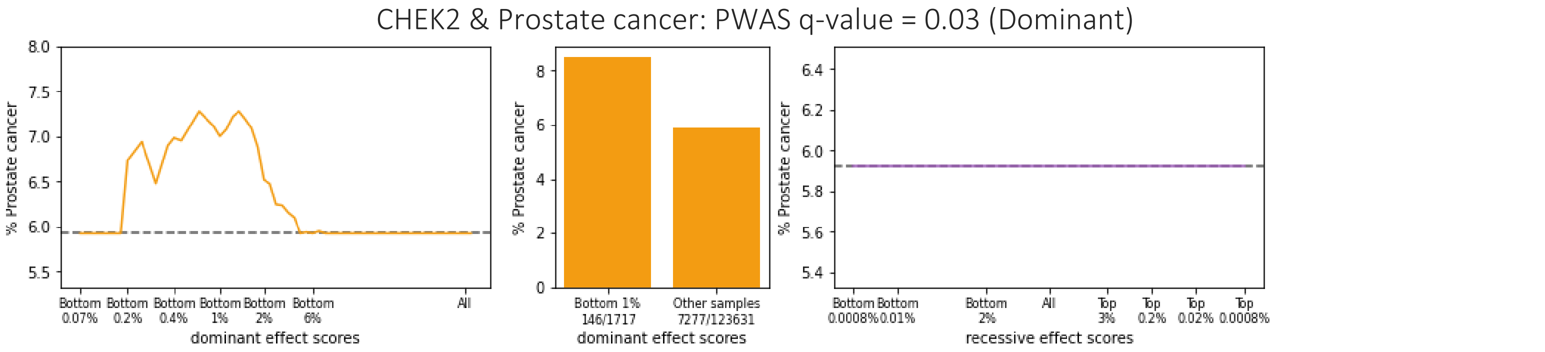
CHEK2 & Prostate cancer: PWAS q-value = 0.03 (Dominant)
% Prostate cancer
dominant effect scores
Bottom 0.07%
Bottom 0.2%
Bottom 0.4%
Bottom 1%
Bottom 2%
Bottom 6%
All
Bottom 1% 146/1717
Other samples 7277/123631
recessive effect scores
Bottom 0.0008%
Bottom 0.01%
Bottom 2%
Top 3%
Top 0.2%
Top 0.02%
Top 0.0008%

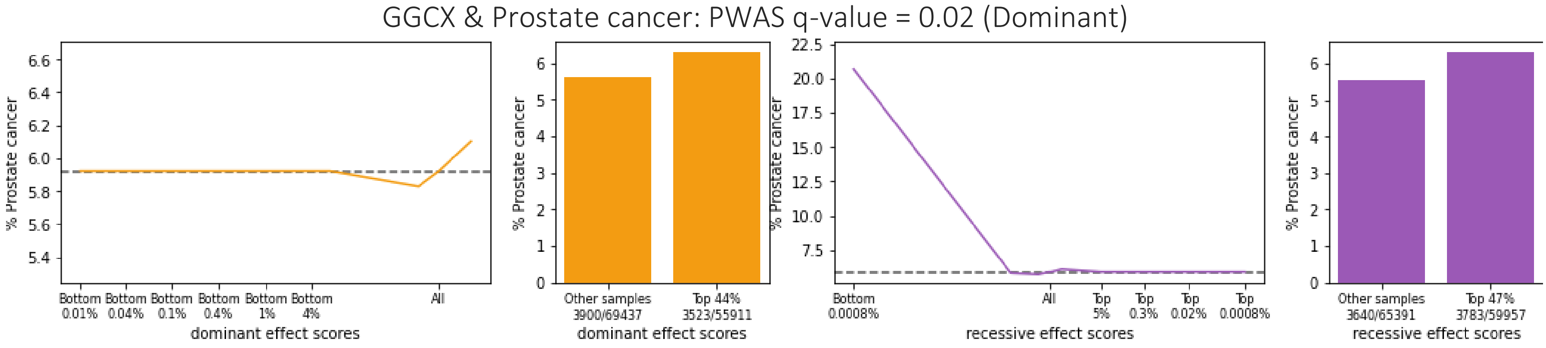
GGCX & Prostate cancer: PWAS q-value = 0.02 (Dominant)
% Prostate cancer
dominant effect scores
Other samples
3900/69437
Top 44%
3523/55911
recessive effect scores
Other samples
3640/65391
Top 47%
3783/59957

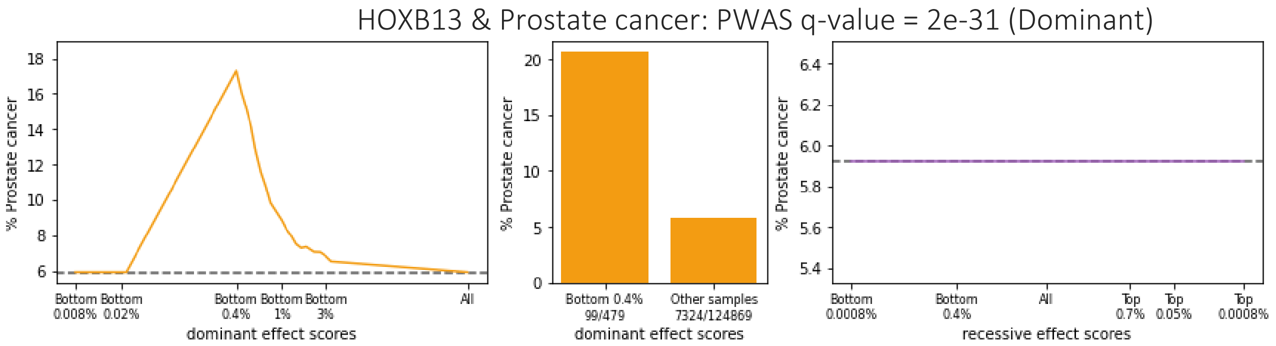
HOXB13 & Prostate cancer: PWAS q-value = 2e-31 (Dominant)
% Prostate cancer
dominant effect scores
Bottom 0.4%
99/479
Other samples
7324/124869
recessive effect scores

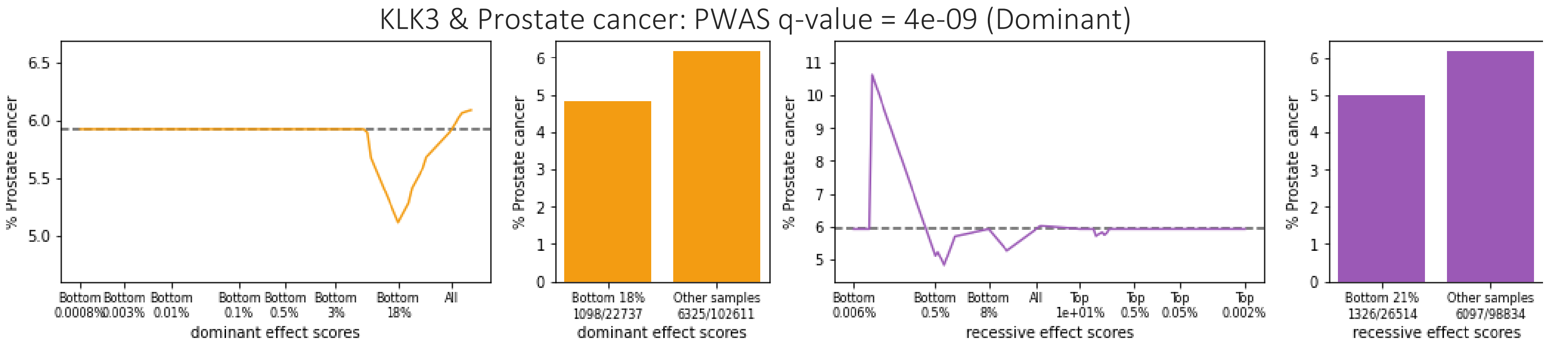
KLK3 & Prostate cancer: PWAS q-value = 4e-09 (Dominant)
% Prostate cancer
dominant effect scores
Bottom 18%
1098/22737
Other samples
6325/102611
recessive effect scores
Bottom 21%
1326/26514
Other samples
6097/98834

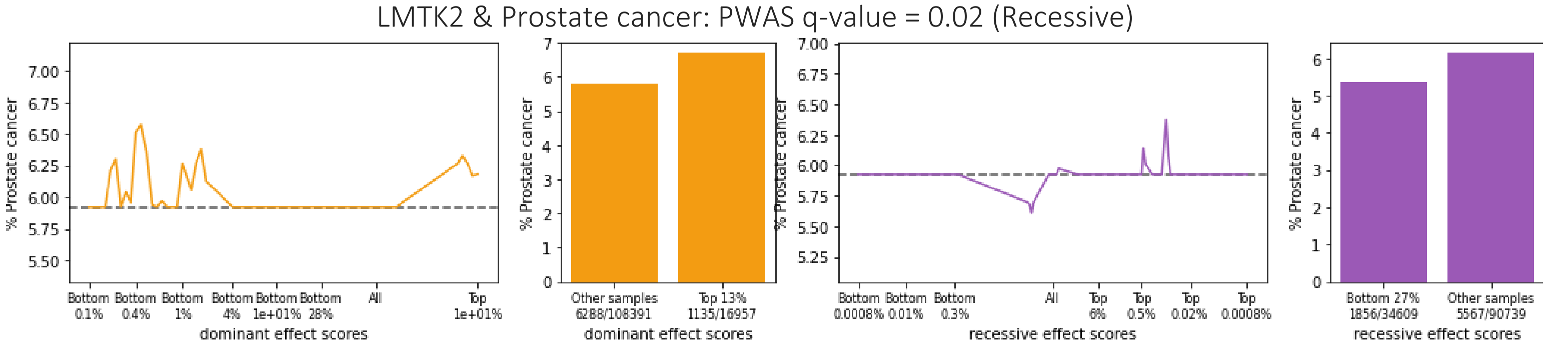
LMTK2 & Prostate cancer: PWAS q-value = 0.02 (Recessive)
% Prostate cancer
dominant effect scores
Other samples
6288/108391
Top 13%
1135/16957
recessive effect scores
Bottom 27%
1856/34609
Other samples
5567/90739

POU5F1B & Prostate cancer: PWAS q-value = 1e-22 (Dominant & recessive)

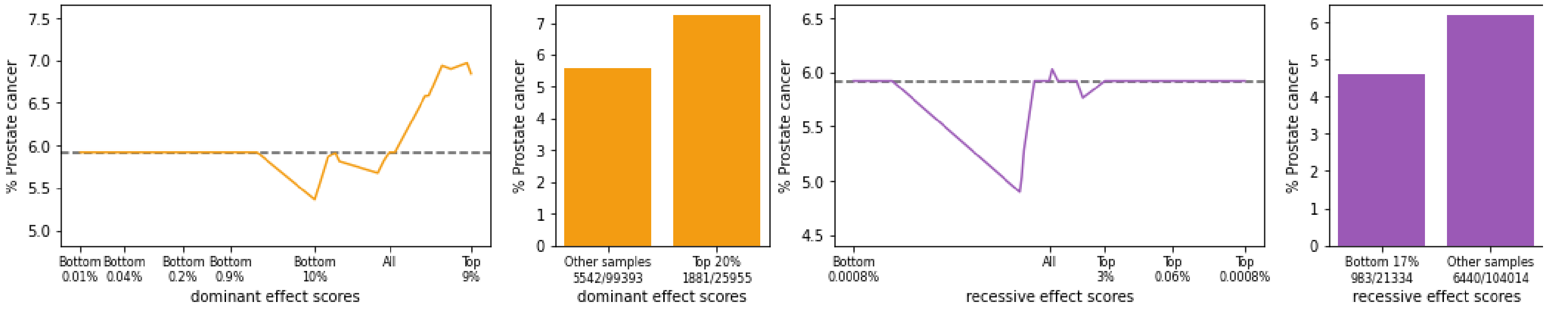

SLC2A4RG & Prostate cancer: PWAS q-value = 0.02 (Recessive)

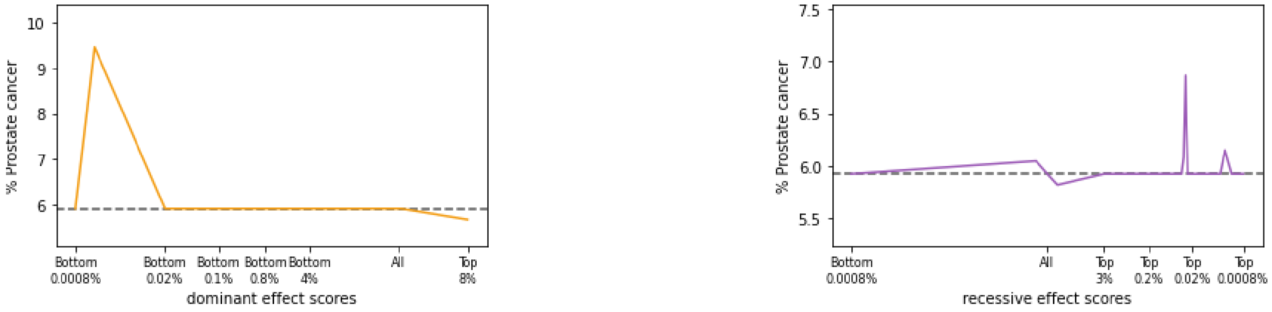

Supplement: Supplementary file 1 — Supplementary Figures. [file 41598_2021_94252_MOESM1_ESM.pdf]
